# Global, regional, and national burden of leukemia: Epidemiological trends analysis from 1990 to 2021

**DOI:** 10.1371/journal.pone.0325937

**Published:** 2025-06-26

**Authors:** Chengjun Hu, Weifeng Chen, Ping Zhang, Tongping Shen, Maozhong Xu

**Affiliations:** 1 Department of Hematology, The Affiliated Jiangyin Hospital of Nantong University, Jiangyin, Jiangsu 214400, China; 2 School of Information Engineering, Anhui University of Chinese Medicine, Hefei, Anhui 230012, China; Instituto Nacional de Salud Publica, MEXICO

## Abstract

Background: Leukemia is a prevalent form of cancer that encompasses four primary subtypes, posing significant health risks. Gaining insights into the global epidemiology of leukemia and its subtypes is crucial for effective resource allocation, clinical guidance, and scientific inquiry. Methods: From 1990 to 2021, we analyzed age-standardized rates (ASR) trends across 204 countries and territories, utilizing the estimated annual percentage change (EAPC). Results: In 2021, there were approximately 460,000 leukemia cases, 320,000 deaths, and 10.98 million disability-adjusted life years (DALYs). Globally, age-standardized incidence rates (ASIR), mortality rates (ASMR), and DALY rates are on the decline, while age-standardized prevalence rates (ASPR) are rising in high social demographic index (SDI) regions. Men experience a higher leukemia burden compared to women. By subtype, acute myeloid leukemia (AML), chronic myeloid leukemia (CML), and chronic lymphocytic leukemia (CLL) predominantly impact older individuals, while acute lymphoblastic leukemia (ALL) primarily affects children. Notably, leukemia ASIR and ASDR reductions are observed in countries with high human development index (HDI). Conclusion: Although there has been a marked decrease in global leukemia incidence, mortality, and DALYs over the past 31 years, the rise in new cases due to population growth suggests an increasing overall disease burden by 2050. Furthermore, the impact of leukemia varies significantly by region and country, highlighting the urgent need for innovative and personalized prevention and treatment approaches to mitigate this global health issue.

## Introduction

Leukemia is a prevalent type of cancer primarily marked by an abnormal increase in white blood cells in the blood or bone marrow [1]. According to the statistics of 2018, leukemia has become one of the leading causes of death from malignant tumors worldwide [2].

According to the fourth edition of the World Health Organization (WHO) classification, leukemia is generally divided into two categories: myeloid and lymphoid leukemia. The development of leukemia involves the over proliferation of blood cells, leading to the formation of malignant tumors and bone marrow failure. Leukemic cells, which can be mature or precursor cells of different lineages, are classified into four main subtypes: AML, ALL, CML, and CLL [3].

Leukemia is a multifactorial disease caused by the interaction of genetic and environmental factors, and its exact etiology has not yet been fully elucidated. The incidence of leukemia usually increases with age and peaks especially in the elderly population [4]. The incidence of leukemia is approximately twice as high in men as in women [5].

Among the various types of leukemia, CML is a clonal disease of hematopoietic stem cells primarily resulting from reciprocal chromosomal translocations [6]. Conversely, CLL is recognized as one of the most genetically predisposed forms of hematologic cancer, with approximately 10% of affected individuals reporting a family history of the condition [7]. Environmental influences are also critical in the onset and advancement of leukemia; for instance, ALL and CLL are notably impacted by exposure to insecticides, ionizing radiation, and certain infections [8, 9]. Additionally, around 10% of patients with AML have undergone cytotoxic chemotherapy or ionizing radiation prior to their diagnosis, as these interventions are typically employed in the treatment of other primary cancers [10].

Other possible risk factors for leukemia include smoking, elevated body mass index, and occupational exposure to substances like benzene and formaldehyde [11]. Over the past few decades, there has been significant advancement in treating hematologic malignancies, especially with targeted therapies such as tyrosine kinase inhibitors (TKIs) [12, 13]. Nevertheless, the outlook for leukemia patients remains grim, and the disease continues to pose a significant threat to public health. Data from 2020 indicate that leukemia ranks as the 15th most prevalent cause of cancer globally [14]. Moreover, it is the most frequently diagnosed cancer in children under the age of 5 and carries the highest mortality rate, imposing a substantial burden on individuals, families, and healthcare systems [15].

Given the rapid evolution of leukemia-related conditions and the potential variations in regional and demographic distribution, conducting epidemiological studies on leukemia is essential for policymakers to allocate healthcare resources effectively. In 2015, four significant leukemia subtypes were recognized in the Global Burden of Disease (GBD) framework, enhancing understanding of the current trends and status of the disease [16]. This report utilizes findings from the GBD 2021 study to thoroughly analyze global leukemia patterns and trends from 1990 to 2021, identify risk factors that could inform targeted intervention strategies, and apply a linkage-point regression model for projecting trends up to 2050.

## Methodology

### Data sources

Data related to leukemia were sourced from the most recent edition of the Global Burden of Disease (GBD) study, using the Global Health Data Exchange (GHDx) query tool (http://ghdx.healthdata.org/gbd-results-tool). We gathered statistical information pertaining to morbidity, mortality, and disability-adjusted life years (DALYs) for diseases and injuries from 1990 to 2021. This dataset encompasses four primary leukemia subtypes: AML, ALL, CLL, and CML.

The Socio-Demographic Index (SDI) is calculated by integrating factors such as fertility rate, the average educational attainment of individuals aged 15 and older, and per capita income rankings. It provides an insight into a country’s overall social development, ranging from 0 to 1 and classified into five categories: low, low-moderate, moderate, moderate-high, and high. The world is further divided into 21 geographic regions based on specific geographic characteristics, including East Asia, South Asia, and Western Europe, and the data encompasses 204 countries and territories, which include nations like China, India, and France.

The methodology used for the global burden of disease estimation in 2021 aligns with approaches detailed in prior studies [17, 18]. The Human Development Index (HDI), serving as a comprehensive indicator of human development levels and health resource availability, was obtained from the United Nations Development Program (UNDP: http://hdr.undp.org/en/data). Additionally, the age-standardized rate (ASR) is a valuable measure that accounts for variations in age distribution, effectively adjusting for changes in population structure and size [19].

### Statistical analysis

We used EAPC to describe the magnitude of change in ASR trends, including ASIR, ASMR, and age-standardized DALY rate, to quantify trends in the leukemia burden and its burden for the four subtypes [20].

The age-standardized rate is calculated per 100,000 population using the following formula:

$$ASR=\frac{\sum_{i=1}^{A}a_{i}w_{i}}{\sum_{i=1}^{A}w_{i}}\times 100000$$ (1)

Where ASR is the age-standardized rate, is the age-specific rate for the *i*_*th*_ age group, *w*_*i*_ is the number of people in the standard population corresponding to the *i*_*th*_ age group, and A is the number of age groups.

y=α+βx+ε (2)

EAPC=100×(exp(β)−1) (3)

The trend in age-standardized rates was evaluated by analyzing the Estimated Annual Percentage Change (EAPC) and its 95% confidence interval. A positive EAPC value and a positive lower limit of the 95% confidence interval suggest an upward trend in age-standardized rates over time. In contrast, a negative EAPC value and a negative upper limit of the 95% confidence interval suggest a downward trend in these rates.

To investigate the factors affecting EAPC, we conducted a Pearson correlation analysis to examine the relationship between EAPC and the Human Development Index (HDI) for 2021. Additionally, we analyzed the age distribution of the four leukemia subtypes in five-year intervals. We evaluated age-specific potential risk factors associated with leukemia-related mortality and disability-adjusted life years (DALYs). This comprehensive approach allows for a better understanding of the interplay between socioeconomic development indicators and leukemia trends and the identification of critical age-related risk factors in different population segments. To project the global leukemia burden to 2050, we first calculated age-specific incidence rates for each age group (0-4 to >95 years) for each five years period between 1990 and 2021. Subsequently, connect-the-dots regression models were used to identify years with significant changes in age-standardized incidence rate (ASR) trends for each age group. By fitting a connect-the-dots model, we analyzed temporal trends from 1990 to 2021 and set up to six connect-the-dots as options in the model.

Additionally, we computed the expected age-specific incidence rates by multiplying these rates with the world population projections for each age group, which can be accessed at (https://population.un.org/wpp/downloads). This allowed us to estimate the number of leukemia cases in each age group. Subsequently, the direct age-standardized rate (ASR) was determined by dividing the total number of cases by the total population within each age group. For statistical analysis, p-values less than 0.05 were considered significant, and all tests were conducted as two-sided. This methodology ensures a robust evaluation of leukemia incidence across diverse age groups while accounting for population dynamics.

### Data visualization

We used packages including ggmap, ggplot2, tidyr, reshape and RcolorBrewer for data visualization. The dplyr package was used for data cleaning. The ggmap was used to visualize the leukemia EAPC for 204 countries and regions. All statistical analyses and visualizations were conducted using R software (version 4.3.0).

## Results

### Leukemia burden at global and regional levels

**Fig 1:**
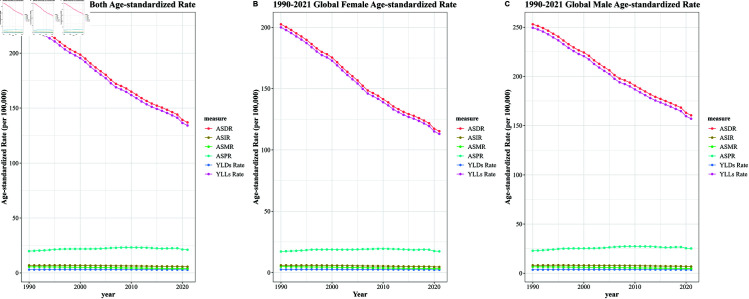
Trends in overall global leukemia burden, 1990-2021.

The global burden of new leukemia cases has risen from 311,648 (95% uncertainty interval (UI): 278,620 to 343,177) in 1990 to 461,423 (95% UI: 397,548 to 504,397) in 2021, with projections indicating an increase to 519,540 cases by 2050. Interestingly, the overall age-standardized incidence rate (ASIR) displayed a declining trend, with a mean annual decrease of 0.88% (EAPC = -0.88; 95% CI: -2.02 to -0.28). See Table 1 and Fig 1 .

**Table 1:** Global leukemia incidence and EAPC, 1990-2021.

	1990		2021		1990-2021
	Incident cases	ASIR per 100,000	Incident cases	ASIR per 100,000	EAPC
	No. *10^2^ (95% UI)	No. (95% UI)	No. *10^2^ (95% UI)	No.(95% UI)	No. (95% CI)
Overall	3116.48 [2786.2-3431.77]	6.89 [6.22-7.49]	4614.23 [3975.48-5043.97]	5.63 [4.83-6.17]	-0.88 [-2.02 to 0.28]
Sex					
Female	1405.74 [1183.45-1574]	5.93 [5.01-6.56]	1977.1 [1647.67-2178.65]	4.6 [3.81-5.06]	-1.05 [-2.23 to 0.14]
Male	1710.74 [1437.25-1988.96]	8.08 [6.99-9.18]	2637.12 [2118.45-3023.4]	6.84 [5.51-7.81]	-0.77 [-1.89 to 0.37]
Socio-demographic index					
High SDI	1026.68 [983.77-1052.02]	10.09 [9.69-10.32]	1491.28 [1366.51-1570.86]	8.35 [7.72-8.81]	-0.74 [-2.11 to 0.65]
High-middle SDI	779.76 [687.7-842.91]	7.75 [6.85-8.39]	1147.57 [948.68-1291.67]	7.44 [5.88-8.62]	-0.45 [-1.69 to 0.81]
Middle SDI	818.07 [671.37-957.97]	5.52 [4.61-6.39]	1179.19 [940.53-1357.13]	4.72 [3.73-5.39]	-0.48 [-1.75 to 0.8]
Low-middle SDI	347.25 [273.78-431.56]	3.68 [3.03-4.47]	551.87 [458.09-655.27]	3.37 [2.8-4.03]	-0.19 [-1.48 to 1.11]
Low SDI	141.65 [97.91-192.5]	3.6 [2.71-4.53]	239.66 [170.75-298.71]	3.05 [2.19-3.83]	-0.65 [-1.75 to 0.47]
Region					
Andean Latin America	17.64 [15.02-21.94]	5.47 [4.7-6.69]	36.1 [26.71-44.77]	5.78 [4.28-7.17]	0.41 [-1.06 to 1.9]
Australasia	23.98 [22.86-25.1]	10.64 [10.14-11.16]	51.95 [46.72-56.62]	10.61 [9.73-11.57]	-0.39 [-1.78 to 1.02]
Caribbean	17.77 [15.59-20.4]	5.75 [5.16-6.47]	25.9 [22.22-30.01]	5.14 [4.4-6.01]	-0.3 [-1.34 to 0.75]
Central Asia	30.35 [28.8-31.92]	4.84 [4.62-5.08]	32.61 [28.69-36.58]	3.58 [3.17-3.99]	-1.05 [-2.26 to 0.18]
Central Europe	93.11 [89.64-96.94]	6.63 [6.39-6.9]	146.23 [132.88-157.57]	7.24 [6.56-7.83]	-0.35 [-1.63 to 0.95]
Central Latin America	70.74 [68.69-73.11]	5.04 [4.9-5.17]	128.07 [114.87-143.98]	5.13 [4.6-5.79]	-0.28 [-1.67 to 1.14]
Central Sub-Saharan Africa	8.03 [5.95-10.96]	2.05 [1.46-2.65]	17.34 [11.16-23.96]	2.04 [1.25-2.98]	0.05 [-1.84 to 1.98]
East Asia	778.08 [597.54-926.27]	7.04 [5.46-8.44]	1087.37 [779.68-1355.87]	7.14 [4.94-8.93]	-0.18 [-1.75 to 1.41]
Eastern Europe	163.39 [159.34-167.48]	6.48 [6.31-6.66]	181.45 [167.27-195.81]	5.95 [5.49-6.42]	-0.65 [-1.77 to 0.48]
Eastern Sub-Saharan Africa	63.82 [43.03-88.65]	4.45 [3.33-5.75]	104.63 [71.37-142.37]	3.71 [2.61-5.16]	-0.86 [-2.24 to 0.55]
High-income Asia Pacific	114.39 [109.17-120.49]	6.41 [6.06-6.83]	170.08 [150.14-183.98]	5.47 [4.92-5.9]	-0.63 [-2.14 to 0.91]
High-income North America	444.76 [422.93-456.58]	13.41 [12.83-13.74]	582.28 [531.23-608.76]	9.8 [9.1-10.21]	-1.04 [-2.7 to 0.64]
North Africa and Middle East	163.64 [126.89-199.66]	6.46 [5.04-7.69]	309.96 [227.54-364.61]	6.12 [4.51-7.17]	0.04 [-0.79 to 0.88]
Oceania	1.83 [1.05-2.47]	3.97 [2.27-5.5]	3.7 [2.24-5]	3.45 [2.11-4.78]	-1.75 [-4.35 to 0.92]
South Asia	289.65 [224.81-355.98]	3.33 [2.66-3.99]	457.94 [374.17-562.37]	2.85 [2.32-3.51]	-0.45 [-1.59 to 0.71]
Southeast Asia	190.7 [151.98-238.18]	5.18 [4.2-6.36]	307.64 [248.82-366.35]	4.62 [3.76-5.53]	-0.56 [-2.07 to 0.98]
Southern Latin America	28.9 [27.91-29.85]	6.09 [5.88-6.29]	41.12 [38.19-44.2]	5.21 [4.85-5.6]	-0.59 [-1.73 to 0.56]
Southern Sub-Saharan Africa	11.3 [8.46-13.32]	3.19 [2.3-3.87]	23.89 [17.13-27.9]	3.78 [2.68-4.38]	0.37 [-2.1 to 2.9]
Tropical Latin America	55.81 [53.97-57.78]	4.64 [4.46-4.77]	104.61 [98.68-109.24]	4.31 [4.06-4.52]	-0.41 [-1.66 to 0.86]
Western Europe	527.84 [506.18-542.83]	10.66 [10.3-10.96]	755.73 [685.76-808.03]	9.6 [8.91-10.18]	-0.54 [-1.9 to 0.84]
Western Sub-Saharan Africa	20.74 [14.87-26.45]	1.17 [0.86-1.41]	45.66 [25.36-60.8]	1.17 [0.7-1.49]	-0.02 [-1.7 to 1.69]

Additionally, when analyzing the data based on the SDI, a similar decreasing trend in ASIR was observed across all regions, with the most pronounced decline occurring in high SDI regions (EAPC = -0.74; 95% CI: -2.11 to 0.65). This suggests that while the absolute number of new leukemia cases is increasing globally, the incidence rate per standardized age group is declining, particularly in more socioeconomically developed regions. Among the 21 geographic regions, Western Europe (755, 73) had the most cases in 2021. There was a decreasing trend in leukemia incidence across all regions, most notably in Oceania (EAPC =-1.75, 95% CI: -4.35 to 0.92), followed by Central Asia and High-income North America, with EAPCs of -1.05 (95% CI: -2.26 to 0.18) and -1.04 (95% CI: -2.7 to 0.64), see S1 Table.

Among the 204 countries, new cases of leukemia in 2021 ranged from 9,887 in the United Kingdom to 0 in Palau .143 countries showed a decreasing trend in leukemia ASIR, with Guam, Cook Islands, and Ghana showing the largest decreases, with EAPCs of -2.79 (95% CI: -5.31 to -0.21), -2.53 (95% CI: -4.82 to -0.19) and -2.32 (95% CI: -4.03 to -0.58) respectively. In contrast, 59 countries showed an upward trend, especially Lesotho, Egypt and Zimbabwe, with EAPCs of 2.4 (95% CI: -0.14 to 5), 1.97 (95% CI: 0.69 to 3.28) and 1.78 (95% CI: -0.87 to 4.49), respectively. Chile and Uganda There was no change in two countries, see S2 Table.

In 2021, the global number of leukemia deaths was reported at 320,284 (95% uncertainty interval (UI): 274,969 to 349,050), reflecting a substantial increase of 29.09% compared to 1990. Despite this rise in the absolute number of deaths, the age-standardized mortality rate (ASMR) exhibited a downward trend, with an average annual decrease of 1.04% from 1990 to 2021 (EAPC = -1.25; 95% CI = -2.02 to -0.48). See Table 1 and Fig 1.

Moreover, it was observed that men had a higher number of leukemia-related deaths compared to women, although the declining trend in ASMR was less pronounced among men. The decreasing trends in ASMR were consistent across all regions stratified by the SDI as well as geographic areas. Out of the 204 countries assessed, 153 countries recorded a decline in ASMR for leukemia, with Guam showing the most significant decrease (EAPC = -2.91; 95% CI = -4.54 to -1.26), followed by the Cook Islands and the Republic of Korea. Conversely, 40 countries experienced an increase in ASMR, with Lesotho exhibiting the most pronounced rise (EAPC = 1.75; 95% CI = 0.1 to 3.43). This variation underscores the importance of localized health interventions and the differing trends in leukemia mortality across diverse regions.

**Fig 2:**
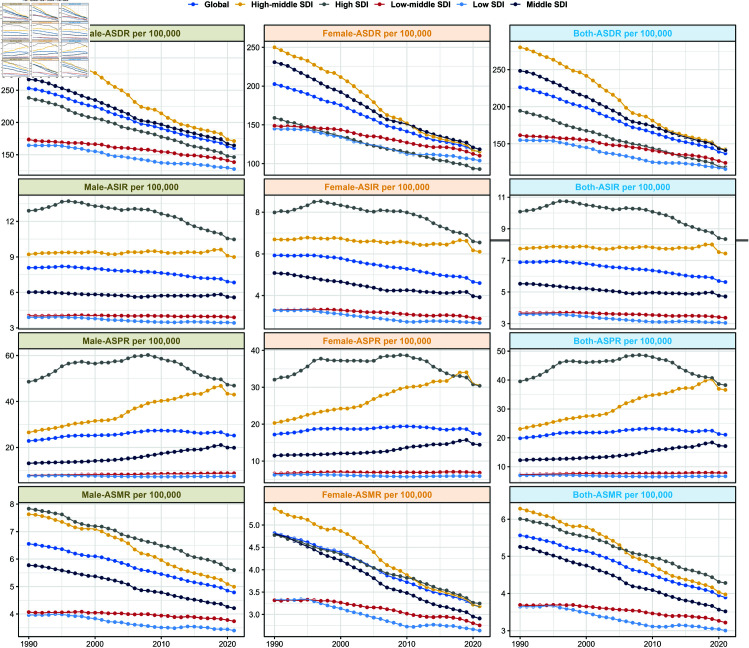
Global and five SDI regions’ leukemia burden trends, 1990-2021.

The global ASDR for leukemia decreased from 11,761,683 cases (95% UI: 10,032,321 to 13,623,198) in 1990, to 10,982,836 cases (95% UI: 9,018,077 to 12,286,933) in 2021. Overall ASDR showed a decreasing trend with an average annual decrease of 1.82% (EAPC = - 1.82; 95% CI: -3.23 to -0.39) . Meanwhile, analysis based on SDI showed a decreasing trend in all geographic regions, especially in the High-middle SDI region (EAPC = -2.34; 95% CI: -3.9 to 0.94). See Table 1 and Fig 2 .

Among the 21 geographic regions, there was a decreasing trend in leukemia ASDR in all regions, most notably in East Asia (EAPC = -2.76, 95% CI: -4.5 to -0.98), followed by High-income Asia Pacific and Eastern Europe, with EAPC of -2.55 (95% CI: -4.54 to -0.53 ) and -2.08 (95% CI: -3.32 to -0.82).

Among the 204 countries, the ASDR for leukemia in 2021 ranged from 3,924,500 in China to 3 in Tokelau. 175 countries showed a decreasing trend in ASDR for leukemia, with Cook Islands, Republic of Korea, and Maldives showing the largest decreases, with EAPC respectively were -3.5 (95% CI: -6.15 to -0.76), -3.46 (95% CI: -5.46 to -1.42) and -3.36 (95% CI: -5.34 to -1.33). In contrast, 28 countries showed an increasing trend, especially Lesotho, Zimbabwe and Chad, with EAPC of 2.53 (95% CI: -0.09 to 5.22), 2.21 (95% CI: -0.49 to 4.98) and 1.53 (95% CI: -0.31 to 3.4), see S2 Table.

**Fig 3:**
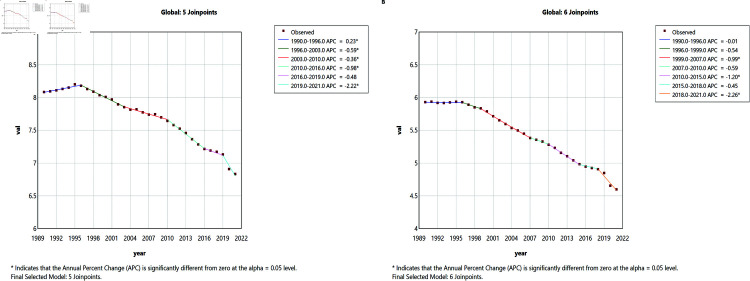
Joinpoint regression analysis of global leukemia incidence in males and females from 1990 to 2021.

Connected-point regression found a significant change in global leukemia incidence in males in 1996, 2003, 2010, 2016, and 2021, AAPC = - 0.5515. The significant change in global leukemia incidence in females in 2007, 2015, and 2021 (Fig 3), AAPC = - 0.8234.There is an overall decreasing trend in global leukemia incidence for both males and females, with a more pronounced decreasing trend in females.

### Burden of the four subtypes of leukemia by gender and age group

**Fig 4:**
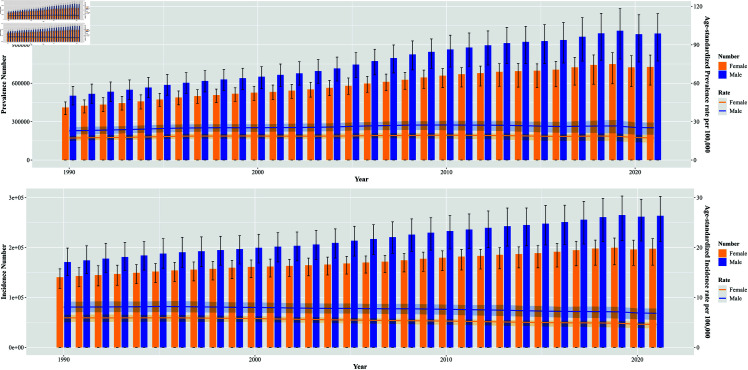
Global Distribution of the Number of Males and Females with Leukemia.

**Fig 5:**
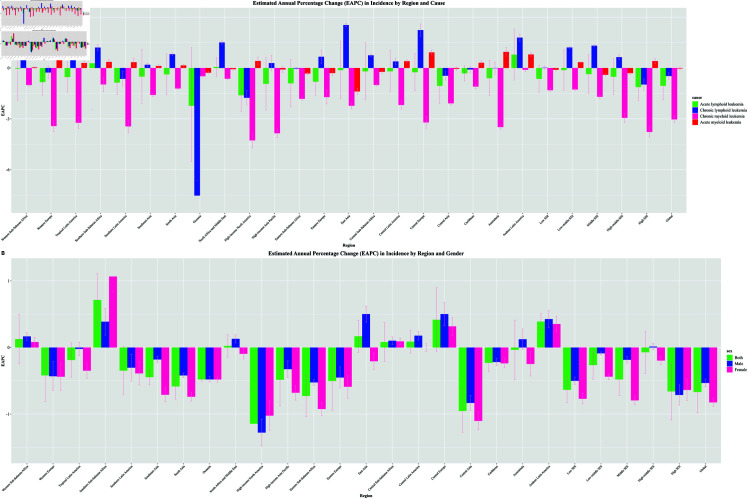
Map of the overall global leukemia burden status, 1990-2021.

Across the SDI regions, there was an increasing trend in the incidence of leukemia in males compared with females, as well as more leukemia deaths in males than in females. The performance of the different subtype species of leukemia globally varied across SDI regions. The greatest decline in CLL was observed in Oceania and the greatest increase in CLL was observed in East Asia. Other different leukemia subtypes showed similar performance in different SDI regions. See Fig 4 and Fig 5

**Fig 6:**
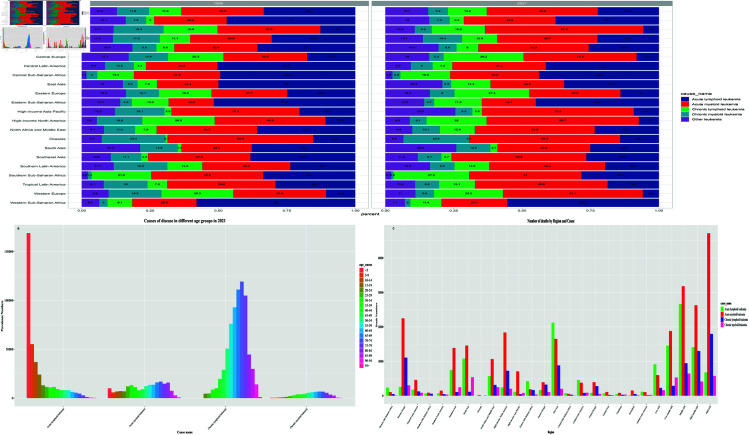
Map of the overall global burden of leukemia, 1990-2021.

In terms of cause of death, 40.6% of global leukemia subtypes in 2021 died from AML and 7.2% from CML, which represent the subtypes with the highest and lowest leukemia fatality rates, respectively. In addition, 22.2% and 14.2% of deaths were due to ALL and CLL (Fig 6).

To study the age pattern of leukemia, we divided the patients into 20 groups of 5 years each according to their age. In 2021, the highest percentage of global leukemia incidence was found in the 0-5 years group (11.31%), followed by the 70-74 years group (10.59%). More than half (59.20%) of the deaths from leukemia occurred in patients over 60 years of age, with the highest number of deaths occurring in the 70-74 and 75-79 age groups, indicating that leukemia is a major health threat to the elderly. The incidence of leukemia was dominated by ALL in the 0-59 year old group and CLL in the 60-79 year old group.2021 The number of incidence, deaths and DALYs of ALL generally peaked in the <5 year old age group, followed by a sharp downward trend, suggesting that childhood is the highest incidence stage for ALL. The age distribution patterns of the remaining three subtypes were similar, with some degree of variation in the distribution of different age groups in different subtypes (Fig 6).

### Correlation between HDI and EAPC

**Fig 7:**
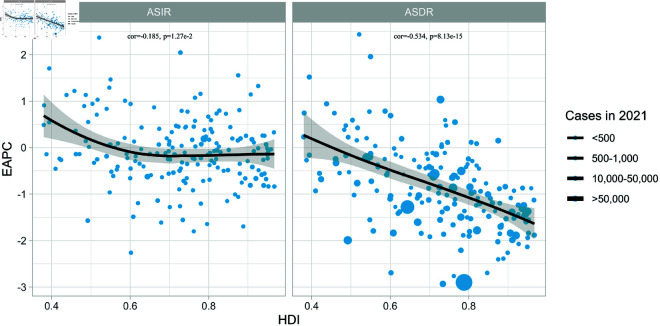
Correlation between HDI and EAPC.

In 2021, leukemia ASIR and ASDR trends were negatively correlated with national HDI (ASIR cor = -0.185, P < 0.005; ASDR cor = -0.534, P < 0.005, see Fig 7), and decreasing trends in leukemia ASIR and ASDR generally occurred in countries with high HDI.

### Frontier analysis based on age-standardized DALYs

**Fig 8:**
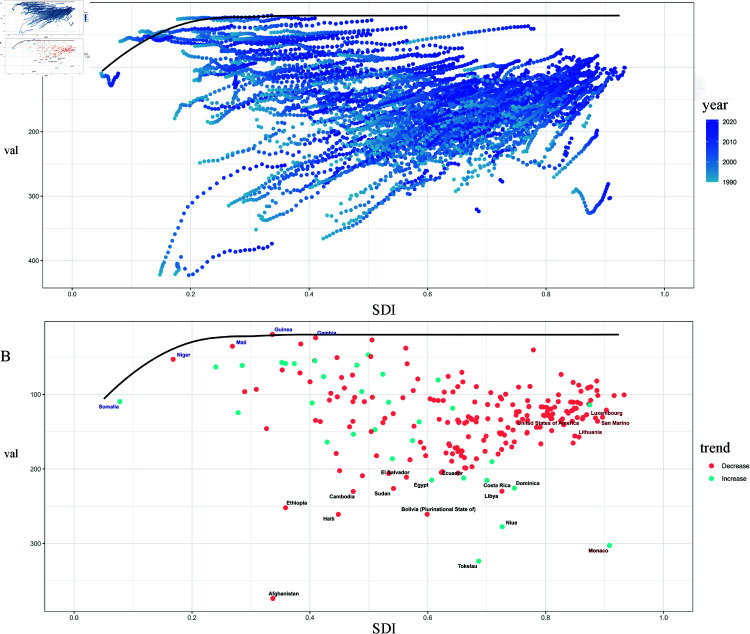
Frontier analysis based on SDI and age-standardized leukemia ASDR per 100,000 population from 1990 to 2021.

To assess the performance of DALYs given a country’s or region’s development status, we conducted a frontier analysis based on age-standardized DALYs and SDI using data from 1990 to 2021. The frontier line delineates the possible age-standardized DALYs based on the SDI. Countries with higher SDI have the lowest effective variance; however, the highest effective variance lies in the middle of the SDI spectrum. The United States of America, Lithuania, Luxembourg, and San Marino are the four countries with the most excellent chance of closing the gap. It is worth noting that the leading performers are not confined to developed countries - two countries with low SDI (<0.2), Somalia, Niger, and Mali, have very little practical difference. In contrast, several countries with high SDI (>0.7), including Niue, Libya, Costa Rica, and Dominica, had disappointing SDI performance (Fig 8).

### Future projections of the global leukemia burden

The global burden of leukemia is expected to continue its downward trend for both males and females from 2021 to 2050. Specifically, the ASPR for leukemia in females is projected to decline from approximately 17.3 per 100,000 population in 2021 to about 8.8 per 100,000 population by 2050. Concurrently, the ASIR for females is anticipated to decrease from roughly 4.6 per 100,000 population in 2021 to approximately 2.3 per 100,000 population in 2050. Additionally, the ASDR)for females is expected to reduce significantly from about 115.2 per 100,000 population in 2021 to approximately 50.1 per 100,000 population in 2050.

Similarly, for males, the ASPR for global leukemia is projected to decrease from approximately 25.2 per 100,000 in 2021 to around 17 per 100,000 by 2050. The ASIR for males is expected to diminish from about 6.8 per 100,000 in 2021 to approximately 4.2 per 100,000 in 2050. The ASDR for males is also projected to decline sharply, from around 160.5 per 100,000 in 2021 to about 83 per 100,000 by 2050.

**Fig 9:**
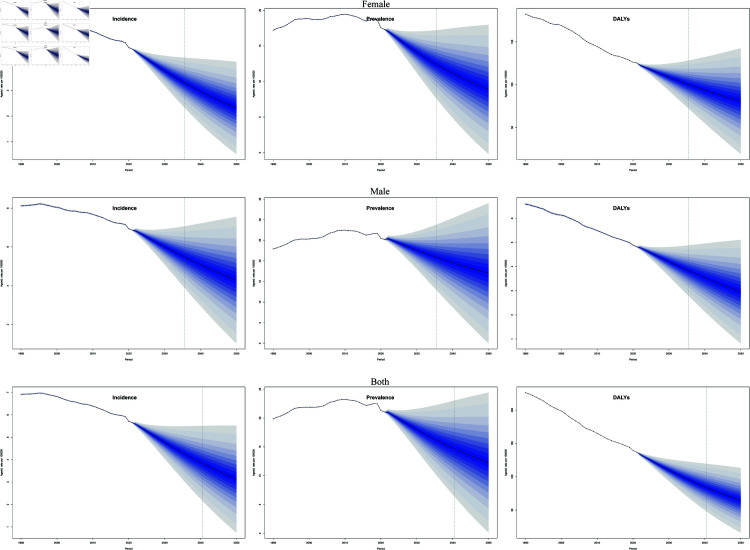
Projected global leukemia burden, 1990-2050.

Overall, these trends signify a hopeful outlook regarding the burden of leukemia, reflecting potential improvements in prevention, early detection, and treatment strategies over the coming decades (Fig 9).

**Fig 10:**
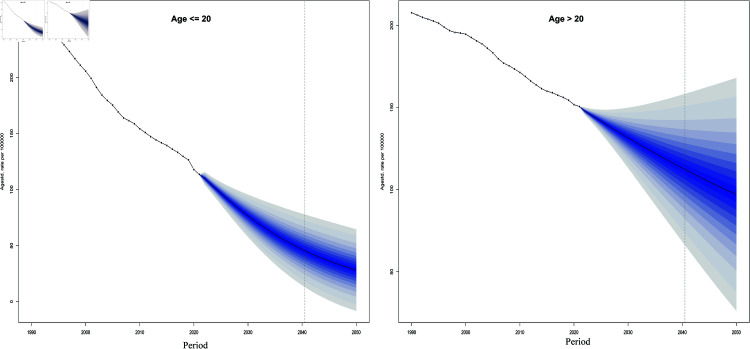
Project the global leukemia burden in patients younger and older than 20 years.

To further investigate the burden of leukemia by age subgroups, we divided age into two groups, less than or equal to 20 years and greater than 20 years. Through our analysis, we found that the leukemic disease burden in both groups showed a decreasing trend, with the decreasing trend being more pronounced in those younger than or equal to 20 years of age (Fig 10).

Globally, the ASDR estimates for leukemia reveal notable disparities when analyzed by region and SDI from 1990 to 2021. Specifically, regions such as High-Income Asia Pacific, Central Latin America, Western Europe, and North Africa and the Middle East reported ASDR estimates that were higher than what would be expected based on their SDI levels during this period.

Conversely, certain areas, including Western Sub-Saharan Africa, Central Sub-Saharan Africa, Southern Sub-Saharan Africa, Central Asia, and Tropical Latin America demonstrated lower than expected ASDR based on their SDI levels across all years evaluated. This indicates a complex relationship between socio-economic development and leukemia mortality, where established correlations do not uniformly apply.

**Fig 11:**
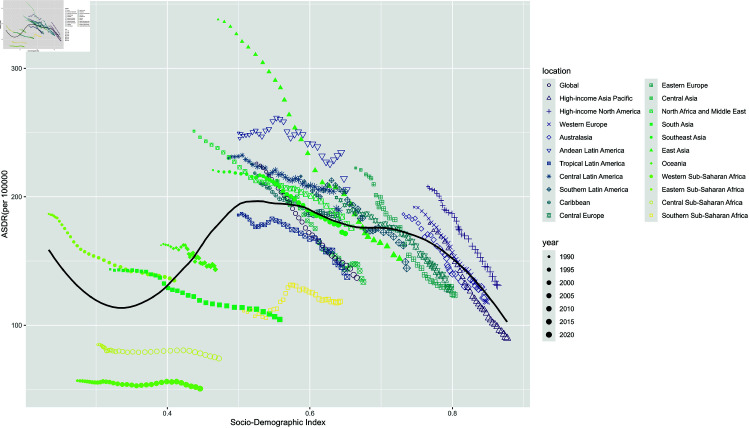
Leukemia ASDR by SDI for 22 GBD regions from 1990 to 2021.

Additionally, the analysis revealed that the highest age-standardized disability-adjusted life year (DALY) rates per 100,000 population were observed in regions categorized with medium SDI levels. This highlights the significant health burden leukemia imposes in these areas, suggesting that factors beyond income, such as access to healthcare and treatment options, may influence both incidence and mortality rates effectively (Fig 11).

**Fig 12:**
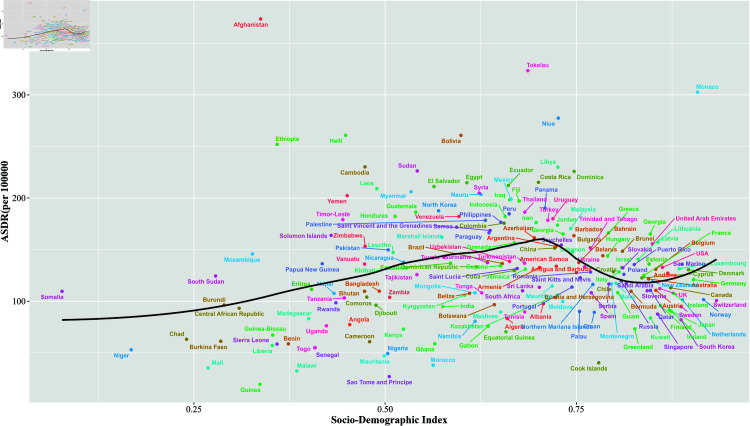
Leukemia ASDR and SDI for 204 countries and territories in 2021.

Country-level analysis reveals that the high burden of leukemia extends beyond developing nations, demonstrating that it is not solely confined to areas with lower socio-economic status. Notably, countries such as Afghanistan, Tokelau, Laos, Bolivia, Haiti, and Yemen exhibit significant leukemia burdens. However, the data indicate that leukemia incidence and mortality rates are surprisingly high in certain regions and countries with high SDI scores. For instance, countries like Monaco, Georgia, the USA, Brunei, and France are typically classified as developed nations show a leukemia burden that exceeds what would be anticipated based on their SDI. This finding underscores the complexity of leukemia prevalence, suggesting that lifestyle, genetic predisposition, environmental influences, and healthcare access play crucial roles in leukemia outcomes across diverse geographic and economic contexts (Fig 12). This highlights the need for tailored public health strategies and research initiatives addressing the specific challenges faced by low and high SDI countries regarding leukemia management and prevention.

## Discussion

Over the past several decades, significant advancements in the treatment of leukemia and improvements in associated prognoses have been achieved. These advancements have resulted in notable changes in survival patterns for patients with leukemia. This study offers a comprehensive overview of the global burden of leukemia and its four major subtypes, informed by the latest findings from the GBD 2021 study.

The GBD 2021 report indicates a decline in age-standardized ratios (ASRs) for both mortality and the DALYs across the four major leukemia subtypes from 1990 to 2021. The EAPC observed during this period suggests a global improvement in leukemia burden, likely attributable to enhancements in living standards worldwide and continuous advancements in medical technology. These improvements encompass better diagnostic methods, more effective treatment protocols, and increased healthcare access, contributing to improved outcomes for patients.

Furthermore, our projections regarding the leukemia burden by 2050 are crucial for understanding future trends in disease dynamics. These insights will aid policymakers in devising strategic interventions to mitigate leukemia’s impacts. By anticipating the potential trajectory of the disease, effective public health policies can be crafted to address prevention, early detection, and treatment options, ultimately reducing the harm inflicted by leukemia on affected populations. As the landscape of leukemia management continues to evolve, ongoing research and proactive planning will be essential in enhancing patient care and outcomes on a global scale.

Demographic factors and socioeconomic status largely influence significant and uneven differences in the global disease burden [21, 22]. The disease burden is most severe in East and South Asia, primarily attributed to large population sizes and inadequate healthcare resources [23, 24].

Low-income and lower-middle-income countries typically have lower incidence rates, usually less than 10 cases per 100,000 population.In 2021, Southern Sub-Saharan Africa had an incidence rate of 3.78 cases per 100,000 population (2.68-4.38 cases), with an EAPC of 0.37 [-2.1 to 2.9], suggesting an increasing burden of leukemia in the region.Western Sub-Saharan Africa had an incidence rate of 1.17 cases per 100,000 population (0.7-1.49 cases), with an EAPC of -0.02 [-1.7 to 1.9]. Western Sub-Saharan Africa had an incidence rate of 1.17 (0.7-1.49) cases per 100,000 population, with an EAPC of -0.02 [-1.7 to 1.69], indicating a decreasing leukemia burden in the region.

Among the individual countries in the South American region, leukemia incidence showed different trends. Ecuador had the fastest increasing trend with an EAPC of 0.85 [-0.62 to 2.33]. Argentina had the fastest decreasing trend with an EAPC of -0.44 [-1.69 to 0.83]. Therefore, the health policy of each country should be formulated in accordance with the epidemiological characteristics of the region, the level of socio-economic development, and the allocation of health care resources for precise intervention.

Our study found that mortality and DALY due to leukemia and its major subtypes showed a significant downward trend in high SDI regions and high-income countries, which may be closely related to adequate healthcare resources and well-established healthcare systems [25, 26]. In high SDI regions, particularly in Western Europe, the ASPR for leukemia is trending upwards. This increase can be attributed to several factors. One significant reason is the improved accessibility to comprehensive population-based leukemia registry data in many high-income countries. These countries benefit from robust healthcare infrastructures that facilitate accurate diagnosis and reporting of leukemia cases. In contrast, low-income countries often encounter challenges such as under-diagnosis and incomplete disease registration, which can obscure the true burden of leukemia in those regions. However, it is important for developing countries or regions to have better health systems to diagnose leukemia and to have access to available information [27].

In addition, population aging may also contribute to this outcome, as aging is more likely to occur in developed countries [28]. Investigating the trends among the four main subtypes of leukemia, significant differences in ASIR patterns have been identified across different CML and ALL SDI quintiles. These findings highlight the need for targeted research and tailored public health strategies considering the unique challenges and risk factors associated with leukemia subtypes across varying socio-economic contexts [29]. Understanding these patterns is essential for effective planning and resource allocation to combat leukemia globally.

Based on the prevalence of leukemia per million population in 2021, ALL is the main causative factor in infants and young children, while CLL mainly affects the elderly. Age-related mortality data show that leukemia most often causes death in older patients and young people in poor health. Previous studies have shown that ALL is the most common type of leukemia in children and is slightly more prevalent in boys than in girls [30, 31].

Following childhood and adolescence, the occurrence of ALL reaches its highest point in older adults, alongside trends in the other three leukemia subtypes. The role of aging in the distribution of leukemia is a significant factor that warrants attention, as highlighted in previous research [32]. Recent investigations have also shed light on the mechanisms linking aging to leukemia [33].

As individuals age, hematopoietic stem cells (HSCs) gradually diminish in their ability to regenerate, resulting in typical signs of blood aging such as immune senescence, anemia, and imbalances in myeloid cell production. These age-related changes contribute to a heightened risk of developing autoimmune disorders and hematologic cancers [34]. The correlation between aging and leukemia incidence clarifies why the highest rates of this disease are found among the elderly in regions with a high SDI, where an aging population plays a crucial role. Older patients often face a worse prognosis compared to their younger counterparts, attributed to factors such as suboptimal physical health at diagnosis, lower rates of complete remission following intensive chemotherapy, higher early mortality associated with treatment, resistance to chemotherapy, and an increased frequency of adverse cytogenetic outcomes and secondary AML [35].

The disparity in leukemia prevalence and incidence between genders may be attributed to various risk factors, including smoking and obesity. Smoking, established as a significant risk factor for leukemia for over ten years, plays a considerable role in this difference [36]. Countries with low HDI often experience a greater burden of leukemia and tend to have worse prognoses [37].

In regions with low to medium SDI, including much of Latin America and East Asia, a slight decrease in leukemia trends has been noted. This may signify the positive impacts of improved local health infrastructure, international collaboration, and health assistance [38]. For instance, Brazil’s primary healthcare system is crucial in delivering effective interventions for AML [39] In China, there has been a marked decline in mortality rates and the DALYs, largely attributed to advancements in socio-economic conditions and medical resources. The new rural cooperative medical system in China now covers around 80% of the rural population (approximately 830 million people), significantly enhancing the accessibility and affordability of healthcare services [40, 41].

The GBD 2021 study provided essential and current data that supported the successful execution of this research. Nonetheless, this study has certain limitations. First, issues such as underreporting and misdiagnosis can introduce bias regarding the true incidence cases, prevalence rates, and corresponding age-standardized rates, particularly due to diagnostic inaccuracies in some less developed countries. Second, despite previous studies illustrating the influence of racial and ethnic differences on leukemia, relevant data for subgroup analysis was unavailable from the GBD database [42]. Lastly, some rare forms of leukemia possess complex diagnostic criteria and are categorized simply as "other leukemia," resulting in the exclusion of these subtypes from our analysis.

## Conclusion

This study indicates that while the incidence, mortality, and the DALYs associated with leukemia have been rising over the years, the ASIR, ASMR, and ASDR have demonstrated a declining trend. This suggests that the overall burden of leukemia has decreased over the past 50 years. The increasing burden of leukemia is notably concentrated in countries and regions with a higher SDI, highlighting the need for greater investment in relevant research to mitigate the incidence and prevalence of leukemia in developing areas. Additionally, findings reveal that leukemia incidence is higher among men and older age groups, emphasizing the importance for policymakers to consider these demographic differences when shaping prevention strategies. The prevalence of leukemia also exhibited a bimodal distribution, particularly increasing in the 1-4 and 90-94 age groups, which underscores the necessity for targeted leukemia screening in these specific age brackets. At the same time, “other types of leukemia” must receive more attention in future research and public health initiatives. The burden of leukemia is expected to increase further in the future as populations age in many developed countries. Our projections for future trends up to 2050 suggest that the ASIR and ASDR will continue to rise for several leukemia subtypes. To effectively address this anticipated increase in leukemia burden, policymakers must focus on enhancing healthcare systems, facilitating early diagnosis and treatment, and promoting health education initiatives to reduce the global burden of leukemia. By investing in these areas, there is potential to mitigate the impacts of leukemia and improve outcomes for affected populations.

## Supporting information

S1 Table Regions of ASDR, ASPR and ASMR (DOCX)

S2 Table Countries of ASIR, ASPR and ASDR (DOCX)
