# Supplementary material for: Global, regional, and national burden of leukemia: Epidemiological trends analysis from 1990 to 2021
Source: PLoS One. 2025 Jun 26;20(6):e0325937. doi: 10.1371/journal.pone.0325937 (PMC12200851; doi:10.1371/journal.pone.0325937)
Supplement: S1 Table — (DOCX) [file pone.0325937.s001.docx]

|  | **1990** | | **2021** | | **1990-2021** |
| --- | --- | --- | --- | --- | --- |
|  | **Prevalence cases** | **ASPR per 100,000** | **Prevalence cases** | **ASPR per 100,000** | **EAPC** |
|  | **No. *10^2^ (95% UI)** | **No. (95% UI)** | **No. *10^2^ (95% UI)** | **No.(95% UI)** | **No. (95% CI)** |
| **Overall** | 9136.08 [8359.91-9953.97] | 19.83 [18.24-21.43] | 17159.11 [14491.15-19095.82] | 21.07 [17.65-23.61] | -0.09 [-1.55 to 1.4] |
| Sex |  | | | | |
| Female | 4110.96 [3556.4-4542.09] | 17.17 [14.92-18.86] | 7272.16 [5853.68-8194.98] | 17.32 [13.74-19.48] | -0.21 [-1.68 to 1.29] |
| Male | 5025.12 [4300.86-5748.45] | 22.86 [19.93-25.72] | 9886.95 [7735.01-11434.62] | 25.21 [19.78-29.3] | 0 [-1.47 to 1.49] |
| **Socio-demographic index** |  | | | | |
| High SDI | 3854.37 [3718.91-3946.13] | 39.52 [38.22-40.57] | 5958.67 [5517.61-6287.21] | 38.23 [35.47-40.49] | -0.2 [-1.76 to 1.38] |
| High-middle SDI | 2352.9 [2113.27-2548.09] | 23.08 [20.74-25.05] | 5095.98 [3997.05-5857.35] | 36.58 [27.33-44.53] | 0.89 [-0.83 to 2.64] |
| Middle SDI | 1899.66 [1546.13-2227.47] | 12.28 [9.92-14.41] | 4188.02 [3115.86-4898.49] | 17.11 [12.72-20.26] | 0.99 [-0.67 to 2.68] |
| Low-middle SDI | 728.42 [552.29-948.46] | 7.29 [5.81-9.35] | 1334.77 [1086.22-1628.21] | 7.79 [6.41-9.54] | 0.37 [-1.12 to 1.88] |
| Low SDI | 292.56 [191.42-398.5] | 7.01 [5.05-8.96] | 565.42 [385.35-723.37] | 6.68 [4.6-8.71] | -0.29 [-1.79 to 1.22] |
| **Region** |  | | | | |
| Andean Latin America | 37.58 [31.95-45.91] | 11.47 [9.79-13.93] | 101.63 [73.05-128.87] | 16.06 [11.55-20.36] | 1.56 [-0.15 to 3.3] |
| Australasia | 88.32 [83.03-93.33] | 39.77 [37.26-42.17] | 193.07 [173.34-213.93] | 43.37 [39.42-48.02] | 0.16 [-1.51 to 1.84] |
| Caribbean | 40.85 [36.34-46.89] | 12.94 [11.68-14.59] | 69.21 [59.48-79.45] | 13.79 [11.77-15.9] | 0.22 [-0.99 to 1.45] |
| Central Asia | 80.2 [75.31-84.97] | 12.76 [12.01-13.5] | 101.42 [88.26-115.54] | 10.8 [9.44-12.27] | -0.54 [-2.1 to 1.03] |
| Central Europe | 249.25 [238.53-262.47] | 17.57 [16.82-18.54] | 590.39 [530.13-644.11] | 30.47 [27.35-33.41] | 0.91 [-0.83 to 2.68] |
| Central Latin America | 142.13 [137.3-147.9] | 9.84 [9.54-10.13] | 318.82 [283.97-367.2] | 12.98 [11.51-15.05] | 0.61 [-0.83 to 2.07] |
| Central Sub-Saharan Africa | 13.27 [9.75-17.89] | 3.14 [2.27-4] | 34.54 [21.81-48.32] | 3.8 [2.2-5.56] | 0.45 [-1.45 to 2.39] |
| East Asia | 2017.07 [1540.41-2409.13] | 17.74 [13.64-21.28] | 5404.23 [3605.44-6745.85] | 39.28 [25.84-51.42] | 2.04 [-0.08 to 4.21] |
| Eastern Europe | 518.55 [490.3-544.27] | 20.53 [19.38-21.58] | 746.15 [681.64-809.79] | 25.34 [23.16-27.46] | 0.19 [-1.43 to 1.83] |
| Eastern Sub-Saharan Africa | 138.4 [87.35-197.08] | 9.05 [6.46-11.79] | 269.45 [175.76-381.47] | 8.84 [6-12.54] | -0.44 [-2.31 to 1.47] |
| High-income Asia Pacific | 329.05 [308.68-356.11] | 20.63 [18.91-23.09] | 546.32 [489.77-588.17] | 24.95 [22.14-27.57] | 0.11 [-1.45 to 1.71] |
| High-income North America | 1775.03 [1703.04-1818.63] | 55.6 [53.63-56.95] | 2190.4 [2037-2288.92] | 40.41 [38.22-42.16] | -0.77 [-2.53 to 1.02] |
| North Africa and Middle East | 340.59 [263.27-425.28] | 12.1 [9.31-14.81] | 926.94 [636.82-1094.28] | 17.35 [11.87-20.43] | 1.21 [0.26 to 2.17] |
| Oceania | 3.15 [1.72-4.37] | 5.98 [3.08-8.77] | 6.73 [4.07-9.2] | 5.54 [3.37-7.82] | -1.57 [-3.89 to 0.81] |
| South Asia | 650.24 [480.57-827.38] | 7.03 [5.27-8.82] | 1145.96 [915.69-1410.98] | 6.87 [5.48-8.49] | 0.13 [-1.16 to 1.43] |
| Southeast Asia | 363.61 [286.08-497.54] | 9.26 [7.44-12.42] | 681.27 [569.74-908.13] | 9.97 [8.37-13.25] | 0.18 [-1.29 to 1.67] |
| Southern Latin America | 61.26 [58.22-64.39] | 12.71 [12.07-13.36] | 113.85 [103.66-124.19] | 15.51 [14.13-17.09] | 0.34 [-0.95 to 1.65] |
| Southern Sub-Saharan Africa | 22.15 [16.42-26.03] | 5.88 [4.02-7.18] | 55.6 [36.28-66.29] | 8.29 [5.28-9.83] | 0.68 [-1.95 to 3.38] |
| Tropical Latin America | 104.77 [100.72-109.07] | 8.26 [7.93-8.56] | 247.42 [233.16-261.54] | 10.31 [9.65-10.98] | 0.36 [-0.91 to 1.64] |
| Western Europe | 2122.63 [2042.14-2185.57] | 46.97 [45.17-48.55] | 3314.97 [3037.15-3544.27] | 49.4 [46.01-52.74] | 0.01 [-1.61 to 1.66] |
| Western Sub-Saharan Africa | 37.96 [27.02-47.1] | 2.16 [1.46-2.65] | 100.75 [53.61-134.79] | 2.55 [1.39-3.31] | 0.38 [-1.64 to 2.45] |

|  | **1990** | | **2021** | | **1990-2021** |
| --- | --- | --- | --- | --- | --- |
|  | **DALYs cases** | **ASDR per 100,000** | **DALYs cases** | **ASDR per 100,000** | **EAPC** |
|  | **No. *10^2^ (95% UI)** | **No. (95% UI)** | **No. *10^2^ (95% UI)** | **No.(95% UI)** | **No. (95% CI)** |
| **Overall** | 117616.83 [100323.21-136231.98] | 226.31 [195.52-258.9] | 109828.36 [90180.77-122869.33] | 136.94 [111.89-153.71] | -1.82 [-3.23 to -0.39] |
| **Sex** |  | | | | |
| Female | 52981.49 [40625.86-62793.29] | 202.53 [156.01-237.17] | 46919.85 [37765.17-51744.59] | 115.29 [92.39-127.61] | -2 [-3.47 to -0.51] |
| Male | 64635.34 [48605.57-80974.3] | 253 [197.99-309.56] | 62908.51 [47736.18-74596.76] | 160.41 [121.78-190.31] | -1.7 [-3.06 to -0.32] |
| **Socio-demographic index** |  | | | | |
| High SDI | 18692.2 [18106.81-19151.34] | 194.46 [188.27-199.21] | 19243.18 [17917.76-20174.67] | 118.04 [110.5-123.67] | -2.02 [-3.44 to -0.58] |
| High-middle SDI | 28734.52 [24163.07-32041.15] | 280.28 [235.26-313.46] | 21030.9 [17207.78-23602.98] | 142.51 [114.31-161.9] | -2.43 [-3.9 to -0.94] |
| Middle SDI | 42831.71 [34583.37-51083.06] | 248.4 [203.37-292.96] | 34867.8 [28034.57-39801.61] | 141.23 [112.8-160.61] | -1.8 [-3.37 to -0.2] |
| Low-middle SDI | 19064.14 [14359.98-24609.44] | 161.36 [127.36-202.58] | 22507.23 [18425.58-26667.16] | 124.05 [102.27-147.8] | -0.75 [-2.4 to 0.94] |
| Low SDI | 8190.04 [5184.68-11738.48] | 154.95 [110.09-206.82] | 12071.2 [8352.66-15121.13] | 115.67 [82.03-143.96] | -1.04 [-2.4 to 0.34] |
| **Region** |  | | | | |
| Andean Latin America | 987.01 [832.44-1248.94] | 249.76 [213.22-312.26] | 1326.24 [974.09-1634.11] | 205.53 [151.29-253.23] | -0.51 [-2.4 to 1.41] |
| Australasia | 403.82 [390.25-416.49] | 186.49 [180.25-192.43] | 548.82 [505.64-588.02] | 121.37 [113.47-129.46] | -2.04 [-3.37 to -0.69] |
| Caribbean | 785.99 [635.32-960.42] | 225.46 [186.33-271.37] | 848.58 [679.59-1062.38] | 179.59 [141.19-228.8] | -0.74 [-2.15 to 0.69] |
| Central Asia | 1502.59 [1414.5-1596.93] | 212.87 [201.01-224.79] | 1264.38 [1100.58-1442.17] | 133.7 [116.69-152.05] | -1.75 [-3.32 to -0.15] |
| Central Europe | 2641.33 [2537.9-2749.51] | 199.34 [191.31-208.11] | 2240.03 [2032.32-2426.27] | 127.94 [116.02-138.74] | -1.9 [-3.19 to -0.6] |
| Central Latin America | 3962.11 [3823.06-4132.23] | 230.01 [223.17-237.69] | 4772.9 [4277.57-5374.38] | 189.53 [169.34-214.14] | -1.02 [-2.87 to 0.87] |
| Central Sub-Saharan Africa | 473.06 [316.4-696.66] | 84.79 [63.33-112.04] | 875.82 [574.65-1169.92] | 74.11 [47.19-104.56] | -0.19 [-2.24 to 1.9] |
| East Asia | 39967.83 [30280.63-48118.79] | 337.98 [257.17-407.77] | 22895.47 [16873.73-28345.55] | 151.75 [110.03-184.35] | -2.76 [-4.5 to -0.98] |
| Eastern Europe | 5155.47 [5043.75-5277.4] | 222.39 [216.95-228.54] | 3345.96 [3090.92-3623.57] | 123.51 [114.58-133.02] | -2.08 [-3.32 to -0.82] |
| Eastern Sub-Saharan Africa | 3738.98 [2345.73-5401.93] | 186.64 [130.04-250.23] | 5278.91 [3557.74-7185.35] | 134.81 [92.01-182.31] | -1.19 [-2.68 to 0.32] |
| High-income Asia Pacific | 3193.59 [2978.49-3355.94] | 179.13 [165.8-189.31] | 2661.56 [2405.05-2857.55] | 89.87 [81.14-95.87] | -2.55 [-4.54 to -0.53] |
| High-income North America | 6547.16 [6339.92-6695.62] | 208.03 [202.4-212.24] | 7130.22 [6664.41-7406.09] | 130.8 [124.1-135.24] | -1.85 [-3.42 to -0.24] |
| North Africa and Middle East | 8184.13 [6208.98-10312.45] | 251.1 [194.27-307.43] | 10014.59 [7237.57-11910.05] | 175.9 [128.52-208.03] | -0.91 [-1.93 to 0.13] |
| Oceania | 100.05 [58.66-136.59] | 162.36 [92.87-218.1] | 191.88 [118.02-258.03] | 143.43 [87.22-193.7] | -1.85 [-4.82 to 1.21] |
| South Asia | 15646.6 [11424.14-20037.09] | 143.51 [111.37-176.87] | 18192.71 [14824.42-22317.16] | 104.45 [85.06-128.32] | -0.98 [-2.39 to 0.44] |
| Southeast Asia | 9927.31 [7645.44-12809.99] | 220.71 [174.03-278.49] | 11728.9 [9314.79-13816.59] | 171.34 [136.04-201.81] | -1.05 [-2.95 to 0.89] |
| Southern Latin America | 1059.14 [1028.24-1091.31] | 216.02 [209.68-222.52] | 1060.14 [1002.02-1132.61] | 144.44 [136.03-155.48] | -1.51 [-2.96 to -0.05] |
| Southern Sub-Saharan Africa | 501.67 [393.69-596.99] | 111.01 [84.62-131.3] | 857.24 [637.77-1017.08] | 118.51 [87.2-139.57] | 0.05 [-2.45 to 2.61] |
| Tropical Latin America | 2677.76 [2551.8-2813.87] | 185.77 [178.84-193.81] | 3217.87 [3063.58-3349.23] | 137.56 [130.17-144.29] | -1.11 [-2.7 to 0.51] |
| Western Europe | 8800.71 [8558.4-9017.02] | 191.91 [187.84-196.11] | 8640.51 [8013.23-9110.39] | 118.39 [111.96-124.1] | -2.07 [-3.49 to -0.63] |
| Western Sub-Saharan Africa | 1360.53 [967.61-1796.21] | 56.87 [41.11-71.95] | 2735.64 [1473.99-3713.88] | 50.6 [28.91-66.8] | -0.27 [-2.14 to 1.64] |

|  | **1990** | | **2021** | | **1990-2021** |
| --- | --- | --- | --- | --- | --- |
|  | **Deaths cases** | **ASMR per 100,000** | **Deaths cases** | **ASMR per 100,000** | **EAPC** |
|  | **No. *10^2^ (95% UI)** | **No. (95% UI)** | **No. *10^2^ (95% UI)** | **No.(95% UI)** | **No. (95% CI)** |
| **Overall** | 2481.05 [2177.86-2773.54] | 5.56 [4.97-6.13] | 3202.84 [2749.69-3490.5] | 3.89 [3.34-4.25] | -1.25 [-2.02 to -0.48] |
| Sex |  | | | | |
| Female | 1132.23 [932.89-1276.48] | 4.82 [4.01-5.36] | 1387.01 [1158.54-1527.83] | 3.17 [2.64-3.49] | -1.45 [-2.25 to -0.65] |
| Male | 1348.83 [1096.94-1607.06] | 6.55 [5.51-7.58] | 1815.82 [1468.02-2129.13] | 4.79 [3.91-5.59] | -1.12 [-1.87 to -0.36] |
| **Socio-demographic index** |  | | | | |
| High SDI | 633.06 [602.54-650.88] | 6.01 [5.73-6.18] | 888.85 [802.52-942.08] | 4.28 [3.94-4.52] | -1.44 [-2.32 to -0.55] |
| High-middle SDI | 625.71 [547.29-679.78] | 6.28 [5.51-6.83] | 689.75 [589.8-765.85] | 3.98 [3.36-4.43] | -1.46 [-2.25 to -0.65] |
| Middle SDI | 750.02 [620.8-874.97] | 5.25 [4.39-6.04] | 888.96 [727.71-1014.77] | 3.52 [2.87-4] | -1.22 [-2.08 to -0.35] |
| Low-middle SDI | 333.16 [264.93-416.34] | 3.69 [3.07-4.48] | 509.59 [427.74-607.47] | 3.22 [2.7-3.87] | -0.33 [-1.22 to 0.56] |
| Low SDI | 136.5 [95.55-184.84] | 3.64 [2.78-4.56] | 222.29 [157.75-276.21] | 3 [2.16-3.76] | -0.62 [-1.35 to 0.1] |
| **Region** |  | | | | |
| Andean Latin America | 17.02 [14.53-21.25] | 5.47 [4.7-6.75] | 30.95 [23.18-37.97] | 5.01 [3.76-6.13] | -0.03 [-1.04 to 0.99] |
| Australasia | 14.01 [13.37-14.56] | 6.17 [5.88-6.4] | 26.24 [23.5-28.46] | 4.81 [4.36-5.19] | -1.31 [-2.15 to -0.48] |
| Caribbean | 16.09 [13.98-18.55] | 5.3 [4.72-5.99] | 22.07 [18.78-25.87] | 4.37 [3.69-5.19] | -0.65 [-1.36 to 0.07] |
| Central Asia | 27.04 [25.64-28.48] | 4.38 [4.17-4.6] | 26.79 [23.57-30.19] | 3.01 [2.66-3.37] | -1.31 [-2.18 to -0.44] |
| Central Europe | 80.08 [77.04-83.48] | 5.74 [5.51-5.99] | 95.39 [86.37-102.84] | 4.5 [4.09-4.86] | -1.11 [-1.89 to -0.33] |
| Central Latin America | 66.15 [64.27-68.22] | 4.87 [4.74-5.01] | 110.84 [99.47-123.09] | 4.43 [3.98-4.93] | -0.65 [-1.57 to 0.28] |
| Central Sub-Saharan Africa | 7.88 [5.88-10.78] | 2.1 [1.48-2.75] | 16.4 [10.5-22.98] | 2.02 [1.23-3] | -0.1 [-1.36 to 1.18] |
| East Asia | 688.33 [532.71-816.05] | 6.37 [4.98-7.55] | 613.02 [456.35-768.75] | 3.43 [2.54-4.26] | -1.96 [-2.97 to -0.94] |
| Eastern Europe | 129.4 [126.84-132.02] | 5.11 [5.01-5.22] | 117.54 [108.11-127.18] | 3.7 [3.41-3.99] | -0.97 [-1.7 to -0.23] |
| Eastern Sub-Saharan Africa | 60.81 [41.63-83.37] | 4.47 [3.37-5.74] | 94.16 [64.27-126.42] | 3.58 [2.52-4.98] | -0.77 [-1.68 to 0.14] |
| High-income Asia Pacific | 82.54 [78.86-87.15] | 4.38 [4.16-4.63] | 127.45 [110.85-138.87] | 2.93 [2.63-3.15] | -1.41 [-2.61 to -0.19] |
| High-income North America | 238.72 [224.42-246.05] | 6.91 [6.53-7.11] | 330.08 [295.25-347.21] | 5.09 [4.62-5.33] | -1.24 [-2.23 to -0.25] |
| North Africa and Middle East | 150.94 [117.18-184.4] | 6.31 [4.95-7.59] | 243.21 [180.8-286.97] | 5.03 [3.76-5.9] | -0.71 [-1.27 to -0.15] |
| Oceania | 1.78 [1.02-2.39] | 4.08 [2.37-5.65] | 3.55 [2.15-4.87] | 3.49 [2.13-4.89] | -1.46 [-3.14 to 0.25] |
| South Asia | 277.96 [217.12-340.52] | 3.37 [2.7-4.03] | 430.19 [352.43-529.79] | 2.76 [2.26-3.42] | -0.48 [-1.27 to 0.31] |
| Southeast Asia | 183.7 [146.1-228.99] | 5.2 [4.22-6.33] | 281.9 [229.14-337.63] | 4.33 [3.56-5.2] | -0.63 [-1.69 to 0.44] |
| Southern Latin America | 26.78 [25.91-27.66] | 5.71 [5.52-5.9] | 34.93 [32.36-37.37] | 4.24 [3.95-4.53] | -0.79 [-1.55 to -0.02] |
| Southern Sub-Saharan Africa | 10.7 [8.08-12.72] | 3.14 [2.24-3.85] | 21.45 [15.66-25.15] | 3.53 [2.55-4.08] | 0.04 [-1.57 to 1.67] |
| Tropical Latin America | 52.96 [51.21-54.8] | 4.56 [4.36-4.7] | 94.55 [88.63-98.84] | 3.89 [3.64-4.07] | -0.64 [-1.47 to 0.2] |
| Western Europe | 327.89 [312.34-338.9] | 6.05 [5.8-6.24] | 439.68 [391.16-469.82] | 4.53 [4.14-4.8] | -1.41 [-2.27 to -0.54] |
| Western Sub-Saharan Africa | 20.27 [14.62-25.9] | 1.18 [0.87-1.42] | 42.45 [24.09-56.09] | 1.14 [0.7-1.44] | -0.07 [-1.17 to 1.05] |

|  | **1990** | | **2021** | | **1990-2021** |
| --- | --- | --- | --- | --- | --- |
|  | **YLDs cases** | **YLDs per 100,000** | **YLDs cases** | **YLDs per 100,000** | **EAPC** |
|  | **No. *10^2^ (95% UI)** | **No. (95% UI)** | **No. *10^2^ (95% UI)** | **No.(95% UI)** | **No. (95% CI)** |
| **Overall** | 1321.7 [955.4-1739.6] | 2.98 [2.16-3.9] | 2290 [1616.85-3011.71] | 2.78 [1.96-3.66] | -0.52 [-1.84 to 0.83] |
| **Sex** |  | | | | |
| Female | 585.53 [412.68-762.08] | 2.5 [1.78-3.26] | 952.44 [663.72-1261.94] | 2.21 [1.53-2.94] | -0.67 [-1.98 to 0.65] |
| Male | 736.17 [528.46-989.81] | 3.56 [2.57-4.67] | 1337.56 [927.77-1788.68] | 3.44 [2.39-4.6] | -0.41 [-1.77 to 0.96] |
| **Socio-demographic index** |  | | | | |
| High SDI | 520.7 [380.32-670.03] | 5.09 [3.74-6.58] | 804.21 [586.24-1044.8] | 4.57 [3.36-6.04] | -0.49 [-1.95 to 0.99] |
| High-middle SDI | 335.84 [241.93-444.89] | 3.33 [2.4-4.4] | 636.96 [440.4-861.17] | 4.15 [2.86-5.77] | 0.22 [-1.27 to 1.73] |
| Middle SDI | 294.41 [201.67-403.6] | 2.02 [1.4-2.74] | 549.45 [369.79-745.84] | 2.2 [1.48-2.98] | 0.2 [-1.19 to 1.61] |
| Low-middle SDI | 120.36 [79.71-170.77] | 1.32 [0.89-1.83] | 209.46 [143.33-280.08] | 1.29 [0.88-1.71] | 0.03 [-1.27 to 1.34] |
| Low SDI | 49.11 [29.52-74.4] | 1.31 [0.85-1.86] | 87.61 [55.86-125.96] | 1.15 [0.73-1.66] | -0.55 [-1.81 to 0.73] |
| **Region** |  | | | | |
| Andean Latin America | 6.12 [4.14-8.32] | 1.97 [1.34-2.65] | 14.45 [9.48-20.77] | 2.32 [1.52-3.33] | 0.89 [-0.6 to 2.41] |
| Australasia | 12.31 [8.93-16.01] | 5.4 [3.94-7.02] | 26.91 [19.45-35.19] | 5.54 [3.99-7.27] | -0.17 [-1.7 to 1.38] |
| Caribbean | 6.51 [4.46-8.63] | 2.13 [1.49-2.8] | 10.62 [7.42-14.23] | 2.09 [1.46-2.81] | -0.06 [-1.11 to 1] |
| Central Asia | 11.81 [8.41-15.63] | 1.95 [1.38-2.57] | 14.05 [9.98-19.31] | 1.56 [1.11-2.12] | -0.79 [-2.13 to 0.56] |
| Central Europe | 40.41 [28.67-52.78] | 2.83 [2-3.7] | 83.18 [60.74-109.48] | 4.04 [2.95-5.34] | 0.26 [-1.3 to 1.84] |
| Central Latin America | 23.63 [16.78-30.97] | 1.74 [1.23-2.27] | 48.83 [33.98-66.37] | 1.97 [1.37-2.68] | 0.11 [-1.17 to 1.4] |
| Central Sub-Saharan Africa | 2.53 [1.57-3.69] | 0.67 [0.44-0.97] | 5.96 [3.32-9.41] | 0.73 [0.38-1.22] | 0.17 [-1.65 to 2.01] |
| East Asia | 294.84 [195.25-409.8] | 2.69 [1.78-3.72] | 620.52 [388.08-877.41] | 4.09 [2.53-5.96] | 0.88 [-0.95 to 2.74] |
| Eastern Europe | 75.3 [54.05-98.12] | 2.92 [2.1-3.82] | 102.32 [73.77-133.63] | 3.29 [2.38-4.31] | -0.21 [-1.62 to 1.22] |
| Eastern Sub-Saharan Africa | 22.71 [13.55-34.63] | 1.68 [1.07-2.39] | 39.9 [24.76-60.32] | 1.48 [0.94-2.26] | -0.75 [-2.39 to 0.9] |
| High-income Asia Pacific | 45.24 [32.28-60.43] | 2.63 [1.87-3.52] | 79.62 [56.46-105.47] | 2.79 [1.98-3.74] | -0.21 [-1.59 to 1.18] |
| High-income North America | 236.76 [173.31-304.47] | 7.09 [5.21-9.14] | 303.58 [223.36-389.46] | 5.14 [3.79-6.63] | -0.96 [-2.62 to 0.73] |
| North Africa and Middle East | 55.41 [36.13-78.53] | 2.21 [1.46-3] | 129.42 [83.79-178.84] | 2.57 [1.65-3.53] | 0.55 [-0.28 to 1.38] |
| Oceania | 0.55 [0.29-0.85] | 1.2 [0.61-1.88] | 1.12 [0.63-1.75] | 1.04 [0.57-1.67] | -1.72 [-4.06 to 0.68] |
| South Asia | 104.54 [69.51-149.19] | 1.25 [0.84-1.71] | 179.41 [121.34-247.56] | 1.12 [0.75-1.55] | -0.22 [-1.38 to 0.95] |
| Southeast Asia | 62.84 [40.22-88.97] | 1.74 [1.15-2.42] | 109.87 [74.28-149.07] | 1.65 [1.12-2.25] | -0.27 [-1.63 to 1.11] |
| Southern Latin America | 10.54 [7.49-13.68] | 2.22 [1.57-2.87] | 17.63 [12.82-23.3] | 2.25 [1.63-2.97] | -0.17 [-1.31 to 0.97] |
| Southern Sub-Saharan Africa | 3.86 [2.55-5.25] | 1.1 [0.69-1.55] | 9.22 [5.8-12.99] | 1.45 [0.9-2.03] | 0.55 [-1.94 to 3.11] |
| Tropical Latin America | 18.38 [13-24.06] | 1.56 [1.09-2.04] | 40.24 [28.82-53] | 1.66 [1.19-2.18] | -0.09 [-1.23 to 1.07] |
| Western Europe | 280.75 [204.65-361.24] | 5.67 [4.14-7.37] | 437.34 [321.48-572.07] | 5.65 [4.17-7.45] | -0.25 [-1.75 to 1.28] |
| Western Sub-Saharan Africa | 6.67 [4.26-9.56] | 0.4 [0.25-0.56] | 15.79 [8.13-24.12] | 0.43 [0.23-0.64] | 0.1 [-1.69 to 1.93] |

|  | **1990** | | **2021** | | **1990-2021** |
| --- | --- | --- | --- | --- | --- |
|  | **YLLs cases** | **YLLs per 100,000** | **YLLs cases** | **YLLs per 100,000** | **EAPC** |
|  | **No. *10^2^ (95% UI)** | **No. (95% UI)** | **No. *10^2^ (95% UI)** | **No.(95% UI)** | **No. (95% CI)** |
| **Overall** | 116295.13 [99163.94-134780.22] | 223.33 [192.8-255.43] | 107538.36 [88626.09-120693.51] | 134.15 [110.15-150.8] | -1.63 [-2.57 to -0.67] |
| **Sex** |  | | | | |
| Female | 52395.96 [40071.04-62177.35] | 200.03 [153.84-234.51] | 45967.4 [37045.26-50849.85] | 113.08 [90.67-125.55] | -1.81 [-2.8 to -0.81] |
| Male | 63899.16 [47919.79-80223.54] | 249.44 [195.06-305.58] | 61570.96 [46685.69-73143.5] | 156.97 [119.06-186.17] | -1.5 [-2.41 to -0.59] |
| **Socio-demographic index** |  | | | | |
| High SDI | 18171.5 [17609.07-18567.36] | 189.37 [183.28-193.77] | 18438.97 [17204.92-19311] | 113.47 [106.28-118.83] | -1.92 [-2.86 to -0.96] |
| High-middle SDI | 28398.68 [23840.45-31710.2] | 276.96 [232.17-309.59] | 20393.95 [16706.14-22881.07] | 138.36 [110.97-156.47] | -2.07 [-3.06 to -1.08] |
| Middle SDI | 42537.3 [34378.09-50734.25] | 246.38 [201.7-290.62] | 34318.35 [27559.4-39065.19] | 139.03 [110.92-157.84] | -1.63 [-2.69 to -0.56] |
| Low-middle SDI | 18943.77 [14262.45-24445.53] | 160.04 [126.33-200.96] | 22297.78 [18212.01-26456.35] | 122.77 [100.96-146.06] | -0.64 [-1.76 to 0.49] |
| Low SDI | 8140.92 [5145.65-11668.21] | 153.64 [108.97-205.36] | 11983.6 [8294.02-15006.05] | 114.52 [81.19-142.29] | -0.91 [-1.82 to 0.01] |
| **Region** |  | | | | |
| Andean Latin America | 980.89 [825.99-1240.63] | 247.79 [211.33-309.82] | 1311.79 [964.77-1617.08] | 203.21 [149.79-250.46] | -0.37 [-1.63 to 0.91] |
| Australasia | 391.51 [379.32-402.76] | 181.09 [175.27-186.34] | 521.91 [482.18-558.05] | 115.83 [108.5-123.25] | -1.8 [-2.68 to -0.91] |
| Caribbean | 779.49 [629.25-954.7] | 223.32 [184.44-269.32] | 837.96 [669.62-1052.83] | 177.5 [139.26-226.38] | -0.76 [-1.7 to 0.19] |
| Central Asia | 1490.78 [1400.61-1582.99] | 210.92 [199.19-222.55] | 1250.33 [1088.79-1424.83] | 132.14 [115.39-150.15] | -1.63 [-2.68 to -0.57] |
| Central Europe | 2600.91 [2497.35-2709.27] | 196.51 [188.61-205.3] | 2156.85 [1957.25-2328.68] | 123.9 [112.5-134.43] | -1.55 [-2.4 to -0.69] |
| Central Latin America | 3938.48 [3799.83-4108.29] | 228.27 [221.51-235.99] | 4724.07 [4235.41-5307.27] | 187.56 [167.62-211.59] | -0.99 [-2.22 to 0.25] |
| Central Sub-Saharan Africa | 470.54 [314.73-693.54] | 84.12 [62.81-111.26] | 869.86 [571.15-1162.62] | 73.38 [46.76-103.57] | -0.22 [-1.6 to 1.17] |
| East Asia | 39673 [30110.6-47738.17] | 335.3 [255.6-404.3] | 22274.94 [16389.47-27488.45] | 147.66 [107.16-179.58] | -2.43 [-3.59 to -1.25] |
| Eastern Europe | 5080.17 [4968.09-5201.54] | 219.47 [214.05-225.27] | 3243.63 [2996.38-3508.44] | 120.23 [111.35-129.31] | -1.66 [-2.51 to -0.8] |
| Eastern Sub-Saharan Africa | 3716.27 [2330.07-5368.95] | 184.96 [128.63-248.41] | 5239.01 [3529.52-7129.25] | 133.33 [90.78-180.06] | -1.01 [-2 to -0.01] |
| High-income Asia Pacific | 3148.35 [2936.92-3314.06] | 176.5 [163.32-186.67] | 2581.94 [2328.65-2770.11] | 87.08 [78.62-92.85] | -2.17 [-3.57 to -0.74] |
| High-income North America | 6310.4 [6118.09-6430.07] | 200.95 [195.58-204.48] | 6826.64 [6366.81-7074.5] | 125.66 [119.13-129.76] | -1.72 [-2.76 to -0.67] |
| North Africa and Middle East | 8128.72 [6168.47-10251.62] | 248.89 [192.51-304.48] | 9885.16 [7157.3-11758.03] | 173.33 [126.86-205.26] | -1.02 [-1.71 to -0.33] |
| Oceania | 99.5 [58.31-135.88] | 161.16 [92.17-216.72] | 190.76 [117.27-256.59] | 142.38 [86.49-192.07] | -1.5 [-3.4 to 0.44] |
| South Asia | 15542.06 [11348.77-19915.11] | 142.26 [110.24-175.05] | 18013.3 [14694.16-22049.3] | 103.33 [84.25-126.98] | -0.77 [-1.72 to 0.19] |
| Southeast Asia | 9864.47 [7597.91-12723.11] | 218.98 [172.74-276.11] | 11619.02 [9228.79-13682.02] | 169.69 [134.62-199.78] | -0.81 [-2.08 to 0.49] |
| Southern Latin America | 1048.6 [1018.16-1081.52] | 213.8 [207.55-220.51] | 1042.52 [984.83-1115.09] | 142.19 [134.07-152.82] | -1.27 [-2.24 to -0.3] |
| Southern Sub-Saharan Africa | 497.81 [390.69-592.5] | 109.91 [83.98-129.76] | 848.02 [631.8-1004.24] | 117.06 [86.23-137.91] | -0.2 [-1.84 to 1.46] |
| Tropical Latin America | 2659.37 [2532.74-2794.07] | 184.21 [177.28-192.11] | 3177.63 [3028.82-3308.05] | 135.91 [128.59-142.55] | -1.07 [-2.12 to 0] |
| Western Europe | 8519.96 [8295.62-8711.32] | 186.24 [182.15-189.91] | 8203.17 [7588.09-8636.05] | 112.74 [106.9-117.83] | -2.06 [-2.98 to -1.12] |
| Western Sub-Saharan Africa | 1353.86 [962.63-1786.45] | 56.47 [40.82-71.4] | 2719.85 [1464.94-3698.35] | 50.17 [28.66-66.17] | -0.21 [-1.46 to 1.05] |
