# Supplementary material for: Global, regional, and national burden of leukemia: Epidemiological trends analysis from 1990 to 2021
Source: PLoS One. 2025 Jun 26;20(6):e0325937. doi: 10.1371/journal.pone.0325937 (PMC12200851; doi:10.1371/journal.pone.0325937)
Supplement: S2 Table — (DOCX) [file pone.0325937.s002.docx]

| **Country** | **1990** | | **2021** | | **1990-2021** |
| --- | --- | --- | --- | --- | --- |
|  | **Incident cases** | **ASIR per 100,000** | **Incident cases** | **ASIR per 100,000** | **EAPC** |
|  | **No. *10^2^ (95% UI)** | **No. (95% UI)** | **No. *10^2^ (95% UI)** | **No.(95% UI)** | **No. (95% CI)** |
| Philippines | 25.88 [21.15-31.32] | 5.17 [4.09-6.05] | 42.94 [35.59-51.05] | 4.36 [3.68-5.24] | -0.68 [-2.31 to 0.97] |
| Uzbekistan | 8.71 [7.9-9.66] | 4.79 [4.37-5.28] | 10.79 [8.87-13.1] | 3.31 [2.73-4] | -1.35 [-2.7 to 0.01] |
| Indonesia | 73.02 [54.67-94.34] | 4.96 [3.78-6.33] | 120.45 [96.63-155.24] | 4.76 [3.86-6.21] | -0.16 [-1.74 to 1.44] |
| Azerbaijan | 3.41 [2.79-4.14] | 5 [4.1-6.04] | 4.12 [2.84-5.96] | 4.03 [2.82-5.72] | -0.86 [-2.23 to 0.52] |
| Thailand | 26.23 [19.96-31.16] | 5.97 [4.4-7.11] | 52.24 [31.49-68.83] | 6.07 [3.73-7.92] | -0.55 [-1.91 to 0.83] |
| Malaysia | 7.63 [5.96-9.01] | 5.55 [4.54-6.58] | 15.15 [12.68-19.34] | 5.07 [4.25-6.56] | -0.18 [-1.79 to 1.45] |
| Viet Nam | 17.28 [13.53-22.27] | 3.29 [2.59-4.21] | 28.63 [21.57-38.54] | 2.91 [2.19-3.9] | -0.44 [-2.77 to 1.95] |
| Samoa | 0.06 [0.05-0.08] | 5.53 [4.31-7.25] | 0.08 [0.06-0.11] | 5.16 [3.89-7.03] | -1.63 [-4.1 to 0.91] |
| China | 762.04 [583.12-909.58] | 7.14 [5.52-8.58] | 1056.67 [752.76-1322.37] | 7.21 [4.93-9.05] | -0.23 [-1.81 to 1.38] |
| Albania | 1.1 [0.9-1.4] | 4.26 [3.45-5.56] | 1.55 [1.09-2.4] | 4.32 [3.04-6.35] | 0.27 [-1.19 to 1.75] |
| Tonga | 0.02 [0.02-0.03] | 3.04 [2.18-4.07] | 0.03 [0.02-0.04] | 3.01 [2.15-4.03] | -1.67 [-4.06 to 0.77] |
| Kazakhstan | 7.11 [6.67-7.55] | 4.73 [4.43-5.03] | 6.36 [5.58-7.26] | 3.39 [2.98-3.87] | -0.66 [-1.87 to 0.56] |
| Micronesia (Federated States of) | 0.03 [0.02-0.05] | 4.93 [3.5-6.68] | 0.04 [0.02-0.05] | 4.36 [2.98-5.97] | -1.77 [-4.34 to 0.87] |
| Fiji | 0.3 [0.17-0.4] | 5.6 [3.08-7.45] | 0.4 [0.22-0.55] | 4.95 [2.76-6.78] | -1.53 [-4.55 to 1.6] |
| Brunei Darussalam | 0.11 [0.09-0.14] | 6.25 [5.12-7.71] | 0.19 [0.14-0.23] | 4.81 [3.46-5.79] | -0.49 [-1.88 to 0.92] |
| Mongolia | 0.61 [0.48-0.79] | 3.31 [2.59-4.14] | 0.79 [0.59-1] | 2.65 [1.98-3.34] | -0.8 [-2.36 to 0.79] |
| Bulgaria | 5.51 [5.04-6.01] | 5.32 [4.85-5.84] | 6.96 [5.68-8.32] | 5.81 [4.72-6.98] | 0.11 [-1.07 to 1.31] |
| Marshall Islands | 0.01 [0.01-0.01] | 4.06 [2.85-5.27] | 0.02 [0.01-0.03] | 4.13 [2.75-5.7] | -1.43 [-4.05 to 1.26] |
| Sri Lanka | 7.33 [5.99-8.81] | 5.26 [4.36-6.42] | 8.95 [5.9-12.54] | 3.65 [2.46-5.12] | -0.17 [-2.15 to 1.86] |
| Turkmenistan | 1.25 [1.15-1.36] | 3.79 [3.52-4.05] | 1.57 [1.26-1.97] | 3.19 [2.56-3.99] | -0.77 [-2.08 to 0.55] |
| Armenia | 2.06 [1.97-2.17] | 6.48 [6.22-6.77] | 1.86 [1.64-2.12] | 4.93 [4.37-5.54] | -0.61 [-1.77 to 0.56] |
| Maldives | 0.08 [0.04-0.12] | 5 [3.13-7.15] | 0.11 [0.08-0.16] | 2.92 [2.09-3.78] | -2.04 [-3.69 to -0.36] |
| Myanmar | 24.39 [14.05-36.21] | 7.27 [4.61-10.44] | 25.92 [19.6-33.68] | 4.99 [3.82-6.5] | -1.64 [-3.23 to -0.02] |
| Solomon Islands | 0.09 [0.04-0.13] | 4.13 [2.02-6.06] | 0.21 [0.13-0.28] | 4.15 [2.61-5.65] | -1.6 [-4.21 to 1.08] |
| Papua New Guinea | 1.06 [0.54-1.51] | 3.55 [1.8-5.34] | 2.54 [1.46-3.65] | 3.17 [1.77-4.87] | -1.96 [-4.69 to 0.85] |
| Georgia | 3.42 [3.14-3.7] | 5.92 [5.44-6.42] | 2.79 [2.5-3.12] | 5.56 [5.01-6.2] | -0.35 [-1.58 to 0.89] |
| Timor-Leste | 0.31 [0.18-0.45] | 5.18 [3.5-6.95] | 0.5 [0.38-0.66] | 4.42 [3.3-5.79] | -0.44 [-2.01 to 1.16] |
| Germany | 102.68 [96.16-107.77] | 9.34 [8.75-9.81] | 158.24 [141.31-172.91] | 9.68 [8.88-10.51] | -0.12 [-1.61 to 1.39] |
| Bosnia and Herzegovina | 1.69 [1.4-2.07] | 4.06 [3.4-5.01] | 2.95 [2.1-3.81] | 5.2 [3.76-6.69] | 0.74 [-0.74 to 2.24] |
| Cuba | 6.18 [5.9-6.44] | 5.93 [5.64-6.19] | 8.57 [7.41-9.83] | 5.29 [4.58-6.1] | -0.37 [-1.76 to 1.04] |
| Kyrgyzstan | 1.73 [1.55-1.9] | 4.24 [3.81-4.64] | 1.79 [1.48-2.1] | 2.9 [2.39-3.41] | -1.5 [-2.64 to -0.35] |
| Taiwan (Province of China) | 5.95 [5.67-6.23] | 3.3 [3.14-3.46] | 16.55 [14.96-17.99] | 5.15 [4.67-5.64] | 1.74 [0.23 to 3.27] |
| Cameroon | 1.06 [0.79-1.38] | 1.19 [0.93-1.5] | 3.49 [1.66-5.08] | 1.47 [0.73-2.18] | 0.93 [-0.74 to 2.63] |
| Vanuatu | 0.03 [0.02-0.05] | 3.6 [2.12-4.94] | 0.08 [0.05-0.11] | 3.51 [2.32-4.55] | -1.7 [-4.31 to 0.98] |
| Lao People's Democratic Republic | 2.33 [1.26-3.59] | 7.09 [4.3-10.29] | 3.05 [2.2-4.04] | 5.09 [3.73-6.69] | -1.28 [-2.83 to 0.3] |
| Nepal | 5.54 [3.63-7.49] | 3.5 [2.54-4.63] | 7.66 [5.63-10.49] | 2.94 [2.18-4] | -0.26 [-2.1 to 1.62] |
| Andorra | 0.07 [0.05-0.11] | 14.64 [9.98-21.91] | 0.14 [0.09-0.2] | 10.98 [7.33-15.56] | -0.82 [-2.36 to 0.75] |
| Burkina Faso | 0.99 [0.73-1.3] | 1.13 [0.87-1.38] | 2.6 [1.26-3.62] | 1.4 [0.68-1.91] | 1.03 [-0.54 to 2.63] |
| Guatemala | 2.73 [2.56-2.88] | 3.73 [3.57-3.87] | 6.11 [5.27-7.11] | 4.33 [3.75-5.03] | -0.13 [-1.67 to 1.44] |
| Cambodia | 5.49 [3.45-8] | 7.08 [4.85-9.7] | 8.6 [6.31-11.33] | 5.97 [4.45-7.77] | -0.63 [-2.12 to 0.88] |
| Netherlands | 18.19 [17.02-19.26] | 10.3 [9.58-11.02] | 23.8 [21.21-25.79] | 7.89 [7.14-8.58] | -0.62 [-2.06 to 0.84] |
| Croatia | 4.33 [3.82-4.89] | 7.59 [6.68-8.64] | 7.6 [6.5-8.78] | 9.39 [7.95-11.15] | -0.13 [-1.54 to 1.31] |
| Tajikistan | 2.05 [1.53-2.53] | 4.11 [3.04-5.07] | 2.55 [1.64-3.91] | 2.74 [1.82-4.04] | -1.82 [-3.22 to -0.4] |
| Turkey | 42.4 [31.23-52.36] | 9.35 [7.04-11.25] | 63.62 [48.01-78.5] | 7.28 [5.5-8.99] | -0.77 [-2.18 to 0.67] |
| Democratic People's Republic of Korea | 10.1 [7.07-13.71] | 5.14 [3.62-6.92] | 14.14 [10.19-19.74] | 4.89 [3.5-6.8] | -0.05 [-1.48 to 1.39] |
| Guinea-Bissau | 0.12 [0.08-0.18] | 1.38 [0.99-1.91] | 0.23 [0.13-0.31] | 1.63 [0.92-2.21] | 0.88 [-0.68 to 2.46] |
| Ukraine | 48.5 [45.75-51.38] | 8.1 [7.59-8.61] | 33.6 [24.79-44.12] | 5.3 [4-6.84] | -1.63 [-2.72 to -0.53] |
| Republic of Korea | 19.87 [16.27-24.03] | 5.15 [4.35-6.49] | 30.28 [19.48-37.07] | 4.76 [2.9-6.15] | -0.23 [-1.84 to 1.41] |
| Dominican Republic | 2.4 [1.94-2.89] | 3.81 [3.14-4.92] | 3.62 [2.72-4.72] | 3.46 [2.59-4.52] | 0.09 [-1.22 to 1.4] |
| Kiribati | 0.02 [0.01-0.02] | 3.08 [2.02-3.81] | 0.03 [0.02-0.04] | 3.07 [1.83-4.15] | -1.37 [-4.06 to 1.4] |
| United Kingdom | 85.71 [82.35-87.56] | 11.05 [10.68-11.32] | 98.87 [91.21-103.35] | 8.78 [8.3-9.14] | -0.56 [-2.18 to 1.08] |
| Bolivia (Plurinational State of) | 4.23 [2.9-5.83] | 7.83 [5.51-10.44] | 6.83 [4.76-9.19] | 6.61 [4.61-8.82] | -0.48 [-1.91 to 0.96] |
| Slovenia | 1.86 [1.72-1.99] | 7.96 [7.4-8.5] | 4.53 [3.76-5.36] | 11.06 [9.24-13.1] | 0.63 [-0.94 to 2.23] |
| Mauritius | 0.42 [0.4-0.44] | 4.78 [4.56-5.01] | 0.59 [0.55-0.62] | 3.71 [3.46-3.92] | 0.6 [-1.45 to 2.69] |
| Botswana | 0.23 [0.16-0.31] | 3.07 [2.12-4.17] | 0.5 [0.33-0.72] | 2.97 [2.04-4.21] | -0.17 [-2.75 to 2.48] |
| Pakistan | 33.23 [23.41-42.18] | 3.7 [2.75-4.6] | 66.97 [50.37-89.93] | 3.65 [2.8-4.87] | -0.28 [-2.11 to 1.59] |
| Egypt | 20.08 [16.68-29.37] | 4.85 [3.93-7.45] | 54.73 [36.95-69.63] | 7.22 [5.07-9.06] | 1.97 [0.69 to 3.28] |
| Ireland | 3.9 [3.67-4.15] | 9.82 [9.21-10.45] | 5.56 [5.05-6.13] | 7.88 [7.18-8.7] | -0.52 [-2.01 to 1] |
| Côte d'Ivoire | 0.81 [0.56-1.04] | 0.87 [0.6-1.08] | 1.77 [0.99-2.68] | 0.86 [0.51-1.23] | -0.01 [-1.65 to 1.65] |
| Czechia | 10.75 [10.17-11.43] | 8.35 [7.9-8.87] | 17.94 [15.2-20.87] | 8.92 [7.54-10.4] | -0.36 [-1.66 to 0.97] |
| Yemen | 5.89 [3.5-9.36] | 6.44 [3.92-9.48] | 12.47 [6.89-18.15] | 5.94 [3.21-8.67] | -0.39 [-1.59 to 0.83] |
| Serbia | 5.96 [4.79-7.09] | 5.9 [4.83-7.12] | 10.01 [7.57-12.53] | 6.53 [4.92-8.15] | -0.26 [-1.67 to 1.16] |
| Ghana | 3.06 [1.59-4.17] | 2.37 [1.31-3.16] | 3.86 [2.65-5.6] | 1.43 [1.04-2.08] | -2.32 [-4.03 to -0.58] |
| Mauritania | 0.18 [0.14-0.23] | 1.05 [0.84-1.29] | 0.43 [0.22-0.65] | 1.31 [0.68-1.84] | 0.59 [-1.23 to 2.44] |
| Norway | 4.44 [4.22-4.62] | 7.55 [7.19-7.92] | 7.19 [6.48-7.71] | 8.13 [7.46-8.7] | 0.16 [-1.32 to 1.66] |
| Guam | 0.04 [0.03-0.05] | 4.09 [3.38-5.08] | 0.06 [0.05-0.07] | 3.15 [2.47-3.83] | -2.79 [-5.31 to -0.21] |
| Niger | 0.93 [0.61-1.32] | 1.13 [0.86-1.46] | 2.37 [1.07-3.73] | 1.23 [0.58-1.96] | 0.54 [-1.1 to 2.21] |
| Palau | 0 [0-0] | 2.79 [1.98-3.68] | 0 [0-0.01] | 2.57 [1.94-3.16] | -0.12 [-1.13 to 0.9] |
| Togo | 0.31 [0.23-0.4] | 1.03 [0.78-1.27] | 0.85 [0.4-1.24] | 1.4 [0.69-1.97] | 1.25 [-0.42 to 2.94] |
| Seychelles | 0.04 [0.04-0.05] | 6.83 [5.87-8.56] | 0.06 [0.05-0.08] | 5.45 [4.36-6.61] | -0.42 [-1.46 to 0.63] |
| Kuwait | 0.66 [0.59-0.74] | 6.19 [5.61-6.83] | 1.46 [1.19-1.74] | 4.51 [3.68-5.39] | -0.98 [-2.24 to 0.3] |
| Tuvalu | 0 [0-0] | 4.6 [3.27-6.15] | 0 [0-0.01] | 3.81 [2.57-4.82] | -1.88 [-4.49 to 0.79] |
| Gambia | 0.05 [0.03-0.07] | 0.58 [0.41-0.74] | 0.11 [0.07-0.15] | 0.61 [0.42-0.79] | -0.06 [-1.71 to 1.63] |
| Zambia | 1.72 [1.29-2.36] | 2.67 [2.15-3.45] | 3.59 [2.19-5.12] | 2.8 [1.82-3.79] | -0.16 [-1.53 to 1.22] |
| Somalia | 1.42 [0.89-2.09] | 2.66 [1.86-3.71] | 3.64 [2.32-5.25] | 2.91 [1.93-4.06] | 0.43 [-0.8 to 1.68] |
| Liberia | 0.26 [0.18-0.38] | 1.11 [0.84-1.43] | 0.55 [0.25-0.81] | 1.4 [0.66-1.99] | 1.07 [-0.57 to 2.74] |
| Eritrea | 0.61 [0.43-0.89] | 2.5 [1.89-3.42] | 1.29 [0.79-1.85] | 2.88 [1.85-3.89] | 0.42 [-0.86 to 1.72] |
| Guyana | 0.2 [0.18-0.23] | 3.5 [3.13-3.87] | 0.24 [0.19-0.32] | 3.46 [2.68-4.45] | 0.44 [-0.81 to 1.71] |
| Lithuania | 3.96 [3.7-4.22] | 9.44 [8.81-10.08] | 3.93 [3.36-4.49] | 7.89 [6.74-8.93] | -0.89 [-2.22 to 0.46] |
| Guinea | 0.31 [0.21-0.41] | 0.47 [0.34-0.59] | 0.48 [0.25-0.83] | 0.42 [0.25-0.64] | -0.12 [-1.81 to 1.6] |
| Niue | 0 [0-0] | 3.81 [2.62-4.99] | 0 [0-0] | 5.71 [4.22-7.06] | -1.2 [-3.78 to 1.44] |
| Nicaragua | 1.6 [1.35-2] | 4.33 [3.71-5.4] | 2.23 [1.6-2.73] | 3.79 [2.76-4.62] | 0.01 [-1.36 to 1.4] |
| Saint Lucia | 0.05 [0.05-0.06] | 5.19 [4.91-5.53] | 0.09 [0.07-0.11] | 4.09 [3.38-4.98] | -1.06 [-2.06 to -0.04] |
| Angola | 1.66 [1.08-2.41] | 2.2 [1.59-2.89] | 4.23 [2.68-6.07] | 2.09 [1.23-3.17] | -0.14 [-2.04 to 1.8] |
| India | 213.95 [167.38-261.36] | 3.17 [2.54-3.77] | 340.76 [274.88-412.19] | 2.71 [2.18-3.28] | -0.45 [-2.03 to 1.16] |
| Slovakia | 4.08 [3.41-5.01] | 7.03 [5.88-8.6] | 6.46 [4.67-8.19] | 7.45 [5.39-9.48] | -0.38 [-2.02 to 1.29] |
| Argentina | 20.49 [19.7-21.27] | 6.3 [6.05-6.53] | 26.72 [24.65-28.76] | 5.18 [4.79-5.61] | -0.77 [-1.91 to 0.38] |
| United States of America | 406.29 [386.01-417.4] | 13.5 [12.91-13.84] | 520.6 [475-543.85] | 9.81 [9.11-10.22] | -1.08 [-2.74 to 0.61] |
| Uganda | 2.58 [1.81-3.37] | 1.82 [1.32-2.3] | 6.23 [3.51-9.52] | 1.99 [1.28-2.76] | 0 [-1.29 to 1.31] |
| Republic of Moldova | 2.52 [2.4-2.66] | 5.69 [5.4-6.01] | 1.85 [1.66-2.09] | 3.71 [3.27-4.25] | -1.17 [-2.58 to 0.26] |
| Madagascar | 2.07 [1.62-2.74] | 2.13 [1.66-2.67] | 4.21 [2.78-5.82] | 2.11 [1.37-2.91] | 0.03 [-1.32 to 1.4] |
| Iraq | 9.33 [7.19-12.81] | 7.08 [5.63-9.86] | 21.87 [16.33-28.61] | 7.28 [5.46-9.31] | 0.1 [-1.25 to 1.47] |
| Morocco | 2.59 [1.92-3.31] | 1.32 [0.98-1.63] | 4.27 [2.92-5.51] | 1.24 [0.85-1.58] | -0.17 [-1.45 to 1.12] |
| Namibia | 0.2 [0.15-0.25] | 2.23 [1.76-2.81] | 0.41 [0.29-0.57] | 2.39 [1.71-3.29] | -0.13 [-2.69 to 2.5] |
| Bhutan | 0.16 [0.1-0.22] | 3.46 [2.31-4.75] | 0.19 [0.13-0.3] | 2.97 [1.98-4.48] | -0.48 [-2.39 to 1.47] |
| New Zealand | 4.19 [3.93-4.42] | 10.99 [10.3-11.6] | 7.66 [6.99-8.26] | 9.98 [9.19-10.72] | -0.23 [-1.59 to 1.14] |
| Jordan | 1.83 [1.48-2.26] | 8.07 [6.36-10.07] | 5.71 [4.08-7.49] | 6.45 [4.61-8.34] | -0.36 [-1.95 to 1.25] |
| Russian Federation | 94.43 [92.2-96.71] | 5.68 [5.54-5.82] | 125.6 [116.3-135.07] | 5.98 [5.57-6.42] | -0.24 [-1.37 to 0.91] |
| Malta | 0.3 [0.27-0.32] | 7.4 [6.8-8.11] | 0.56 [0.48-0.63] | 7.93 [6.81-9.06] | -0.2 [-1.65 to 1.26] |
| Libya | 1.99 [1.5-2.54] | 7.07 [5.04-9.03] | 4.45 [3.2-5.92] | 7.9 [5.65-10.54] | 0.72 [-0.69 to 2.14] |
| Antigua and Barbuda | 0.03 [0.02-0.03] | 4.66 [4.32-5.03] | 0.04 [0.04-0.05] | 4.47 [4.17-4.76] | -0.26 [-1.85 to 1.35] |
| Spain | 46.63 [43.88-49.18] | 10.26 [9.61-10.97] | 70.22 [61.43-76.96] | 9.29 [8.3-10.23] | -0.67 [-1.99 to 0.66] |
| Monaco | 0.09 [0.07-0.13] | 20.64 [14.89-27.98] | 0.16 [0.12-0.21] | 25.09 [18.21-32.83] | 0.65 [-0.91 to 2.22] |
| Kenya | 2.79 [2.07-3.63] | 1.81 [1.34-2.66] | 6.84 [4.91-9.29] | 2.16 [1.59-2.88] | 0.84 [-0.4 to 2.1] |
| Congo | 0.38 [0.28-0.51] | 2.38 [1.84-3.04] | 0.83 [0.53-1.16] | 2.26 [1.45-3.18] | -0.35 [-2.28 to 1.62] |
| South Africa | 8.7 [6.54-10.28] | 3.27 [2.31-4.08] | 17.87 [12.61-20.63] | 3.71 [2.57-4.28] | 0.03 [-2.4 to 2.53] |
| Venezuela (Bolivarian Republic of) | 7.4 [7.12-7.66] | 4.69 [4.5-4.86] | 13.43 [9.99-17.61] | 4.89 [3.64-6.4] | -0.24 [-1.58 to 1.13] |
| Senegal | 0.8 [0.62-1.02] | 1.12 [0.85-1.38] | 1.66 [0.82-2.53] | 1.38 [0.72-2.02] | 0.98 [-0.77 to 2.75] |
| Estonia | 1.91 [1.78-2.03] | 10.16 [9.5-10.86] | 2.1 [1.76-2.44] | 8.85 [7.45-10.19] | -0.92 [-2.37 to 0.56] |
| Saudi Arabia | 3.37 [2.36-5.33] | 3.24 [2.17-5.59] | 12.87 [8.98-20.63] | 4.81 [3.55-7.31] | 1.53 [0.53 to 2.55] |
| Colombia | 14.62 [13.8-15.44] | 5.45 [5.18-5.71] | 27.01 [22.68-32.16] | 5.35 [4.48-6.36] | -0.36 [-1.67 to 0.95] |
| Sao Tome and Principe | 0.01 [0.01-0.01] | 0.82 [0.61-1] | 0.01 [0.01-0.02] | 0.75 [0.55-1.03] | -0.3 [-2.04 to 1.46] |
| Grenada | 0.04 [0.04-0.04] | 5.11 [4.62-5.64] | 0.06 [0.05-0.06] | 5.27 [4.6-5.9] | 0.13 [-0.93 to 1.2] |
| Saint Vincent and the Grenadines | 0.05 [0.05-0.05] | 5.64 [5.18-6.07] | 0.06 [0.06-0.07] | 4.91 [4.3-5.62] | -0.37 [-1.41 to 0.68] |
| Malawi | 0.85 [0.57-1.1] | 0.9 [0.65-1.1] | 1.13 [0.59-1.9] | 0.82 [0.5-1.21] | -0.33 [-1.67 to 1.03] |
| Tunisia | 2.53 [1.9-3.1] | 3.84 [2.85-4.78] | 4.42 [3-6.07] | 3.56 [2.4-4.88] | -0.43 [-1.75 to 0.9] |
| Lesotho | 0.26 [0.19-0.35] | 2.56 [1.85-3.4] | 0.56 [0.4-0.76] | 4.35 [3.19-5.78] | 2.4 [-0.14 to 5] |
| Canada | 38.44 [36.27-40.42] | 12.66 [11.9-13.41] | 61.65 [55.89-67.24] | 9.76 [8.97-10.55] | -0.65 [-2.3 to 1.03] |
| Haiti | 4.06 [2.11-6.37] | 7.34 [4.56-10.52] | 6.11 [3.79-9.14] | 5.89 [3.82-8.38] | -0.5 [-1.62 to 0.64] |
| Japan | 93 [89.15-96.74] | 6.85 [6.49-7.31] | 136.47 [120.5-146.07] | 5.69 [5.28-6.02] | -0.79 [-2.31 to 0.76] |
| Denmark | 8.23 [7.74-8.66] | 11.39 [10.74-11.95] | 11.54 [10.29-12.73] | 10.97 [9.87-11.96] | -0.18 [-1.52 to 1.18] |
| American Samoa | 0.01 [0.01-0.01] | 3.66 [2.92-4.46] | 0.02 [0.01-0.02] | 3.82 [2.89-4.76] | 0.49 [-2.38 to 3.45] |
| Luxembourg | 0.58 [0.54-0.61] | 11.96 [11.27-12.66] | 0.97 [0.87-1.08] | 10.19 [9.14-11.48] | -0.88 [-2.32 to 0.58] |
| Chile | 5.82 [5.55-6.06] | 5.07 [4.83-5.29] | 10.77 [9.86-11.63] | 4.85 [4.45-5.26] | 0 [-1.22 to 1.22] |
| Paraguay | 1.44 [1.15-1.73] | 4.39 [3.52-5.34] | 3.29 [2.41-4.24] | 5.27 [3.86-6.8] | 0.77 [-0.47 to 2.04] |
| Chad | 0.53 [0.39-0.73] | 0.93 [0.71-1.18] | 2.01 [1.05-2.97] | 1.4 [0.74-1.98] | 1.66 [0.04 to 3.3] |
| Poland | 31.86 [29.74-33.9] | 7.57 [7.05-8.03] | 50.98 [46.22-56.12] | 7.64 [6.95-8.37] | -0.78 [-2.1 to 0.57] |
| Peru | 9.55 [7.83-12.4] | 5.11 [4.23-6.53] | 19.29 [12.18-25.24] | 5.56 [3.52-7.28] | 0.56 [-0.94 to 2.09] |
| Tokelau | 0 [0-0] | 4.1 [2.69-5.43] | 0 [0-0] | 6.26 [3.87-9.04] | -1.57 [-4.18 to 1.12] |
| North Macedonia | 1.03 [0.85-1.28] | 5.53 [4.59-6.92] | 1.88 [1.36-2.45] | 6.17 [4.55-7.96] | -0.12 [-7.69 to 8.07] |
| Dominica | 0.04 [0.03-0.04] | 5.52 [4.64-6.55] | 0.05 [0.03-0.06] | 6.21 [4.62-7.87] | 0.32 [-0.88 to 1.52] |
| Algeria | 6.74 [4.98-8.31] | 3.49 [2.58-4.24] | 10.89 [7.98-14.32] | 2.94 [2.12-3.81] | -0.24 [-1.76 to 1.31] |
| Central African Republic | 0.48 [0.33-0.67] | 2.46 [1.81-3.16] | 0.85 [0.53-1.22] | 2.33 [1.49-3.16] | -0.11 [-1.9 to 1.71] |
| Belize | 0.05 [0.05-0.06] | 3.22 [3.03-3.45] | 0.12 [0.1-0.13] | 3.31 [2.93-3.71] | 0.3 [-0.83 to 1.45] |
| Eswatini | 0.15 [0.11-0.21] | 3.4 [2.6-4.56] | 0.3 [0.2-0.45] | 4.06 [2.76-6.01] | 0.77 [-1.74 to 3.34] |
| Italy | 93.84 [89.08-97.78] | 13.23 [12.44-14.05] | 126.36 [112.96-136.25] | 10.99 [10.17-11.83] | -1.15 [-2.54 to 0.26] |
| Jamaica | 0.79 [0.73-0.87] | 3.71 [3.45-4.04] | 1.23 [0.96-1.58] | 4.16 [3.24-5.4] | 0.72 [-0.63 to 2.1] |
| Saint Kitts and Nevis | 0.02 [0.02-0.02] | 5.25 [4.93-5.61] | 0.02 [0.02-0.03] | 4.02 [3.37-4.72] | -0.23 [-1.57 to 1.14] |
| San Marino | 0.06 [0.05-0.08] | 22.51 [17.59-30.06] | 0.06 [0.04-0.09] | 13.01 [8.21-18.52] | -0.95 [-2.43 to 0.55] |
| Portugal | 9.27 [8.8-9.72] | 7.77 [7.35-8.21] | 15.82 [13.78-17.35] | 8.05 [7.17-8.94] | -0.1 [-1.55 to 1.38] |
| Cabo Verde | 0.08 [0.06-0.11] | 2.42 [1.91-3.37] | 0.17 [0.12-0.21] | 3.45 [2.55-4.39] | 0.7 [-1.03 to 2.46] |
| Israel | 5.22 [4.83-5.59] | 10.66 [9.85-11.44] | 11.77 [10.54-12.89] | 9.84 [8.89-10.77] | -0.23 [-1.94 to 1.5] |
| Bahamas | 0.08 [0.08-0.09] | 4.15 [3.85-4.49] | 0.16 [0.13-0.2] | 4.06 [3.26-4.96] | -0.17 [-1.3 to 0.97] |
| Suriname | 0.12 [0.09-0.15] | 3.85 [2.93-4.54] | 0.21 [0.16-0.28] | 3.58 [2.68-4.61] | -0.18 [-1.24 to 0.9] |
| Zimbabwe | 1.76 [1.29-2.19] | 3.08 [2.38-3.9] | 4.25 [2.98-5.61] | 4.26 [3.14-5.54] | 1.78 [-0.87 to 4.49] |
| Iceland | 0.25 [0.23-0.27] | 9.15 [8.39-9.89] | 0.44 [0.39-0.49] | 8.82 [7.83-9.85] | -0.24 [-1.65 to 1.18] |
| Cyprus | 0.68 [0.54-0.95] | 9.38 [7.38-13.39] | 1.92 [1.29-2.39] | 10.45 [7.02-12.96] | 0.8 [-1.16 to 2.81] |
| Benin | 0.51 [0.38-0.68] | 1.1 [0.88-1.36] | 1.47 [0.7-2.09] | 1.34 [0.67-1.88] | 0.84 [-0.86 to 2.58] |
| Democratic Republic of the Congo | 5.29 [3.88-7.22] | 1.95 [1.36-2.6] | 10.96 [6.87-15.7] | 1.97 [1.17-3] | 0.16 [-1.74 to 2.09] |
| Bermuda | 0.04 [0.04-0.04] | 6.49 [6.01-6.97] | 0.06 [0.05-0.07] | 5.5 [4.5-6.77] | -0.2 [-1.42 to 1.03] |
| Equatorial Guinea | 0.07 [0.05-0.1] | 2.22 [1.59-2.84] | 0.18 [0.09-0.3] | 2.14 [1.14-3.5] | -0.44 [-2.41 to 1.57] |
| Afghanistan | 8.61 [4.47-13.17] | 10.49 [5.7-15.83] | 18.2 [10.67-26.25] | 9.95 [5.88-14.56] | -0.23 [-1.5 to 1.06] |
| Mexico | 37.29 [35.96-38.82] | 5.04 [4.9-5.18] | 65.6 [59.2-72.18] | 5.17 [4.66-5.68] | -0.3 [-1.85 to 1.26] |
| France | 77.29 [72.67-81.79] | 10.46 [9.83-11.13] | 126.05 [110.39-141.22] | 10.35 [9.21-11.5] | 0.05 [-1.2 to 1.33] |
| Oman | 0.52 [0.37-0.7] | 4.64 [3.22-6.27] | 1.01 [0.74-1.33] | 3.93 [2.89-5.12] | -0.47 [-2.06 to 1.16] |
| Rwanda | 1.78 [1.3-2.4] | 3.14 [2.43-4.08] | 2.5 [1.55-3.81] | 2.7 [1.73-3.99] | -1.2 [-2.46 to 0.08] |
| Iran (Islamic Republic of) | 34.51 [21.14-44.26] | 7.99 [4.88-9.87] | 53.18 [32.59-63.71] | 6.77 [4.16-8.18] | -0.25 [-1.44 to 0.96] |
| Syrian Arab Republic | 7.63 [5.82-9.66] | 8.8 [6.44-10.95] | 10.27 [7.28-14.01] | 7.84 [5.6-10.58] | -0.67 [-1.94 to 0.63] |
| Qatar | 0.15 [0.1-0.19] | 8.21 [5.7-10.45] | 0.85 [0.52-1.19] | 6.17 [4.06-8.34] | -1.14 [-2.35 to 0.08] |
| South Sudan | 1.17 [0.8-1.75] | 2.48 [1.8-3.46] | 2.24 [1.47-3.1] | 3.15 [2.07-4.34] | 0.82 [-0.55 to 2.21] |
| Lebanon | 1.6 [1.09-2.37] | 6.79 [4.66-9.87] | 4.12 [3.07-5.3] | 6.96 [5.13-9] | 0.26 [-1.05 to 1.6] |
| Sudan | 11.05 [6.54-16.56] | 6.86 [4.47-9.67] | 19.32 [12.04-27.62] | 6.22 [3.85-8.91] | -0.36 [-1.65 to 0.95] |
| Ethiopia | 36.84 [19-55.63] | 10.43 [6.89-14.23] | 50.45 [36.39-73.58] | 7.09 [5.01-10.99] | -1.73 [-3.23 to -0.22] |
| Panama | 0.92 [0.86-0.99] | 4.39 [4.13-4.68] | 2.39 [1.97-2.87] | 5.56 [4.6-6.69] | 0.45 [-0.91 to 1.83] |
| United Republic of Tanzania | 5.73 [4.55-7.31] | 2.63 [2.1-3.33] | 11.69 [7.4-17.17] | 2.67 [1.72-3.84] | 0.02 [-1.33 to 1.39] |
| Montenegro | 0.38 [0.3-0.46] | 6.17 [4.93-7.51] | 0.62 [0.47-0.85] | 6.88 [5.2-9.23] | 0.25 [-1.28 to 1.81] |
| Brazil | 54.36 [52.59-56.33] | 4.64 [4.46-4.78] | 101.32 [96.01-105.62] | 4.28 [4.05-4.49] | -0.44 [-1.69 to 0.83] |
| Belarus | 9.15 [8.56-9.68] | 7.74 [7.23-8.22] | 11.61 [9.49-14.14] | 8.09 [6.63-9.79] | -0.41 [-1.69 to 0.89] |
| Northern Mariana Islands | 0.01 [0.01-0.02] | 4.82 [3.24-6.26] | 0.02 [0.01-0.02] | 3.83 [2.89-4.49] | -0.79 [-4.72 to 3.32] |
| Costa Rica | 1.45 [1.36-1.53] | 6.15 [5.77-6.5] | 3.75 [3.35-4.16] | 7.46 [6.64-8.3] | 0.29 [-1.04 to 1.63] |
| Bangladesh | 36.76 [23.54-52.63] | 4.06 [2.95-5.25] | 42.36 [29.63-57.66] | 2.85 [1.99-3.89] | -1.05 [-2.8 to 0.74] |
| Finland | 4.85 [4.57-5.09] | 7.44 [7.05-7.81] | 8.02 [7.1-8.77] | 7.75 [7.07-8.45] | -0.02 [-1.35 to 1.33] |
| Singapore | 1.41 [1.32-1.5] | 5.55 [5.18-5.91] | 3.13 [2.88-3.39] | 5.07 [4.6-5.56] | -0.22 [-2.13 to 1.72] |
| United States Virgin Islands | 0.05 [0.03-0.06] | 4.88 [3.74-5.95] | 0.05 [0.04-0.07] | 3.58 [2.57-4.72] | -0.78 [-2.42 to 0.88] |
| Burundi | 1.23 [0.9-1.67] | 2.71 [2.05-3.63] | 2.19 [1.21-3.4] | 2.51 [1.5-3.75] | -0.37 [-1.62 to 0.9] |
| Greece | 16.59 [15.67-17.44] | 12.77 [11.99-13.65] | 23 [20.96-24.7] | 11.53 [10.66-12.3] | -0.86 [-1.92 to 0.2] |
| Sierra Leone | 0.44 [0.31-0.63] | 1.04 [0.77-1.35] | 0.94 [0.45-1.35] | 1.33 [0.64-1.87] | 1.18 [-0.46 to 2.84] |
| Ecuador | 3.85 [3.63-4.09] | 4.77 [4.49-5.05] | 9.98 [8.16-12.08] | 5.8 [4.75-7.03] | 0.85 [-0.62 to 2.33] |
| El Salvador | 2.46 [2.13-3.06] | 5.16 [4.52-6.45] | 3.64 [2.61-4.52] | 5.74 [4.12-7.14] | 0.47 [-1.05 to 2.01] |
| Hungary | 11.24 [10.51-11.95] | 8.28 [7.74-8.84] | 14.29 [12.05-16.9] | 8.28 [6.94-9.92] | -0.67 [-2.01 to 0.69] |
| Comoros | 0.09 [0.06-0.13] | 2.5 [1.81-3.36] | 0.16 [0.11-0.23] | 2.75 [1.89-3.86] | 0.04 [-1.38 to 1.47] |
| Romania | 11.85 [11.31-12.41] | 4.73 [4.52-4.95] | 18.32 [15.89-20.71] | 5.76 [4.95-6.52] | 0.32 [-0.93 to 1.59] |
| Puerto Rico | 2.31 [2.18-2.44] | 6.42 [6.08-6.79] | 3.36 [2.73-3.97] | 5.93 [4.92-6.91] | -0.37 [-1.66 to 0.95] |
| Barbados | 0.15 [0.14-0.16] | 5.52 [5.22-5.84] | 0.24 [0.19-0.3] | 5.6 [4.43-7.02] | 0.22 [-1.05 to 1.51] |
| Mozambique | 4.83 [3.63-6.36] | 3.85 [3.1-4.75] | 8.15 [4.94-13.18] | 3.81 [2.74-5.2] | 0.36 [-1 to 1.74] |
| Sweden | 12.65 [11.76-13.42] | 10.52 [9.73-11.36] | 15.32 [13.46-17.22] | 8.26 [7.35-9.2] | -0.39 [-2.04 to 1.29] |
| Austria | 10.03 [9.44-10.62] | 9.68 [9.1-10.32] | 14.96 [13.09-16.55] | 9.3 [8.22-10.2] | -0.16 [-1.59 to 1.3] |
| Mali | 0.86 [0.58-1.13] | 1.01 [0.73-1.23] | 1.57 [0.94-2.52] | 0.83 [0.54-1.14] | -0.51 [-2.18 to 1.17] |
| Latvia | 2.92 [2.71-3.12] | 8.99 [8.34-9.61] | 2.75 [2.32-3.24] | 7.99 [6.76-9.4] | -1.07 [-2.48 to 0.35] |
| United Arab Emirates | 0.68 [0.49-0.9] | 8.82 [6.06-12.02] | 2.98 [2.08-4.09] | 6.52 [4.88-8.54] | -0.05 [-1.32 to 1.25] |
| Djibouti | 0.07 [0.04-0.1] | 2.24 [1.5-3.28] | 0.23 [0.13-0.39] | 2.72 [1.6-4.25] | 0.58 [-0.82 to 2] |
| Palestine | 1.19 [0.91-1.58] | 8.87 [6.74-11.55] | 2.4 [1.92-3.13] | 7.13 [5.74-9] | -0.78 [-2.17 to 0.62] |
| Greenland | 0.02 [0.01-0.02] | 4.09 [3.16-4.86] | 0.02 [0.01-0.02] | 2.86 [2.12-3.79] | -0.97 [-2.26 to 0.33] |
| Bahrain | 0.2 [0.15-0.24] | 8.2 [6.05-9.86] | 0.56 [0.39-0.75] | 5.64 [4.1-7.3] | -1.58 [-3.59 to 0.48] |
| Belgium | 14.56 [13.45-15.61] | 11.09 [10.16-12.02] | 18.89 [16.34-21.05] | 9.67 [8.55-10.77] | -0.82 [-2.06 to 0.43] |
| Trinidad and Tobago | 0.5 [0.47-0.52] | 4.93 [4.7-5.16] | 0.73 [0.56-0.93] | 4.37 [3.38-5.53] | -0.62 [-1.78 to 0.55] |
| Gabon | 0.16 [0.12-0.2] | 2.15 [1.56-2.79] | 0.3 [0.18-0.44] | 2.29 [1.41-3.33] | 0.07 [-1.92 to 2.1] |
| Cook Islands | 0 [0-0] | 1.78 [1.29-2.26] | 0 [0-0] | 1.34 [0.95-1.75] | -2.53 [-4.82 to -0.19] |
| Uruguay | 2.59 [2.44-2.74] | 7.15 [6.77-7.56] | 3.63 [3.31-3.93] | 7.37 [6.76-7.98] | -0.23 [-1.47 to 1.01] |
| Nauru | 0 [0-0.01] | 5.49 [3.98-7.33] | 0 [0-0.01] | 5.2 [3.43-7.45] | -1.79 [-4.4 to 0.89] |
| Australia | 19.78 [18.73-20.87] | 10.57 [10.02-11.19] | 44.29 [39.62-48.39] | 10.72 [9.79-11.73] | -0.54 [-1.97 to 0.9] |
| Honduras | 2.28 [1.86-2.78] | 5.54 [4.43-6.75] | 3.91 [2.8-5.45] | 4.98 [3.6-6.68] | -0.04 [-1.38 to 1.32] |
| Switzerland | 11.3 [10.61-11.92] | 13.38 [12.52-14.34] | 15.2 [13.25-17.12] | 10.28 [9.05-11.46] | -0.89 [-2.37 to 0.61] |
| Nigeria | 9.43 [6.67-13.27] | 1.13 [0.8-1.49] | 21.09 [11.23-29.09] | 1.1 [0.63-1.46] | -0.17 [-1.89 to 1.58] |

| **Country** | **1990** | | **2021** | | **1990-2021** |
| --- | --- | --- | --- | --- | --- |
|  | **Prevalence cases** | **ASPR per 100,000** | **Prevalence cases** | **ASPR per 100,000** | **EAPC** |
|  | **No. *10^2^ (95% UI)** | **No. (95% UI)** | **No. *10^2^ (95% UI)** | **No.(95% UI)** | **No. (95% CI)** |
| Philippines | 45.93 [36.61-56.52] | 8.17 [6.41-9.62] | 82.89 [64.52-98.28] | 7.88 [6.16-9.31] | -0.3 [-1.6 to 1.02] |
| Myanmar | 43.61 [22.39-74.74] | 12.29 [6.77-20.76] | 53.42 [38.32-78.59] | 9.92 [7.13-14.72] | -1.04 [-2.63 to 0.57] |
| Maldives | 0.16 [0.09-0.24] | 9.26 [5.53-13.44] | 0.35 [0.25-0.47] | 8.63 [6.02-11.68] | -0.28 [-2.06 to 1.54] |
| Malaysia | 17.7 [13.92-22.21] | 12.32 [10.13-16.53] | 43.56 [34.81-61.67] | 14.2 [11.39-20.35] | 0.75 [-1.14 to 2.68] |
| Lao People's Democratic Republic | 3.95 [1.96-6.6] | 11.43 [6.15-18.43] | 6.12 [4.23-8.95] | 9.37 [6.49-14.02] | -0.77 [-2.3 to 0.78] |
| Cambodia | 9.66 [5.51-15.44] | 11.71 [7.28-18.17] | 17.56 [12.86-25.65] | 11.35 [8.3-16.8] | -0.07 [-1.54 to 1.41] |
| Indonesia | 135.41 [96.81-204.49] | 8.6 [6.21-13.01] | 255.1 [202.77-375.47] | 9.5 [7.5-14.17] | 0.29 [-1.29 to 1.89] |
| Vanuatu | 0.06 [0.04-0.09] | 5.76 [3.21-8.62] | 0.15 [0.1-0.2] | 5.74 [3.9-8.04] | -1.74 [-4.26 to 0.85] |
| Tonga | 0.04 [0.03-0.06] | 5.51 [3.53-9] | 0.05 [0.04-0.08] | 5.68 [3.91-8.69] | -1.41 [-3.63 to 0.87] |
| Samoa | 0.11 [0.08-0.17] | 8.66 [6.42-13.9] | 0.15 [0.1-0.23] | 8.32 [6.06-13.42] | -1.45 [-3.64 to 0.8] |
| Solomon Islands | 0.16 [0.07-0.24] | 6.62 [2.72-10.31] | 0.38 [0.23-0.55] | 6.83 [4.26-10.03] | -1.5 [-4.03 to 1.09] |
| Taiwan (Province of China) | 14.09 [12.57-15.63] | 7.53 [6.69-8.37] | 47.24 [41.8-53.14] | 18.21 [15.56-21.07] | 3 [1.66 to 4.36] |
| China | 1979.7 [1507.86-2369.09] | 18.04 [13.83-21.68] | 5316.61 [3527.29-6651.54] | 40.09 [26.3-52.58] | 2.02 [-0.13 to 4.21] |
| Papua New Guinea | 1.84 [0.88-2.73] | 5.52 [2.54-8.94] | 4.66 [2.67-6.89] | 5.13 [2.88-8.06] | -2 [-4.58 to 0.65] |
| Democratic People's Republic of Korea | 23.28 [15.79-32.08] | 11.34 [7.73-15.5] | 40.38 [26.67-57.82] | 14.47 [9.55-20.63] | 0.77 [-0.88 to 2.45] |
| Micronesia (Federated States of) | 0.06 [0.04-0.09] | 7.52 [5.09-10.93] | 0.07 [0.05-0.09] | 7.06 [4.96-9.92] | -1.63 [-4.05 to 0.86] |
| Marshall Islands | 0.02 [0.01-0.03] | 6.31 [4.09-9.2] | 0.03 [0.02-0.05] | 6.73 [4.48-9.56] | -1.39 [-3.89 to 1.17] |
| Fiji | 0.47 [0.26-0.64] | 7.1 [3.82-9.51] | 0.64 [0.35-0.93] | 7.3 [3.98-10.5] | -1.1 [-3.67 to 1.54] |
| Kiribati | 0.03 [0.02-0.04] | 4.62 [2.78-5.95] | 0.05 [0.03-0.08] | 4.84 [2.73-6.92] | -1.47 [-4.07 to 1.21] |
| Thailand | 52.43 [38.72-63.41] | 11.13 [8-13.57] | 116.02 [79.22-148.24] | 15.86 [11.06-19.96] | 0.89 [-0.35 to 2.16] |
| Tajikistan | 4.76 [3.3-6.01] | 9.56 [6.68-12.19] | 6.7 [4.15-10.6] | 6.9 [4.41-10.64] | -1.54 [-3.19 to 0.14] |
| Mongolia | 1.11 [0.84-1.45] | 5.77 [4.37-7.4] | 1.77 [1.29-2.25] | 5.56 [4.08-7.1] | 0.01 [-1.57 to 1.61] |
| Timor-Leste | 0.56 [0.29-0.89] | 8.52 [5.18-12.97] | 1 [0.73-1.39] | 8.28 [6.15-11.81] | 0.03 [-1.53 to 1.61] |
| Viet Nam | 35.58 [27.25-48.63] | 6.36 [4.89-8.73] | 77.35 [55.84-108.23] | 7.91 [5.69-10.99] | 0.91 [-1.52 to 3.4] |
| Sri Lanka | 17.07 [12.94-20.44] | 11.59 [8.73-13.97] | 24.71 [15.45-38.19] | 10.44 [6.71-16.1] | 1.02 [-1.03 to 3.1] |
| Turkmenistan | 3 [2.71-3.35] | 9.08 [8.29-9.95] | 4.48 [3.52-5.7] | 8.89 [7.03-11.28] | -0.15 [-1.71 to 1.44] |
| Kazakhstan | 18.27 [16.14-20.17] | 11.94 [10.53-13.13] | 21.32 [17.87-25.39] | 11.06 [9.29-13.16] | 0.29 [-1.23 to 1.84] |
| Georgia | 11.72 [10.44-13.05] | 20.01 [17.9-22.29] | 9.16 [8.13-10.31] | 18.67 [16.68-20.89] | -0.58 [-2.33 to 1.2] |
| Kyrgyzstan | 4.78 [4.23-5.33] | 11.9 [10.59-13.16] | 5.61 [4.63-6.62] | 8.78 [7.29-10.32] | -0.99 [-2.48 to 0.53] |
| Afghanistan | 13.47 [6.11-21.42] | 15.85 [7.57-25.01] | 35.45 [20.4-51.52] | 16.86 [9.81-24.75] | 0.16 [-1.09 to 1.41] |
| United Kingdom | 355.04 [340.67-366.81] | 49.92 [47.87-51.86] | 398.94 [373.9-416.7] | 40.54 [38.52-42.39] | -0.19 [-2.12 to 1.77] |
| Azerbaijan | 8.31 [6.3-10.11] | 11.96 [9-14.59] | 12.4 [8.36-17.99] | 12.01 [8.1-17.33] | -0.07 [-1.69 to 1.58] |
| Armenia | 5.79 [5.42-6.21] | 17.75 [16.62-19.02] | 6.68 [5.88-7.54] | 18.26 [15.93-20.51] | 0.32 [-1.29 to 1.96] |
| France | 278.88 [257.29-298.81] | 41.12 [37.47-45.12] | 529.04 [456.37-607.61] | 51.71 [45.64-58.3] | 0.91 [-0.71 to 2.55] |
| Jordan | 3.65 [2.85-4.63] | 12.86 [9.92-16.41] | 17.2 [11.01-23.63] | 17.74 [11.4-24.26] | 1.4 [-0.08 to 2.91] |
| Latvia | 9.6 [8.41-10.69] | 29.47 [25.99-32.77] | 12.06 [9.93-14.55] | 36.27 [30.05-43.66] | -0.28 [-2.11 to 1.58] |
| Turkey | 81.69 [59.91-103.87] | 16.74 [12.33-20.81] | 204.2 [140.25-261.58] | 24 [16.42-30.51] | 0.91 [-0.69 to 2.53] |
| Congo | 0.64 [0.46-0.87] | 3.68 [2.8-4.82] | 1.77 [1.09-2.51] | 4.5 [2.73-6.41] | 0.21 [-1.75 to 2.22] |
| Cyprus | 2.03 [1.53-2.88] | 25.33 [19.24-36.47] | 8.94 [5.55-11.64] | 50.93 [31.94-65.32] | 2.66 [0.44 to 4.93] |
| Yemen | 11 [6.19-17.33] | 10.51 [6.3-16.07] | 27.36 [14.83-40.44] | 11.45 [6.07-17.07] | 0.13 [-1.13 to 1.41] |
| Barbados | 0.41 [0.38-0.43] | 15.35 [14.34-16.35] | 0.69 [0.54-0.86] | 16.6 [12.99-21.24] | 0.75 [-0.83 to 2.36] |
| Sweden | 53.17 [48.47-57.93] | 50.81 [45.29-56.94] | 66.77 [57.79-75.94] | 42.07 [37.26-47.48] | 0.08 [-1.87 to 2.07] |
| United Republic of Tanzania | 12.18 [9.35-17.79] | 5.23 [4.18-7.14] | 30.35 [17.79-45.98] | 6.48 [3.97-9.49] | 0.51 [-1.26 to 2.31] |
| Algeria | 21.61 [15.44-28.75] | 9.68 [7.23-12.07] | 41.66 [29.31-56.72] | 10.28 [7.25-13.84] | 0.82 [-1.17 to 2.86] |
| Gambia | 0.09 [0.06-0.12] | 1.11 [0.73-1.44] | 0.24 [0.15-0.34] | 1.38 [0.87-1.83] | 0.31 [-1.71 to 2.36] |
| Antigua and Barbuda | 0.05 [0.04-0.05] | 8.23 [7.5-9.02] | 0.09 [0.09-0.1] | 10.03 [9.07-11.28] | 0.51 [-0.7 to 1.74] |
| Equatorial Guinea | 0.11 [0.08-0.15] | 3.34 [2.4-4.34] | 0.45 [0.22-0.74] | 4.98 [2.48-8.18] | 0.57 [-1.47 to 2.65] |
| Brazil | 101.93 [98.01-106.3] | 8.26 [7.94-8.56] | 238.97 [225.5-252.54] | 10.22 [9.6-10.87] | 0.32 [-0.95 to 1.61] |
| Colombia | 30.97 [28.82-33.17] | 11.26 [10.51-11.96] | 75.85 [63.01-91.58] | 15.83 [13.03-19.47] | 1.04 [-0.41 to 2.5] |
| Cuba | 14.74 [13.71-15.75] | 14.33 [13.27-15.4] | 24.63 [21.17-28.61] | 16.73 [14.26-19.53] | 0.4 [-0.95 to 1.77] |
| Pakistan | 75.32 [49.86-102.63] | 7.87 [5.52-10.13] | 167.07 [121.14-227.58] | 8.36 [6.22-11.32] | 0.08 [-1.98 to 2.18] |
| Israel | 19.37 [17.31-21.66] | 38.83 [34.69-43.35] | 52.68 [46.51-59.52] | 46.2 [41.06-52.09] | 0.71 [-1.24 to 2.7] |
| Morocco | 4.89 [3.6-7.37] | 2.28 [1.68-3.28] | 9.81 [6.5-13.04] | 2.77 [1.83-3.66] | 0.48 [-0.94 to 1.93] |
| Malawi | 1.75 [1.2-2.31] | 1.75 [1.27-2.18] | 2.81 [1.44-4.81] | 1.93 [1.15-2.92] | 0.09 [-1.66 to 1.86] |
| Romania | 32.19 [30.2-33.89] | 12.68 [11.88-13.45] | 66.54 [57.12-75.51] | 22.21 [19-25.57] | 1.46 [-0.31 to 3.26] |
| Venezuela (Bolivarian Republic of) | 13.99 [13.25-14.83] | 8.49 [8.06-8.94] | 32.05 [23.11-43.68] | 11.89 [8.52-16.29] | 0.63 [-0.63 to 1.91] |
| Kenya | 6.37 [4.53-8.54] | 3.92 [2.68-6] | 17.58 [12.29-24.52] | 5.2 [3.65-7.24] | 1.1 [-0.66 to 2.9] |
| Bhutan | 0.38 [0.2-0.57] | 7.59 [4.63-10.82] | 0.49 [0.31-0.77] | 7.33 [4.73-11.16] | 0.07 [-2.04 to 2.23] |
| Bahamas | 0.2 [0.18-0.22] | 9.94 [8.98-10.9] | 0.47 [0.37-0.58] | 11.38 [8.94-14.31] | 0.39 [-0.95 to 1.75] |
| Singapore | 3.07 [2.8-3.41] | 12.19 [10.96-13.9] | 10.5 [9.52-11.55] | 22 [19.27-25.21] | 1.42 [-0.29 to 3.16] |
| Poland | 79.71 [72.05-85.9] | 18.83 [16.95-20.33] | 208.06 [187.85-231.6] | 32.43 [29.28-35.88] | 0.55 [-1.21 to 2.33] |
| Kuwait | 2.87 [2.44-3.36] | 22.25 [19.44-25.13] | 6.75 [5.37-8.35] | 21.02 [16.56-26.43] | 0.06 [-1.78 to 1.93] |
| Puerto Rico | 5.5 [5.08-6] | 15.37 [14.18-16.75] | 9.8 [7.94-11.73] | 20.12 [16.59-23.91] | 0.91 [-0.47 to 2.31] |
| Italy | 391.53 [368.95-413.69] | 62.08 [56.98-68.8] | 526.04 [473.11-571.32] | 56.82 [52.34-62.05] | -0.72 [-2.55 to 1.15] |
| United Arab Emirates | 1.71 [1.19-2.31] | 19.08 [13.03-25.84] | 10.3 [6.81-15.01] | 19.04 [13.37-25.76] | 0.58 [-1.15 to 2.34] |
| Germany | 440.42 [402.54-474.4] | 43.62 [40.06-47.12] | 807.01 [720.69-886.65] | 56.84 [51.98-62.36] | 0.42 [-1.19 to 2.06] |
| Palau | 0.01 [0.01-0.01] | 6.29 [4.38-8.6] | 0.01 [0.01-0.02] | 7.01 [5.13-8.96] | 0.38 [-0.9 to 1.68] |
| Canada | 168.01 [157.11-180.27] | 57.57 [53-62.69] | 261.74 [237.17-287.46] | 46.32 [42.07-50.77] | -0.2 [-2.18 to 1.83] |
| Argentina | 42.38 [39.77-45.27] | 12.89 [12.1-13.74] | 68.94 [62.41-76.14] | 14.26 [12.75-15.95] | -0.06 [-1.35 to 1.24] |
| Ecuador | 8.27 [7.61-8.97] | 10.01 [9.26-10.81] | 24.04 [19.56-29.11] | 13.8 [11.22-16.7] | 1.54 [-0.1 to 3.2] |
| Ireland | 14.81 [13.65-16.08] | 38.26 [34.88-42.01] | 26.61 [23.87-29.76] | 40.59 [36.46-45.45] | 0.62 [-1.2 to 2.47] |
| Greece | 68.37 [63.74-73.31] | 57.63 [52.24-63.64] | 87.49 [79.4-94.09] | 54.33 [49.52-59.1] | -0.41 [-1.96 to 1.18] |
| North Macedonia | 2.26 [1.75-2.89] | 11.82 [9.23-15.13] | 6.83 [4.27-9.65] | 21.5 [13.42-30.19] | 1.18 [-6.64 to 9.65] |
| Zambia | 3.57 [2.5-5.31] | 5.11 [3.89-7.19] | 9.37 [5.71-13.61] | 6.72 [4.24-9.35] | 0.4 [-1.38 to 2.22] |
| Norway | 14.86 [13.98-15.73] | 28.71 [26.57-30.95] | 30.51 [27.33-33.6] | 39.24 [35.56-43] | 0.65 [-1.15 to 2.49] |
| Guam | 0.08 [0.06-0.1] | 6.46 [4.8-8.24] | 0.1 [0.08-0.12] | 5.83 [4.51-7.63] | -1.93 [-3.96 to 0.14] |
| Somalia | 2.87 [1.63-4.84] | 4.97 [3.22-7.97] | 8.23 [5.31-12.01] | 6.1 [4.08-8.7] | 0.65 [-1 to 2.33] |
| El Salvador | 5.42 [4.63-6.91] | 11.8 [9.89-14.87] | 11.19 [7.93-14.3] | 17.82 [12.64-22.75] | 1.53 [-0.38 to 3.47] |
| Ghana | 5.98 [2.96-8.14] | 4.63 [2.33-6.19] | 9.67 [6.43-13.91] | 3.43 [2.38-4.89] | -1.93 [-4.02 to 0.21] |
| Seychelles | 0.11 [0.09-0.15] | 17.62 [14.64-24.37] | 0.21 [0.17-0.27] | 17.77 [14.51-23.01] | 0.3 [-1.3 to 1.92] |
| Mozambique | 10.23 [7.18-14.31] | 7.39 [5.67-10.01] | 20.74 [11.91-36.47] | 8.46 [5.73-12.03] | 0.57 [-1.23 to 2.4] |
| Estonia | 7.17 [6.49-7.91] | 37.79 [34.32-41.77] | 9.83 [7.97-11.75] | 43.07 [35.27-51.28] | 0.2 [-1.62 to 2.06] |
| Central African Republic | 0.76 [0.51-1.07] | 3.62 [2.64-4.64] | 1.49 [0.96-2.12] | 3.79 [2.37-5.29] | 0.11 [-1.64 to 1.89] |
| Libya | 4.43 [3.37-5.81] | 13.94 [10.06-18.64] | 12.45 [8.64-17.05] | 21.88 [15.07-30.37] | 1.74 [0.15 to 3.36] |
| Trinidad and Tobago | 1.03 [0.96-1.1] | 9.84 [9.24-10.45] | 1.64 [1.25-2.07] | 10.2 [7.82-12.95] | 0.23 [-0.98 to 1.45] |
| Nigeria | 17.17 [11.98-23.13] | 2.08 [1.4-2.81] | 45.62 [22.91-64.75] | 2.34 [1.21-3.24] | 0.29 [-1.79 to 2.41] |
| Nicaragua | 3.14 [2.58-4.04] | 8.21 [6.8-10.22] | 5.9 [4.26-7.46] | 9.81 [7.17-12.3] | 0.83 [-0.58 to 2.27] |
| Sierra Leone | 0.78 [0.56-1.09] | 1.85 [1.31-2.44] | 2.05 [0.93-2.97] | 2.85 [1.22-4.1] | 1.57 [-0.33 to 3.5] |
| South Sudan | 2.43 [1.49-3.96] | 4.79 [3.29-7.43] | 5.52 [3.45-8.11] | 7.17 [4.68-10.01] | 1.21 [-0.6 to 3.06] |
| Uruguay | 6.27 [5.79-6.78] | 17.52 [16.26-18.91] | 11.27 [10.08-12.49] | 24.57 [21.98-27.28] | 0.51 [-0.95 to 1.99] |
| United States Virgin Islands | 0.12 [0.09-0.15] | 12.67 [9.35-15.97] | 0.17 [0.11-0.23] | 12.86 [8.68-17.56] | 0.04 [-1.84 to 1.96] |
| Mexico | 73.55 [70.56-77.06] | 9.59 [9.32-9.93] | 153.59 [138.25-171.11] | 12.33 [11.05-13.83] | 0.42 [-1.11 to 1.98] |
| Mali | 1.48 [0.97-1.96] | 1.71 [1.18-2.13] | 3.23 [1.82-5.16] | 1.69 [1.02-2.32] | -0.16 [-1.95 to 1.66] |
| Saint Kitts and Nevis | 0.05 [0.05-0.06] | 14.21 [12.99-15.51] | 0.09 [0.07-0.11] | 13.77 [11.36-16.57] | 0.66 [-1.26 to 2.61] |
| Croatia | 14.16 [12.43-15.88] | 24.37 [21.4-27.52] | 33.8 [28.64-39.56] | 44.42 [36.83-52.67] | 1.12 [-0.72 to 3] |
| Republic of Moldova | 6.23 [5.62-6.82] | 13.83 [12.43-15.22] | 5.66 [5.01-6.45] | 12.42 [10.65-14.76] | -0.3 [-2.04 to 1.47] |
| Honduras | 4.01 [3.26-4.91] | 9.77 [7.86-11.88] | 7.77 [5.59-11.14] | 9.47 [6.86-12.91] | 0.06 [-1.28 to 1.42] |
| Hungary | 29.83 [27.82-31.9] | 22.03 [20.55-23.69] | 56.96 [48.37-67.05] | 34.83 [29.44-41.98] | 0.55 [-1.13 to 2.27] |
| Spain | 191.73 [177.31-206.75] | 45.85 [41.3-51.36] | 319.08 [278.08-355.73] | 51.08 [45.03-57.09] | -0.02 [-1.82 to 1.81] |
| Guinea | 0.53 [0.36-0.71] | 0.81 [0.56-1.03] | 0.97 [0.51-1.63] | 0.85 [0.48-1.29] | 0.12 [-1.79 to 2.07] |
| Northern Mariana Islands | 0.03 [0.02-0.04] | 9.29 [5.18-12.47] | 0.06 [0.03-0.07] | 11.23 [5.74-14.3] | 0.05 [-3.89 to 4.16] |
| Austria | 38.02 [35.34-40.86] | 40.12 [36.79-43.9] | 65.75 [56.04-74.37] | 46.05 [40.19-51.53] | 0.48 [-1.23 to 2.23] |
| Belgium | 54.4 [49.3-59.52] | 46.36 [41.18-52.02] | 74.94 [64.78-85.66] | 46.05 [40.22-52.52] | -0.18 [-1.78 to 1.43] |
| Czechia | 30.69 [28.22-33.17] | 24.6 [22.66-26.9] | 79.5 [66-93.47] | 41.44 [34.19-49.85] | 0.81 [-1.07 to 2.72] |
| Chad | 0.92 [0.66-1.23] | 1.63 [1.21-2.09] | 3.92 [1.95-5.69] | 2.8 [1.32-4.04] | 1.91 [0.01 to 3.85] |
| Costa Rica | 3.51 [3.21-3.93] | 13.59 [12.49-14.81] | 10.97 [9.49-12.55] | 23.15 [20-26.82] | 1.29 [-0.04 to 2.64] |
| Peru | 21.14 [17.28-27.18] | 11.15 [9.15-14.08] | 62.11 [39.05-82.73] | 17.85 [11.2-23.79] | 2.08 [0.27 to 3.91] |
| Monaco | 0.38 [0.26-0.54] | 111.04 [74.19-159.41] | 0.73 [0.46-1.01] | 139.25 [94.43-189.55] | 1.01 [-0.88 to 2.94] |
| Netherlands | 73.12 [67.25-79.44] | 45.33 [41.12-50.63] | 96.09 [83.38-108.15] | 36.48 [32.15-40.75] | -0.09 [-1.78 to 1.63] |
| American Samoa | 0.03 [0.02-0.04] | 7.38 [5.7-10.02] | 0.05 [0.03-0.06] | 9.54 [6.65-12.4] | 0.49 [-2.45 to 3.52] |
| Gabon | 0.26 [0.19-0.33] | 3.41 [2.42-4.33] | 0.68 [0.39-1.02] | 4.96 [2.83-7.3] | 0.7 [-1.36 to 2.79] |
| Sudan | 20.28 [10.77-32.21] | 11.39 [7-16.9] | 47.18 [29.2-67.11] | 13.52 [8.37-19.22] | 0.39 [-0.98 to 1.78] |
| Jamaica | 2.58 [2.33-2.89] | 12.41 [11.27-13.72] | 4.18 [3.17-5.55] | 14.36 [10.9-19.06] | 1.04 [-0.88 to 2.99] |
| Australia | 71.83 [67.3-76.58] | 39.01 [36.42-41.76] | 154.7 [136.98-173.62] | 41.67 [37.2-46.76] | 0.04 [-1.64 to 1.76] |
| Brunei Darussalam | 0.25 [0.19-0.31] | 13.01 [10.27-16.52] | 0.54 [0.38-0.66] | 12.96 [8.97-15.98] | 0.32 [-1.24 to 1.9] |
| Luxembourg | 1.99 [1.84-2.14] | 44.12 [40.51-48.21] | 4 [3.55-4.54] | 46.71 [41.26-53.97] | -0.09 [-1.74 to 1.58] |
| Palestine | 4.11 [2.99-5.72] | 27.82 [20.39-36.69] | 11.19 [8.76-14.69] | 29.88 [24-38.59] | 0.26 [-1.92 to 2.48] |
| Iceland | 1.11 [1-1.23] | 42.74 [38.39-47.31] | 2.01 [1.75-2.29] | 45.05 [39.19-51.27] | 0.25 [-1.49 to 2.02] |
| Burkina Faso | 1.76 [1.26-2.28] | 2 [1.42-2.52] | 5.48 [2.53-7.61] | 2.91 [1.3-4.04] | 1.41 [-0.44 to 3.29] |
| Niger | 1.59 [1.08-2.17] | 1.97 [1.45-2.5] | 4.73 [1.99-7.65] | 2.49 [1.06-4.23] | 0.81 [-1.1 to 2.76] |
| Botswana | 0.4 [0.27-0.55] | 4.81 [3.11-6.52] | 1.06 [0.65-1.55] | 5.71 [3.52-8.19] | 0.36 [-2.29 to 3.09] |
| Montenegro | 1.37 [0.96-1.75] | 22.29 [15.54-28.71] | 2.65 [1.79-3.85] | 29.1 [19.53-41.4] | 0.9 [-1 to 2.84] |
| Ethiopia | 80.78 [39.29-125.29] | 21.19 [13.16-29.55] | 131.06 [86.36-204.85] | 17.42 [11.77-27.9] | -1.21 [-3.21 to 0.83] |
| Grenada | 0.11 [0.1-0.12] | 14.37 [12.69-16.03] | 0.19 [0.17-0.22] | 17.28 [14.8-19.67] | 0.6 [-1.02 to 2.25] |
| Saint Lucia | 0.12 [0.11-0.13] | 11.07 [10.34-11.9] | 0.25 [0.21-0.31] | 11.94 [9.71-14.76] | -0.09 [-1.42 to 1.25] |
| Japan | 286.53 [267.6-311.57] | 25.2 [22.68-28.74] | 444.83 [403.91-473.48] | 26.73 [24.69-28.89] | -0.29 [-1.92 to 1.37] |
| Serbia | 15.63 [11.9-19.11] | 14.85 [11.66-18.32] | 38.89 [27.94-52.21] | 26.15 [18.9-34.92] | 1.03 [-0.81 to 2.89] |
| Comoros | 0.19 [0.14-0.27] | 4.9 [3.59-6.7] | 0.41 [0.27-0.59] | 6.49 [4.4-9.16] | 0.45 [-1.39 to 2.33] |
| Lesotho | 0.43 [0.31-0.58] | 3.97 [2.83-5.36] | 1.01 [0.71-1.38] | 7.29 [5.2-9.93] | 2.5 [-0.1 to 5.17] |
| Iran (Islamic Republic of) | 79.27 [43.84-108.15] | 16.55 [9.18-21.85] | 179.13 [90.91-228.15] | 22.56 [11.51-29.13] | 1.05 [-0.41 to 2.53] |
| Togo | 0.58 [0.42-0.73] | 1.91 [1.35-2.36] | 2.01 [0.85-2.96] | 3.17 [1.35-4.62] | 1.61 [-0.4 to 3.65] |
| Zimbabwe | 3.16 [2.21-3.85] | 5.06 [3.61-6.43] | 8.25 [5.74-11.13] | 7.62 [5.37-10.16] | 2.04 [-0.7 to 4.86] |
| Eswatini | 0.25 [0.19-0.34] | 5.29 [4.01-6.8] | 0.59 [0.38-0.89] | 7.33 [4.79-10.99] | 1.02 [-1.54 to 3.65] |
| Eritrea | 1.21 [0.8-1.89] | 4.58 [3.26-6.76] | 3.05 [1.88-4.29] | 6.25 [4.03-8.41] | 0.79 [-0.87 to 2.49] |
| Guinea-Bissau | 0.21 [0.14-0.3] | 2.41 [1.67-3.38] | 0.49 [0.26-0.69] | 3.33 [1.72-4.69] | 1.17 [-0.68 to 3.06] |
| Guatemala | 5.52 [5.15-5.94] | 7.75 [7.34-8.16] | 14.45 [12.43-16.93] | 10.17 [8.71-11.91] | 0.5 [-1.32 to 2.37] |
| Angola | 2.71 [1.73-3.94] | 3.31 [2.37-4.38] | 8.53 [5.25-12.07] | 3.94 [2.2-5.99] | 0.35 [-1.53 to 2.26] |
| Lithuania | 14.82 [13.5-16.2] | 35.23 [32.07-38.52] | 14.93 [12.34-17.44] | 31.61 [26.63-37.06] | -0.54 [-2.27 to 1.21] |
| Switzerland | 46.28 [42.1-50.99] | 62.33 [56.22-69.59] | 65.69 [56.29-76.59] | 51.38 [44.61-59.18] | -0.52 [-2.23 to 1.22] |
| Cabo Verde | 0.17 [0.13-0.23] | 5.41 [4.37-7.35] | 0.54 [0.35-0.72] | 10.75 [7.13-14.39] | 1.51 [-0.72 to 3.79] |
| New Zealand | 16.49 [15.19-17.76] | 43.55 [40.11-47.03] | 38.37 [34.15-42.23] | 52.14 [47.1-57.26] | 0.37 [-1.28 to 2.04] |
| Rwanda | 3.49 [2.34-5.25] | 5.76 [4.1-8.27] | 6.29 [3.8-9.69] | 6.32 [3.98-9.33] | -0.46 [-2.1 to 1.21] |
| Senegal | 1.45 [1.1-1.84] | 2.07 [1.47-2.55] | 3.83 [1.72-6] | 3.13 [1.43-4.75] | 1.36 [-0.73 to 3.5] |
| Bermuda | 0.1 [0.09-0.11] | 16.55 [14.94-18.36] | 0.21 [0.17-0.25] | 22.92 [18.05-28.97] | 1.32 [-0.08 to 2.73] |
| Paraguay | 2.84 [2.27-3.46] | 8.1 [6.37-9.84] | 8.46 [5.97-11.38] | 13.26 [9.43-17.89] | 1.46 [0.17 to 2.75] |
| Saint Vincent and the Grenadines | 0.14 [0.13-0.16] | 15.83 [14.46-17.16] | 0.2 [0.17-0.23] | 15.3 [13.2-17.73] | 0 [-1.62 to 1.64] |
| Sao Tome and Principe | 0.02 [0.01-0.02] | 1.52 [1.05-1.91] | 0.03 [0.02-0.05] | 1.92 [1.2-2.73] | 0.3 [-1.62 to 2.26] |
| Saudi Arabia | 7.64 [5.37-12.1] | 6.6 [4.57-11.23] | 47.18 [33.03-75.66] | 16.39 [11.85-25.3] | 3.13 [1.82 to 4.47] |
| Lebanon | 3.32 [2.21-5.38] | 13 [8.64-21.18] | 13.35 [9.01-18.02] | 23.79 [15.99-32.52] | 1.86 [0.36 to 3.38] |
| Madagascar | 4.28 [3.16-6.43] | 4.15 [3.27-5.79] | 10.4 [6.37-14.76] | 4.85 [3.09-6.67] | 0.37 [-1.39 to 2.16] |
| Qatar | 0.47 [0.29-0.61] | 21.17 [13.36-28.01] | 4.28 [2.44-6.21] | 27.33 [16.35-38.51] | 0.86 [-0.94 to 2.68] |
| Cook Islands | 0 [0-0.01] | 3.09 [2.13-4.28] | 0.01 [0-0.01] | 2.97 [1.92-4.26] | -1.72 [-3.62 to 0.23] |
| Egypt | 39.13 [30.64-66.5] | 8.47 [6.57-14.64] | 147.5 [89.06-198.78] | 17.29 [10.73-22.94] | 2.7 [1.28 to 4.15] |
| Albania | 2.36 [1.82-3] | 8.63 [6.5-11.3] | 5.26 [3.14-9.41] | 16.2 [9.8-26.55] | 2.06 [0.25 to 3.91] |
| Belize | 0.15 [0.13-0.17] | 9.18 [8.45-10.12] | 0.39 [0.34-0.45] | 10.76 [9.46-12.26] | 0.75 [-0.99 to 2.52] |
| Nauru | 0.01 [0-0.01] | 8.5 [6.03-11.94] | 0.01 [0-0.01] | 8.34 [5.45-12.33] | -1.68 [-4.14 to 0.83] |
| Guyana | 0.36 [0.32-0.41] | 6.1 [5.43-6.8] | 0.51 [0.39-0.67] | 7.09 [5.37-9.22] | 0.81 [-0.46 to 2.1] |
| Tunisia | 6.02 [4.48-7.68] | 8.21 [5.93-10.35] | 14.37 [8.28-21.26] | 11.73 [6.81-17.46] | 0.8 [-0.74 to 2.36] |
| Bolivia (Plurinational State of) | 8.17 [5.59-11.13] | 14.9 [10.36-20.07] | 15.48 [10.58-20.49] | 14.34 [9.86-19.08] | 0.02 [-1.6 to 1.67] |
| United States of America | 1606.95 [1542.79-1649.3] | 55.42 [53.52-56.82] | 1928.57 [1786.61-2010.32] | 39.75 [37.34-41.42] | -0.83 [-2.57 to 0.95] |
| Uganda | 6.41 [4.26-8.69] | 3.96 [2.9-5.15] | 17.71 [8.96-30.49] | 5.05 [3.03-7.44] | 0.52 [-1.33 to 2.41] |
| Russian Federation | 294.46 [279.74-309.64] | 17.61 [16.73-18.48] | 522.67 [479.07-567.03] | 25.77 [23.77-27.87] | 0.72 [-0.92 to 2.38] |
| Burundi | 2.4 [1.64-3.45] | 4.99 [3.58-7.11] | 5.1 [2.73-8.09] | 5.41 [3.11-8.33] | 0.05 [-1.59 to 1.71] |
| Dominican Republic | 5.77 [4.41-7.18] | 8.86 [6.71-10.89] | 9.64 [7.15-12.69] | 9.1 [6.74-12] | 0.53 [-1.18 to 2.26] |
| Slovakia | 12.1 [8.89-15.4] | 20.9 [15.48-26.62] | 28.77 [19.29-38.48] | 33.64 [22.64-44.32] | 0.9 [-1.26 to 3.1] |
| Syrian Arab Republic | 15.68 [11.44-21.88] | 16.28 [11.5-21.43] | 31.31 [19.61-44.84] | 22.97 [14.8-32.38] | 0.58 [-0.87 to 2.06] |
| Mauritius | 0.93 [0.86-1.01] | 10.33 [9.57-11.14] | 2.01 [1.87-2.14] | 12.98 [12.03-13.9] | 0.7 [-1.39 to 2.84] |
| Cameroon | 1.97 [1.45-2.58] | 2.2 [1.65-2.83] | 8.12 [3.74-12.13] | 3.34 [1.48-5.1] | 1.35 [-0.66 to 3.4] |
| Chile | 12.61 [11.85-13.43] | 10.59 [9.97-11.26] | 33.63 [30.18-37.41] | 16.75 [14.74-19.07] | 1.44 [0.12 to 2.77] |
| South Africa | 17.56 [12.91-20.73] | 6.26 [4.2-7.8] | 43.79 [27.42-52.08] | 8.57 [5.28-10.24] | 0.36 [-2.26 to 3.05] |
| Nepal | 12.65 [7.48-18.34] | 7.45 [4.95-10.29] | 18.79 [13.74-26.45] | 6.84 [4.99-9.66] | 0.15 [-1.88 to 2.23] |
| Mauritania | 0.33 [0.25-0.42] | 1.92 [1.5-2.38] | 1.13 [0.53-1.73] | 3.38 [1.53-4.91] | 1.35 [-0.83 to 3.58] |
| Benin | 0.91 [0.68-1.17] | 1.97 [1.51-2.43] | 3.22 [1.48-4.68] | 2.93 [1.33-4.12] | 1.25 [-0.77 to 3.3] |
| Denmark | 28.66 [26.45-31.07] | 43.18 [39.62-46.86] | 50.69 [44.12-57.56] | 53.94 [47.51-60.53] | 0.5 [-1.27 to 2.3] |
| Panama | 2.02 [1.82-2.23] | 9.23 [8.43-10.03] | 7.05 [5.73-8.74] | 16.93 [13.74-21.19] | 1.37 [-0.08 to 2.85] |
| Belarus | 33.38 [30.69-36.25] | 28.14 [25.8-30.73] | 52.71 [41.73-66.37] | 36.99 [29.17-46.08] | 0.59 [-1.33 to 2.55] |
| Oman | 1.44 [0.96-2.07] | 11.18 [7.01-15.53] | 3.98 [2.73-5.5] | 13.59 [9.1-18.87] | 0.8 [-1.14 to 2.79] |
| Niue | 0 [0-0] | 6.12 [4.05-8.52] | 0 [0-0] | 12.27 [8.91-15.83] | -0.71 [-3.03 to 1.66] |
| Bangladesh | 82.95 [50.89-124.81] | 8.68 [5.98-11.69] | 109.46 [74.47-156.72] | 7.17 [4.9-10.21] | -0.32 [-2.28 to 1.69] |
| Côte d'Ivoire | 1.56 [1.04-1.99] | 1.64 [1.02-2.06] | 4.16 [2.23-6.34] | 1.95 [1.04-2.85] | 0.5 [-1.53 to 2.57] |
| Portugal | 27.59 [25.56-29.95] | 24.51 [22.23-27.34] | 61.06 [52.81-68.73] | 38.56 [33.69-44.27] | 1.16 [-0.5 to 2.86] |
| Uzbekistan | 22.45 [20.14-25.21] | 12.51 [11.21-14.05] | 33.32 [26.73-40.93] | 9.74 [7.84-11.99] | -0.85 [-2.5 to 0.83] |
| Dominica | 0.06 [0.05-0.08] | 9.78 [7.66-11.64] | 0.1 [0.07-0.12] | 13.67 [9.71-17.69] | 0.99 [-0.27 to 2.26] |
| Tokelau | 0 [0-0] | 6.24 [3.89-8.85] | 0 [0-0] | 13.71 [8.08-22.05] | -0.94 [-3.33 to 1.51] |
| Malta | 0.93 [0.84-1.05] | 24.57 [21.74-27.93] | 2.08 [1.73-2.41] | 38.16 [31.52-45.48] | 0.93 [-0.67 to 2.56] |
| Bahrain | 0.57 [0.41-0.7] | 19.04 [13.57-23.63] | 2.05 [1.44-2.71] | 17.76 [12.86-23.67] | -0.12 [-2.22 to 2.03] |
| San Marino | 0.3 [0.24-0.41] | 137.73 [100.66-189.9] | 0.34 [0.21-0.51] | 84.77 [52.59-121.66] | -0.54 [-2.82 to 1.79] |
| Tuvalu | 0.01 [0-0.01] | 6.8 [4.58-9.62] | 0.01 [0.01-0.01] | 6.28 [4.44-8.39] | -1.69 [-4.13 to 0.8] |
| Bosnia and Herzegovina | 4.07 [3.17-5.14] | 9.42 [7.51-11.95] | 10.88 [6.96-15.25] | 19.52 [12.79-27.32] | 2.15 [0.3 to 4.05] |
| Finland | 17.57 [16.36-18.87] | 28.45 [26.43-30.69] | 34.9 [30.75-39.16] | 39.13 [35.15-43.61] | 0.92 [-0.8 to 2.67] |
| Ukraine | 152.89 [134.72-168.89] | 25.85 [22.75-28.86] | 128.3 [94.01-170.17] | 21.08 [16.03-27.38] | -1.05 [-2.56 to 0.49] |
| Haiti | 7.67 [3.86-12.16] | 13.57 [8.03-20.4] | 13.06 [7.79-19.55] | 11.88 [7.48-17.27] | -0.26 [-1.62 to 1.12] |
| Iraq | 17.15 [11.77-28.09] | 11.52 [8.27-17.91] | 59.39 [41.68-79.39] | 17.62 [12.34-23.3] | 1.42 [0.45 to 2.39] |
| Namibia | 0.35 [0.27-0.43] | 3.59 [2.79-4.43] | 0.91 [0.6-1.3] | 4.92 [3.4-6.83] | 0.44 [-2.21 to 3.17] |
| Democratic Republic of the Congo | 8.8 [6.44-11.82] | 3.01 [2.1-3.92] | 21.61 [13.35-31.13] | 3.65 [2.03-5.54] | 0.51 [-1.4 to 2.46] |
| Bulgaria | 14.42 [13.19-15.74] | 13.85 [12.66-15.18] | 19.73 [15.48-24.64] | 17.44 [13.81-21.7] | 0.62 [-0.93 to 2.19] |
| Greenland | 0.03 [0.03-0.05] | 7.62 [5.9-10.57] | 0.05 [0.04-0.07] | 7.67 [5.52-10.59] | 0.17 [-1.27 to 1.63] |
| India | 478.94 [366.38-603.42] | 6.69 [5.04-8.32] | 850.15 [660.89-1042.74] | 6.54 [5.07-8.02] | 0.12 [-1.66 to 1.94] |
| Andorra | 0.32 [0.21-0.52] | 68.47 [42.81-109.63] | 0.65 [0.41-1.02] | 58.91 [37.41-89.89] | -0.27 [-2.19 to 1.69] |
| Slovenia | 6.49 [5.78-7.18] | 28.24 [25.2-31.18] | 23.93 [19.38-29.68] | 61.08 [49.57-74.44] | 1.94 [-0.06 to 3.97] |
| Republic of Korea | 39.21 [31.03-50.6] | 9.85 [7.91-13.01] | 90.45 [62.96-117.9] | 20.16 [12.07-28.03] | 1.9 [0.35 to 3.47] |
| Suriname | 0.29 [0.2-0.33] | 8.61 [5.97-10.07] | 0.56 [0.41-0.73] | 9.33 [6.89-12.1] | 0.31 [-1.11 to 1.76] |
| Liberia | 0.46 [0.31-0.68] | 1.93 [1.45-2.6] | 1.3 [0.57-1.99] | 3.2 [1.38-4.83] | 1.68 [-0.28 to 3.68] |
| Djibouti | 0.14 [0.09-0.22] | 4.43 [2.96-6.5] | 0.61 [0.34-1.02] | 6.47 [3.75-10.47] | 0.93 [-0.89 to 2.8] |

| **Country** | **1990** | | **2021** | | **1990-2021** |
| --- | --- | --- | --- | --- | --- |
|  | **DALYs cases** | **ASDR per 100,000** | **DALYs cases** | **ASDR per 100,000** | **EAPC** |
|  | **No. *10^2^ (95% UI)** | **No. (95% UI)** | **No. *10^2^ (95% UI)** | **No.(95% UI)** | **No. (95% CI)** |
| Republic of Korea | 892.26 [691.36-1054.32] | 207.43 [165.7-250.41] | 535.04 [343.5-649.12] | 81.98 [53.19-99.63] | -3.46 [-5.46 to -1.42] |
| American Samoa | 0.57 [0.45-0.71] | 135.05 [106.86-165.93] | 0.66 [0.49-0.83] | 133.94 [100.89-169.23] | 0.29 [-2.97 to 3.66] |
| Cambodia | 317.24 [183.06-489.62] | 308.92 [198.34-446.74] | 370.61 [262.51-498.61] | 230.16 [165-305.83] | -1.1 [-2.92 to 0.76] |
| Fiji | 15.44 [8.58-20.72] | 223.26 [124.9-297.53] | 17.53 [9.68-24.48] | 197.02 [109.47-272.81] | -1.67 [-5.04 to 1.82] |
| Niue | 0.03 [0.02-0.05] | 148.01 [99.91-198.38] | 0.04 [0.03-0.05] | 277.47 [205-343.33] | -1.5 [-4.49 to 1.58] |
| Benin | 34.73 [24.92-48.15] | 53.97 [41.55-70.2] | 89.3 [41.62-131.62] | 58.69 [28.27-84.1] | 0.59 [-1.31 to 2.53] |
| Croatia | 92.13 [79.5-106.17] | 166.91 [143.81-193.37] | 90.68 [76.29-107.29] | 128.69 [106.97-154.7] | -1.71 [-3.01 to -0.4] |
| Guyana | 10.6 [8.92-11.99] | 144.6 [125.23-161.69] | 10.04 [7.67-12.91] | 134.45 [103.32-172.61] | 0.24 [-1.23 to 1.73] |
| United Arab Emirates | 31.26 [22.44-41.31] | 274.13 [192.76-361.42] | 96.15 [66.74-130.47] | 155.72 [119.21-202.22] | -1.05 [-2.36 to 0.28] |
| Azerbaijan | 180.55 [146.11-217.95] | 239.66 [195.06-293.16] | 161.36 [112.96-228.19] | 161.58 [113.84-224.7] | -1.71 [-3.48 to 0.09] |
| Estonia | 45.66 [42.96-48.52] | 270.11 [253.15-287.87] | 26.97 [22.94-30.58] | 135.97 [116.98-153.28] | -2.95 [-4.24 to -1.64] |
| Colombia | 763.49 [714.41-813.38] | 235.61 [222.66-249.16] | 864.26 [725.64-1019.11] | 175.72 [147.53-207.64] | -1.37 [-3.1 to 0.4] |
| Canada | 548.01 [525.12-568.4] | 183.69 [176.38-190.54] | 665.69 [611.09-715.12] | 113.08 [104.94-121.64] | -1.82 [-3.32 to -0.3] |
| Hungary | 276.47 [257.85-297.46] | 227.41 [211.59-245.87] | 213.93 [178.97-256.09] | 143.92 [119.25-173.61] | -2.03 [-3.3 to -0.75] |
| Nigeria | 619.01 [413.55-895.88] | 55.51 [39.39-78.28] | 1319.24 [689.99-1835.9] | 49.23 [27.13-66.53] | -0.4 [-2.32 to 1.56] |
| Lao People's Democratic Republic | 132.57 [65.19-215.05] | 314.72 [172.66-480.72] | 145.65 [102.65-201.93] | 209.05 [149.56-283.05] | -1.56 [-3.45 to 0.37] |
| Marshall Islands | 0.59 [0.42-0.76] | 157.14 [112.37-205.12] | 0.85 [0.55-1.2] | 162.08 [105.93-226.71] | -1.62 [-4.54 to 1.39] |
| Jamaica | 33.82 [30.88-37.15] | 142.78 [131.59-155.79] | 37.38 [28.64-48.58] | 131.11 [100.75-169.38] | 0 [-1.66 to 1.68] |
| Ireland | 68.48 [65.15-72.03] | 176.48 [168.22-185.61] | 61.04 [55.78-66.77] | 90.38 [83.35-98.48] | -2.57 [-4.07 to -1.04] |
| New Zealand | 69.03 [66.11-71.73] | 189.76 [182.07-197.66] | 83.31 [77.86-88.08] | 119 [112.31-125.07] | -1.46 [-3.06 to 0.17] |
| Kazakhstan | 323.37 [304.33-343.3] | 199.88 [188.37-212.09] | 201.21 [180.74-224.38] | 106 [95.32-118.08] | -1.79 [-3.32 to -0.24] |
| Lithuania | 95.49 [89.6-101.14] | 245.07 [230.31-260.71] | 64.94 [56.26-73.16] | 157.12 [137.07-175.66] | -1.8 [-3.04 to -0.55] |
| El Salvador | 141.11 [119.92-178.83] | 247.29 [213.95-311.15] | 135.79 [98.04-168.29] | 211.09 [152.18-261.8] | -0.37 [-2.29 to 1.58] |
| Kenya | 153.79 [109.63-197.43] | 68.87 [50.3-93.64] | 304.79 [212.85-413.76] | 72.92 [52.15-99.27] | 0.66 [-0.71 to 2.05] |
| Botswana | 10.93 [7.64-15.16] | 104.35 [72.77-143.91] | 19.78 [13.17-28.39] | 96.68 [66.46-136.02] | -0.31 [-2.98 to 2.44] |
| Jordan | 91.5 [73.98-114.97] | 289.75 [231.63-357.92] | 184.08 [132.77-241.93] | 173.84 [125.65-228.09] | -1.7 [-3.6 to 0.24] |
| Côte d'Ivoire | 52.71 [35.73-69.58] | 38.3 [26.36-48.52] | 99.05 [54.02-156.33] | 34.99 [19.78-52.33] | -0.16 [-1.97 to 1.69] |
| Angola | 103.06 [56.91-160.57] | 95.39 [65.21-131.43] | 224.94 [142.2-319.64] | 77.34 [47.65-115.58] | -0.46 [-2.53 to 1.66] |
| Panama | 46.2 [42.69-50.21] | 189.61 [176.35-204.1] | 81.39 [67.69-96.43] | 190.24 [158.91-225.54] | -0.27 [-2.09 to 1.58] |
| Belize | 2.79 [2.57-3.05] | 135.43 [126.88-146.33] | 4.29 [3.85-4.8] | 107.63 [96.51-120.18] | -0.28 [-1.59 to 1.05] |
| Russian Federation | 3017.14 [2962.38-3082.42] | 195.06 [191.45-199.51] | 2263.26 [2105.77-2432.18] | 119.54 [111.82-128.02] | -1.81 [-3.09 to -0.52] |
| Myanmar | 1352 [688.19-2122.45] | 334.83 [186.53-505.96] | 1130.56 [835.48-1502.18] | 206.05 [153.23-274.94] | -2.07 [-4.04 to -0.07] |
| Andorra | 1.25 [0.85-1.88] | 231.2 [156.09-342.69] | 1.54 [1-2.17] | 127.18 [83.64-175.38] | -1.91 [-3.46 to -0.33] |
| Italy | 1515.57 [1467.36-1554.18] | 224.69 [218.86-229.96] | 1376.02 [1256.22-1459.96] | 128.5 [120.97-135.84] | -2.61 [-4 to -1.19] |
| Yemen | 331.53 [184.39-554.17] | 248.19 [152.95-390.06] | 565.83 [308.6-814.29] | 202.29 [112.01-296.09] | -0.81 [-2.27 to 0.66] |
| Samoa | 2.83 [2.19-3.84] | 196.48 [153.77-263.5] | 3.24 [2.41-4.5] | 171.84 [130.77-236.76] | -2 [-4.73 to 0.82] |
| Dominica | 1.37 [1.15-1.68] | 195.36 [164.84-237.48] | 1.51 [1.12-1.91] | 225.74 [166.03-285.31] | 0.33 [-1.2 to 1.9] |
| Central African Republic | 27.82 [17.23-42.54] | 102.96 [73.11-140.95] | 46.38 [29.13-69.49] | 93.19 [58.7-131.69] | -0.18 [-2.13 to 1.81] |
| Madagascar | 125.15 [93.01-169.17] | 93.56 [73.06-121.42] | 225.96 [146.31-314.06] | 83.08 [54.79-113.88] | -0.15 [-1.66 to 1.39] |
| Lebanon | 57.89 [38.93-87.77] | 214.13 [145.42-322.28] | 85.1 [67.27-109.73] | 147.71 [116.57-189.82] | -1.14 [-2.61 to 0.35] |
| Congo | 20.37 [14.06-28.79] | 92.71 [69.29-123.94] | 36.91 [23.87-51.54] | 79.27 [50.57-111.05] | -0.59 [-2.65 to 1.52] |
| Lesotho | 11.29 [8.05-15.48] | 86.2 [61.83-116.17] | 23.22 [16.47-31.39] | 147.36 [105.32-199.28] | 2.53 [-0.09 to 5.22] |
| Tajikistan | 120.79 [90.16-149.95] | 200.27 [151.78-245.41] | 131.85 [83.66-209.9] | 125.78 [80.82-193.72] | -2.13 [-3.9 to -0.34] |
| Chile | 231.31 [222.28-240.32] | 183.2 [176.34-190.02] | 244.15 [228.31-260.25] | 116.23 [108.83-123.79] | -1.51 [-3.05 to 0.05] |
| Ecuador | 205.23 [192.64-218.75] | 205.16 [193.35-217.43] | 378.17 [313.18-449.69] | 212.19 [175.98-252.42] | 0.12 [-1.76 to 2.04] |
| Grenada | 1.66 [1.49-1.85] | 194.6 [175.06-216.9] | 1.66 [1.45-1.89] | 156.67 [137.01-176.34] | -0.47 [-1.51 to 0.58] |
| Gambia | 3.18 [2.1-4.41] | 26.94 [18.45-35.56] | 5.7 [3.59-8.62] | 24.09 [16.07-33.45] | -0.42 [-2.26 to 1.45] |
| Togo | 20.79 [14.92-27.2] | 48 [36.61-59.73] | 44.4 [19.91-66.39] | 55.01 [25.79-80.04] | 0.93 [-0.91 to 2.8] |
| Solomon Islands | 4.83 [2.53-7.07] | 162.73 [81.29-242.68] | 10.14 [6.16-13.95] | 163.93 [100.04-222.14] | -1.78 [-4.68 to 1.21] |
| Paraguay | 72.46 [57.4-87.38] | 176.01 [139.29-211.78] | 112.27 [81.86-144.55] | 166.34 [121.14-213.56] | 0.14 [-1.43 to 1.74] |
| Equatorial Guinea | 3.9 [2.46-5.83] | 91.85 [65.01-124.62] | 8.73 [4.26-14.6] | 70.36 [35.97-116.48] | -1.07 [-3.17 to 1.06] |
| Belgium | 247.73 [232.96-262.13] | 201.98 [191.03-212.63] | 237.03 [212.33-259.28] | 131.05 [120.91-142.19] | -2.21 [-3.59 to -0.82] |
| Bermuda | 1.18 [1.09-1.28] | 195.79 [180.88-212.52] | 0.96 [0.8-1.15] | 109.7 [91.47-132.33] | -2.08 [-3.59 to -0.55] |
| Mozambique | 315.35 [227.05-426.41] | 179.42 [137.16-237.07] | 453.18 [255.68-811.13] | 145.93 [93.49-218.7] | 0.09 [-1.45 to 1.66] |
| Philippines | 1448.71 [1166.86-1875.32] | 227.36 [187.19-277.55] | 1926.89 [1550.16-2261.61] | 176.64 [144.88-207.7] | -0.83 [-2.81 to 1.19] |
| Saint Kitts and Nevis | 0.81 [0.75-0.87] | 198.71 [186.31-213.15] | 0.72 [0.6-0.85] | 122.91 [103.34-145.2] | -1.06 [-2.37 to 0.26] |
| Turkmenistan | 71.4 [65.28-77.61] | 180.31 [166.45-194.37] | 70.61 [56.75-87.75] | 136.92 [110.18-170.1] | -1.2 [-2.88 to 0.51] |
| Vanuatu | 1.79 [1.1-2.5] | 137.75 [82.36-189] | 3.85 [2.51-5.09] | 136.07 [88.88-178.94] | -1.88 [-4.8 to 1.12] |
| Malawi | 56.85 [36.72-75.89] | 44.43 [29.79-56.49] | 60.68 [29.22-108.67] | 32.35 [17.59-53.39] | -0.73 [-2.27 to 0.84] |
| Namibia | 9.77 [7.08-12.43] | 80.51 [60.37-101.22] | 17.1 [11.34-25.09] | 80.61 [55.23-114.99] | -0.35 [-3 to 2.36] |
| Uzbekistan | 466.26 [421.16-517.79] | 217.26 [197.32-239.46] | 475.26 [391.17-578.45] | 138.64 [114.25-169] | -1.77 [-3.48 to -0.03] |
| Thailand | 1157.87 [892.23-1384.86] | 223.92 [170.56-269.14] | 1421.45 [845.76-1878.89] | 186.32 [115.21-241.88] | -1.47 [-3.2 to 0.28] |
| Ukraine | 1557.25 [1475.37-1641.64] | 292.22 [275.32-311.69] | 723.56 [536.93-934.99] | 133.35 [101.55-168.08] | -2.64 [-3.89 to -1.38] |
| Ghana | 192.75 [96.52-269.1] | 113.03 [58.67-154.51] | 200.47 [131.45-292.49] | 58.86 [39.78-85.62] | -2.59 [-4.5 to -0.65] |
| Cook Islands | 0.11 [0.08-0.14] | 62.37 [45.06-81.39] | 0.08 [0.06-0.1] | 40.35 [27.91-52.81] | -3.5 [-6.15 to -0.76] |
| Puerto Rico | 72.01 [68.18-76.27] | 199.38 [188.96-211.13] | 63.54 [52.47-74.56] | 135.61 [112.4-158.87] | -1.72 [-3.19 to -0.23] |
| Greenland | 0.65 [0.46-0.8] | 137.16 [102.02-166] | 0.46 [0.35-0.61] | 73.21 [54.92-96.2] | -1.86 [-3.37 to -0.34] |
| Sri Lanka | 345.98 [276.5-413.81] | 211.98 [172.9-254.55] | 270 [179.79-376.56] | 113.59 [76.58-157.53] | -1.25 [-3.64 to 1.21] |
| San Marino | 0.75 [0.62-1] | 270.5 [221.07-362.07] | 0.69 [0.45-0.97] | 135.92 [89.08-194.8] | -1.6 [-3.02 to -0.16] |
| Poland | 882.95 [801.85-926.12] | 219.16 [197.55-229.64] | 743.98 [678.39-810.87] | 126.09 [116.17-137.18] | -2.38 [-3.63 to -1.1] |
| Tonga | 0.98 [0.69-1.28] | 110.77 [78.63-145.14] | 1.08 [0.74-1.5] | 108.13 [74.47-147.47] | -1.95 [-4.62 to 0.79] |
| Cyprus | 14.7 [12.21-19.99] | 193.87 [160.27-263.39] | 22.18 [15.42-27.74] | 122.67 [85.22-152.83] | -1.43 [-3.4 to 0.58] |
| Serbia | 179.78 [145.19-213.62] | 181.79 [149.37-220.57] | 159.59 [118.78-196.77] | 116.7 [87.37-142.8] | -1.84 [-3.28 to -0.37] |
| Kuwait | 22.79 [20.91-24.91] | 172.96 [159.63-188.58] | 32.48 [26.59-39.22] | 83.85 [69.26-101.13] | -2.56 [-4.12 to -0.96] |
| Austria | 165.44 [158.16-173.24] | 174.8 [167.16-183.12] | 162.95 [148.02-176.17] | 110.59 [101.87-119.04] | -1.75 [-3.2 to -0.28] |
| Luxembourg | 10.99 [10.47-11.54] | 248.42 [236.18-260.96] | 11.91 [10.7-13.19] | 131.5 [119.18-146.54] | -2.89 [-4.44 to -1.32] |
| Afghanistan | 407.58 [189.52-650.88] | 421.02 [208.94-661.53] | 956.29 [558.4-1384.78] | 373.66 [215.7-553.13] | -0.42 [-1.97 to 1.15] |
| Peru | 529.85 [430.54-712.79] | 235.03 [193.28-307.49] | 653.35 [410.78-864.7] | 184.76 [116.19-244.32] | -0.59 [-2.51 to 1.36] |
| Brazil | 2605.29 [2477.96-2741.35] | 185.96 [178.64-194.38] | 3105.6 [2972.69-3227.73] | 136.6 [129.79-142.97] | -1.15 [-2.74 to 0.47] |
| Dominican Republic | 138.07 [109.95-171.3] | 179.17 [146.97-218.99] | 142.91 [107.15-185.14] | 133.35 [99.41-172.73] | -0.4 [-2.08 to 1.32] |
| Libya | 89.84 [70.93-114.47] | 243.41 [187.92-310.19] | 143.79 [103.48-198.68] | 229.86 [168.23-309.04] | -0.06 [-1.68 to 1.59] |
| Uruguay | 75.19 [71.67-79.18] | 224.63 [213.74-237.14] | 75.24 [69.62-81.4] | 180.01 [166.16-195.14] | -1.12 [-2.41 to 0.19] |
| Sudan | 633.31 [330.27-1021.19] | 298.06 [177.61-444.85] | 897.4 [542.56-1282.69] | 226.2 [136.95-323.49] | -0.88 [-2.49 to 0.75] |
| Mauritius | 16.12 [15.43-16.77] | 160.44 [153.65-166.8] | 16.98 [15.76-17.86] | 114.75 [107.23-120.33] | -0.06 [-2.2 to 2.14] |
| Tokelau | 0.03 [0.02-0.03] | 160.19 [105.94-216.56] | 0.04 [0.03-0.06] | 323.48 [190.23-494.19] | -1.86 [-4.85 to 1.21] |
| Bangladesh | 2184.2 [1253.98-3255.34] | 184.47 [123.32-254.67] | 1744.38 [1230.58-2348.51] | 109.76 [77.48-147.62] | -1.63 [-3.78 to 0.56] |
| South Africa | 379.51 [304.68-450.75] | 115.84 [89.95-136.21] | 580.63 [434.12-663.83] | 110.18 [82.09-126.48] | -0.51 [-2.96 to 2.01] |
| China | 39244.66 [29699.34-47269.63] | 343.57 [260.79-414.78] | 22052.21 [16128.39-27366.25] | 151.54 [108.71-185.06] | -2.84 [-4.6 to -1.06] |
| Malta | 6.01 [5.56-6.49] | 152.87 [140.51-164.88] | 7.71 [6.83-8.68] | 117.44 [103.09-132.15] | -1.71 [-3.36 to -0.04] |
| Guinea | 21.18 [14.04-29.18] | 25.48 [17.55-33.65] | 30.02 [14.79-55.06] | 19.52 [10.53-32.68] | -0.42 [-2.34 to 1.55] |
| Palestine | 50.72 [38.05-68.23] | 286.74 [216.94-377.86] | 72.96 [58.29-96.66] | 178.52 [145.01-229.9] | -1.53 [-2.97 to -0.07] |
| Netherlands | 285.7 [274.31-297.34] | 167.91 [161.5-174.55] | 286.82 [263.89-308.81] | 101.44 [94.56-109.25] | -2.08 [-3.62 to -0.52] |
| Democratic People's Republic of Korea | 500.12 [347.85-688.58] | 239.05 [166.41-328.45] | 505.36 [360.98-722.36] | 187.57 [134.5-265.5] | -0.69 [-2.4 to 1.05] |
| Morocco | 127.44 [92.91-171.48] | 51.82 [38.93-67.4] | 136.23 [94.79-183.53] | 37.97 [26.26-51.11] | -0.91 [-2.45 to 0.65] |
| Finland | 88.15 [84.47-91.82] | 146.99 [141.06-153.14] | 87.92 [80.38-94.99] | 95.77 [88.57-103.04] | -1.85 [-3.28 to -0.4] |
| United States of America | 5998.35 [5807.51-6131.69] | 210.71 [205.12-214.98] | 6463.96 [6033.16-6708.55] | 132.89 [125.97-137.57] | -1.85 [-3.43 to -0.24] |
| Rwanda | 109.77 [78.06-151.83] | 139.23 [102.65-185.51] | 120.14 [70.05-188.77] | 98.56 [60.31-151.34] | -1.63 [-3.05 to -0.19] |
| Bhutan | 9.24 [5.38-13.36] | 148.6 [93.73-207.54] | 7.66 [4.88-11.84] | 109.68 [71.25-166.91] | -1.14 [-3.42 to 1.19] |
| Costa Rica | 58.42 [55.51-61.33] | 208.18 [197.69-218.5] | 105.82 [95.16-115.69] | 215.21 [194.03-235.19] | -0.36 [-2.02 to 1.33] |
| Romania | 436.85 [414.74-457.75] | 186.62 [176.8-195.65] | 347.56 [299.25-392.78] | 128.38 [111.25-144.82] | -1.41 [-2.84 to 0.04] |
| Qatar | 6.16 [4.1-7.85] | 226.93 [157.98-287.29] | 20.45 [13.74-28.28] | 110.56 [76.68-149.68] | -2.63 [-3.86 to -1.38] |
| Burundi | 75.32 [54.5-103.78] | 120.41 [88.86-162.74] | 118.9 [61.38-190.78] | 96.33 [54.55-148.7] | -0.59 [-2.01 to 0.86] |
| Slovenia | 37.77 [35.2-40.4] | 172.18 [160.89-183.49] | 40.33 [34.02-46.86] | 110.2 [94.21-127.79] | -1.7 [-2.97 to -0.42] |
| Zimbabwe | 82.81 [55.43-101.98] | 101.04 [74.32-125.99] | 203.56 [139.86-271.6] | 153.41 [108.83-202.41] | 2.21 [-0.49 to 4.98] |
| Viet Nam | 802.97 [624.77-1050.92] | 126.57 [98.93-163.81] | 969.96 [718.03-1307.3] | 96.91 [72.06-129.69] | -0.94 [-3.7 to 1.9] |
| Bosnia and Herzegovina | 55.61 [45.99-66.59] | 127.68 [106.5-153.97] | 50.81 [37.4-64.28] | 103.69 [76.82-130.05] | -0.63 [-2.2 to 0.96] |
| Albania | 49.25 [40.03-61.47] | 157.32 [128.87-200.85] | 30.75 [21.38-44.74] | 97.61 [67.66-137.85] | -1.29 [-3.05 to 0.5] |
| Seychelles | 1.63 [1.38-2] | 241.44 [205.12-299.82] | 1.76 [1.42-2.12] | 154.47 [124.91-185.95] | -0.93 [-2.1 to 0.26] |
| Mali | 58.03 [37.22-80.43] | 50.66 [35.16-65.62] | 96.24 [53.94-166.85] | 35.37 [21.68-53.52] | -0.86 [-2.72 to 1.04] |
| Norway | 78.74 [75.95-81.14] | 149.71 [145.38-154.08] | 83.64 [77.21-88.58] | 101.59 [95.7-106.82] | -1.46 [-3.1 to 0.21] |
| Brunei Darussalam | 5.25 [4.23-6.83] | 234.05 [190.88-296.09] | 6.48 [4.95-7.75] | 148.58 [112.66-177.34] | -1.21 [-2.87 to 0.49] |
| Timor-Leste | 19.37 [9.98-29.06] | 235.84 [144.06-333.19] | 24.41 [18.06-31.43] | 179.2 [133.61-234.58] | -0.85 [-2.8 to 1.14] |
| Nepal | 317.78 [189.49-460.38] | 154.57 [104.34-207.17] | 315.18 [229.69-438.51] | 107.65 [79.16-149.83] | -0.84 [-3.04 to 1.41] |
| Tuvalu | 0.17 [0.12-0.25] | 188.55 [134.09-262.73] | 0.17 [0.12-0.22] | 142.63 [96.75-184] | -2.34 [-5.26 to 0.68] |
| Japan | 2247.26 [2203.03-2282.82] | 164.86 [161.99-167.32] | 2064.97 [1882.24-2178.96] | 91.59 [87.07-94.71] | -2.28 [-4.28 to -0.25] |
| Liberia | 17.69 [10.55-27.25] | 55.68 [39.1-79.85] | 30.82 [14.17-46.66] | 57.15 [26.34-84.59] | 0.63 [-1.21 to 2.5] |
| Guinea-Bissau | 8.03 [5.25-11.93] | 67.27 [46.04-96.84] | 13.32 [7.17-18.51] | 67.23 [38.16-90.71] | 0.56 [-1.2 to 2.35] |
| United States Virgin Islands | 1.65 [1.3-2.04] | 162.18 [127.41-199.03] | 1.04 [0.75-1.37] | 100.51 [72.53-135.53] | -1.18 [-2.82 to 0.49] |
| Eswatini | 7.37 [5.37-10.99] | 115.95 [87.4-164.14] | 12.94 [8.17-19.39] | 136.85 [89.36-204.53] | 0.75 [-1.83 to 3.4] |
| Spain | 864.78 [830.1-895.62] | 198.78 [191.1-206.12] | 780.3 [703.25-845.43] | 108.28 [99.82-116.3] | -2.57 [-3.94 to -1.17] |
| Niger | 67.97 [41.48-100] | 59.96 [41.65-82.1] | 151.29 [66.64-242.35] | 52.94 [24.21-84.51] | 0.05 [-1.79 to 1.93] |
| Slovakia | 108.83 [91.63-134.2] | 194.96 [164.08-239.97] | 100.58 [71.89-129.16] | 132.4 [95.17-170.41] | -1.67 [-3.28 to -0.03] |
| Saint Vincent and the Grenadines | 2.19 [1.99-2.4] | 208.73 [191.18-227.03] | 2.01 [1.75-2.32] | 168.7 [146.3-194.38] | -0.78 [-1.92 to 0.38] |
| Taiwan (Province of China) | 223.06 [213.69-232.74] | 113.87 [109.08-118.68] | 337.91 [309.8-364.2] | 114.16 [105.12-123.11] | 0.06 [-1.81 to 1.97] |
| Bahrain | 8.37 [6.36-10.08] | 247.61 [183.63-296.4] | 16.7 [11.99-22.6] | 137.5 [100.29-180.84] | -2.63 [-5.02 to -0.19] |
| Bulgaria | 170.5 [155.87-186.13] | 182.15 [165.74-198.55] | 149.08 [121.11-180.3] | 151.83 [123.46-183.06] | -0.75 [-2.14 to 0.67] |
| Guatemala | 175.37 [162.3-187.97] | 181.45 [171.2-190.46] | 291.6 [250.94-339.64] | 186.28 [160.05-217.3] | -0.62 [-2.59 to 1.39] |
| Denmark | 146.75 [140.78-152.9] | 226.49 [217.17-235.24] | 128.37 [117.38-138.94] | 130.57 [120.77-141.57] | -2.12 [-3.49 to -0.74] |
| Singapore | 48.83 [46.35-51.43] | 174.69 [165.83-184.23] | 55.07 [50.96-59.15] | 87.57 [80.79-95.13] | -2.5 [-4.67 to -0.28] |
| Burkina Faso | 64.7 [46.34-90.91] | 53.26 [39.72-68.23] | 158.45 [75.45-226.61] | 61.22 [29.43-85.05] | 0.9 [-0.88 to 2.71] |
| Haiti | 248.78 [109.28-413.06] | 351.92 [184.02-550.64] | 327.08 [182.8-513.2] | 260.85 [158.21-396.25] | -0.72 [-2.23 to 0.81] |
| Belarus | 264.32 [246.62-279.66] | 241.32 [224.1-256.23] | 182.16 [149.46-222.02] | 144.2 [119.18-175.05] | -2.14 [-3.38 to -0.89] |
| Comoros | 5.37 [3.52-7.47] | 106.97 [74.07-147.18] | 7.28 [4.7-10.64] | 104.04 [68.7-148.9] | -0.27 [-1.83 to 1.32] |
| France | 1372.89 [1308.94-1435.79] | 200.63 [191.87-209.14] | 1410.57 [1259.99-1549.85] | 125.11 [114.44-135.56] | -1.89 [-3.2 to -0.56] |
| United Republic of Tanzania | 347.17 [267.91-442.2] | 115.25 [92.37-143.33] | 604.79 [361.37-902.14] | 103.24 [64.81-151.88] | -0.14 [-1.67 to 1.41] |
| Nauru | 0.2 [0.14-0.26] | 216.22 [156.91-289.32] | 0.21 [0.13-0.31] | 203.41 [132.56-297.91] | -2.01 [-4.93 to 1.01] |
| Democratic Republic of the Congo | 310.56 [211.23-459.12] | 80.04 [58.48-106.34] | 546.78 [351.26-764.11] | 71.26 [44.49-104.27] | -0.07 [-2.13 to 2.02] |
| South Sudan | 68.53 [44.28-107.73] | 105.5 [73.71-154] | 122.5 [80.49-178.37] | 124.51 [82.26-172.74] | 0.74 [-0.8 to 2.3] |
| Somalia | 87.73 [52.68-134.31] | 109.45 [72.93-157.98] | 204.89 [128.84-299.24] | 109.61 [70.87-154.64] | 0.3 [-1.07 to 1.68] |
| Saint Lucia | 2.31 [2.14-2.52] | 185.84 [174.29-200.1] | 2.49 [2.05-3] | 131.75 [108.56-159.21] | -1.42 [-2.59 to -0.25] |
| Kiribati | 1.04 [0.67-1.34] | 139 [92.25-174.58] | 1.54 [0.85-2.32] | 132.06 [75.7-190.85] | -1.73 [-4.79 to 1.42] |
| Georgia | 130.62 [121.53-140.77] | 237.85 [221.01-256.95] | 72.66 [65.13-80.72] | 165.23 [148.85-182.16] | -1.13 [-2.6 to 0.36] |
| Guam | 1.72 [1.39-2.1] | 141.24 [116.2-173.63] | 1.85 [1.42-2.23] | 108.4 [82.85-131.51] | -3.02 [-5.97 to 0.03] |
| Oman | 23.57 [16.68-31.92] | 152.28 [107.5-208.03] | 29.67 [22.24-38.67] | 89.31 [67.65-114.25] | -1.71 [-3.29 to -0.1] |
| Mexico | 2133.69 [2034.6-2246.53] | 235.39 [227.05-244.97] | 2555.66 [2328.45-2792.45] | 198.89 [180.95-217.37] | -0.98 [-2.99 to 1.08] |
| Antigua and Barbuda | 0.91 [0.84-0.99] | 156.6 [144.63-169.7] | 1.19 [1.12-1.26] | 127.75 [120.44-135.75] | -0.94 [-2.76 to 0.91] |
| Djibouti | 4 [2.59-5.97] | 94.82 [62.68-139.3] | 10.58 [5.82-18.04] | 96.13 [54.21-157.65] | 0.25 [-1.3 to 1.84] |
| Czechia | 264.96 [249.5-281.9] | 222.62 [209.57-236.57] | 236.52 [202.42-272.64] | 132.89 [113.47-153.08] | -1.97 [-3.25 to -0.67] |
| Egypt | 1047.52 [858.49-1543.09] | 196.86 [163.56-287.26] | 1947.95 [1302.21-2491.53] | 214.87 [148.57-270.79] | 1.1 [-0.44 to 2.66] |
| Uganda | 152.48 [104.03-205.69] | 75.07 [53.09-96.79] | 332.68 [177-525.81] | 76.19 [45.43-111.06] | -0.15 [-1.59 to 1.31] |
| Sweden | 175.38 [166.55-184.02] | 157.94 [150.63-165.53] | 162.4 [143.83-181.19] | 94.73 [85.44-104.28] | -1.51 [-3.19 to 0.19] |
| Germany | 1861.54 [1784.93-1931.26] | 188.29 [181.46-194.77] | 1841.38 [1680.68-1977.9] | 121.05 [112.99-128.97] | -1.71 [-3.23 to -0.18] |
| Pakistan | 1822.49 [1214.15-2434.44] | 158.06 [112.51-200.05] | 3399.26 [2519.32-4670.99] | 149.82 [112.87-203.55] | -0.41 [-2.61 to 1.85] |
| Honduras | 141.98 [116.3-177.73] | 272.23 [222.93-331.25] | 166.21 [112.28-249.78] | 182.25 [126.14-258.82] | -0.88 [-2.62 to 0.88] |
| Armenia | 85.02 [80.31-89.9] | 251.55 [238.5-264.85] | 45.47 [40.34-51.37] | 132.63 [118.3-148.51] | -1.77 [-3.12 to -0.4] |
| Ethiopia | 2087.44 [901.38-3411.09] | 421.97 [237.4-614.5] | 2453.73 [1712.45-3503.77] | 252.01 [178.46-367.3] | -2.09 [-3.65 to -0.51] |
| Iraq | 471.67 [351.91-668.03] | 273.41 [211.14-383.45] | 700.46 [524.11-912.96] | 198.36 [150.4-255.89] | -0.97 [-2.58 to 0.67] |
| Syrian Arab Republic | 386.38 [291.28-495.14] | 329.82 [246.67-407.74] | 287.76 [207.91-395.52] | 204.7 [147.58-279.86] | -1.77 [-3.26 to -0.26] |
| Turkey | 2011.33 [1439.48-2616.82] | 367.06 [269.48-461.34] | 1550.51 [1143.43-1913.87] | 178.55 [131.27-219.53] | -2.37 [-4.05 to -0.66] |
| Montenegro | 9.53 [7.63-11.54] | 154.03 [123.54-185.97] | 9.56 [7.66-12.11] | 116.84 [92.81-145.18] | -0.91 [-2.49 to 0.69] |
| Cameroon | 66.89 [48.36-90.84] | 54.46 [41.24-70.73] | 197.19 [91.97-285.13] | 60.78 [29.2-88.01] | 0.77 [-1.09 to 2.66] |
| Maldives | 4.58 [2.32-7.29] | 207.13 [120.35-307.4] | 3.98 [2.83-5.38] | 89.57 [64.96-118.43] | -3.36 [-5.34 to -1.33] |
| Senegal | 52.95 [39.25-69.3] | 54.89 [42.12-69.48] | 86.23 [40.33-140.61] | 54.82 [26.82-84.89] | 0.59 [-1.35 to 2.56] |
| Chad | 35.12 [23.83-49.95] | 45.12 [33.61-60.39] | 131.8 [69.65-201.69] | 63.16 [32.59-91.69] | 1.53 [-0.31 to 3.4] |
| Palau | 0.15 [0.11-0.2] | 104.68 [74.37-137.95] | 0.16 [0.12-0.2] | 90.16 [69.27-111.38] | -0.32 [-1.5 to 0.87] |
| Mongolia | 36.77 [28.27-48.92] | 160.47 [125.55-205.69] | 35.12 [25.9-44.96] | 108.12 [80.38-137.35] | -1.41 [-3.31 to 0.54] |
| Papua New Guinea | 60.78 [31.63-87.32] | 151.63 [76.66-214.16] | 139.64 [82.48-195.82] | 136.43 [78.21-196.38] | -2.14 [-5.26 to 1.08] |
| India | 11312.88 [8624.08-14427.99] | 135.48 [107.52-166.12] | 12726.23 [10182.72-15366.78] | 94.82 [75.53-114.8] | -1.08 [-3.01 to 0.89] |
| Monaco | 1.27 [0.97-1.71] | 293.94 [228.58-383.03] | 1.85 [1.38-2.28] | 302.64 [223.33-373.64] | -0.18 [-1.8 to 1.47] |
| Portugal | 227.12 [217.19-236.13] | 208.07 [198.45-216.75] | 200.36 [179.66-216.71] | 113.78 [104.86-122.89] | -2.39 [-3.97 to -0.79] |
| Algeria | 330.43 [245.3-410.62] | 130.63 [97.98-158.6] | 338.85 [248.98-436.09] | 83.21 [61.37-107.07] | -1.27 [-3.08 to 0.57] |
| Switzerland | 154.92 [147.82-162.12] | 190.16 [182.02-199.22] | 142.6 [127.44-157.37] | 100.66 [91.02-110.76] | -2.28 [-3.72 to -0.81] |
| Mauritania | 10.61 [7.79-14.24] | 46.16 [35.79-58.38] | 20.29 [10.16-31.62] | 46.94 [24.14-70.21] | 0.15 [-1.83 to 2.16] |
| Greece | 262.26 [251.44-271.58] | 207.68 [200.56-214.81] | 299.17 [277.53-318.85] | 166.28 [156.39-175.86] | -1.55 [-2.79 to -0.3] |
| Gabon | 7.35 [5.56-9.39] | 80.67 [60.86-101.44] | 12.06 [6.99-18.02] | 75.79 [45.3-113.38] | -0.18 [-2.29 to 1.98] |
| Tunisia | 110.2 [83.83-137.23] | 137.13 [104.73-170.3] | 109.93 [76.83-150.14] | 89.6 [62.89-121.73] | -1.45 [-3.01 to 0.13] |
| North Macedonia | 34.44 [28.78-41.97] | 180.31 [151.36-220.29] | 34.06 [24.07-43.18] | 123.32 [86.35-157.34] | -1.53 [-9.08 to 6.64] |
| Barbados | 5.18 [4.86-5.48] | 199.03 [185.9-211.23] | 6.01 [4.67-7.46] | 163.64 [127.3-205.71] | -0.53 [-2.01 to 0.98] |
| Indonesia | 3937.05 [2874.69-5262.8] | 218.78 [162.25-286.35] | 4895.01 [3947.07-6231.93] | 182.22 [146.79-232.04] | -0.61 [-2.54 to 1.35] |
| Nicaragua | 100.75 [83.23-129.85] | 215.58 [181.15-269.19] | 88.92 [61.74-109.39] | 137.89 [96.32-168.47] | -0.84 [-2.63 to 0.98] |
| Saudi Arabia | 172.99 [122.15-264.61] | 120.56 [82.9-196.92] | 387.77 [273-601.09] | 117.11 [86.82-171.03] | 0.36 [-0.84 to 1.58] |
| Iceland | 3.97 [3.67-4.25] | 148.36 [137.49-158.88] | 5.11 [4.58-5.65] | 107.89 [97.73-118.75] | -1.47 [-3 to 0.09] |
| Latvia | 75.42 [70.55-79.79] | 260.21 [243.85-275.55] | 40.97 [35.12-47.34] | 140.71 [121.07-162.2] | -2.18 [-3.47 to -0.88] |
| Cuba | 204.35 [195.4-213.24] | 192.13 [183.72-200.14] | 181.02 [157.08-205.76] | 129.84 [113.33-147.66] | -1.35 [-2.91 to 0.23] |
| Venezuela (Bolivarian Republic of) | 401.09 [384.61-417.6] | 209.21 [201.44-217] | 483.25 [357.44-636.74] | 182.19 [134.31-240.05] | -0.9 [-2.65 to 0.88] |
| Australia | 334.79 [322.71-346.03] | 185.83 [179.31-192.08] | 465.51 [427.16-501.46] | 121.72 [113.34-130.54] | -2.13 [-3.43 to -0.81] |
| Suriname | 6.05 [4.24-7.09] | 160.87 [117.59-188.61] | 8.01 [5.99-10.17] | 137.56 [102.71-174.21] | -0.53 [-1.88 to 0.84] |
| United Kingdom | 1130.12 [1098.38-1150.98] | 160.31 [156.9-162.96] | 1170.96 [1100.76-1216.85] | 112.34 [107.58-116] | -1.46 [-3.19 to 0.31] |
| Zambia | 109.37 [79-152.16] | 118.55 [91.95-156.14] | 186.58 [110.08-274.9] | 103.8 [65.17-147.47] | -0.38 [-1.88 to 1.15] |
| Bahamas | 3.52 [3.23-3.8] | 149.6 [138-161.36] | 5.16 [4.11-6.44] | 128.38 [102.63-160.47] | -0.7 [-1.95 to 0.56] |
| Kyrgyzstan | 87.82 [78.73-96.65] | 191.6 [172.47-209.84] | 70.82 [58.38-84.11] | 105.92 [87.34-125.65] | -2.33 [-3.77 to -0.87] |
| Iran (Islamic Republic of) | 1767.17 [1101.6-2279.01] | 311.61 [193.52-390.17] | 1444.89 [958.94-1689.54] | 176.34 [116.43-205.76] | -1.41 [-2.88 to 0.08] |
| Israel | 108.96 [103.65-114.43] | 221.04 [210.16-232.2] | 150.4 [138.22-161.96] | 132.65 [123.13-142.35] | -1.96 [-3.67 to -0.22] |
| Trinidad and Tobago | 22.12 [20.82-23.57] | 192.07 [182.35-202.81] | 22.85 [17.58-29.04] | 150.95 [118.94-191.98] | -1.15 [-2.66 to 0.39] |
| Cabo Verde | 4.32 [3.21-5.98] | 108.57 [84.43-148.59] | 5.82 [4.28-7.48] | 110.41 [81.84-141.49] | -0.17 [-2.02 to 1.72] |
| Argentina | 752.59 [725.48-778.64] | 226.97 [218.77-234.65] | 740.7 [695.01-798.17] | 153.09 [143.18-165.64] | -1.51 [-2.97 to -0.03] |
| Bolivia (Plurinational State of) | 251.93 [168.87-351.85] | 365.79 [253.23-503.71] | 294.71 [205.1-396.16] | 260.74 [181.12-349.96] | -1.07 [-2.9 to 0.79] |
| Sao Tome and Principe | 0.62 [0.42-0.84] | 40.37 [28.29-52.29] | 0.55 [0.31-0.94] | 26.94 [16.83-42.04] | -0.94 [-2.83 to 0.98] |
| Malaysia | 376.86 [276.67-459.58] | 227.18 [176.45-268.32] | 535.29 [438.08-642.32] | 170.54 [139.92-204.89] | -0.92 [-2.89 to 1.08] |
| Sierra Leone | 29.22 [19.06-43.97] | 53.38 [39.2-74.75] | 55.41 [26.26-81.61] | 58.49 [28.03-84.42] | 0.87 [-0.99 to 2.77] |
| Northern Mariana Islands | 0.6 [0.39-0.81] | 159.78 [106.55-211.64] | 0.59 [0.44-0.7] | 116.46 [87.83-139.17] | -0.78 [-5.17 to 3.83] |
| Micronesia (Federated States of) | 1.81 [1.28-2.49] | 195.87 [140.03-270.54] | 1.59 [1.05-2.19] | 164.12 [108.17-226.41] | -2.14 [-5.01 to 0.82] |
| Eritrea | 37.99 [25.76-56.26] | 107.56 [78.6-152.12] | 67.63 [39.77-102.87] | 111.52 [68.45-159.26] | 0.21 [-1.22 to 1.66] |
| Republic of Moldova | 100.19 [94.59-106.26] | 226.47 [213.57-240.57] | 44.09 [39.51-49.71] | 99.7 [87.87-114.32] | -2.12 [-3.84 to -0.36] |

| **Country** | **1990** | | **2021** | | **1990-2021** |
| --- | --- | --- | --- | --- | --- |
|  | **Deaths cases** | **ASMR per 100,000** | **Deaths cases** | **ASMR per 100,000** | **EAPC** |
|  | **No. *10^2^ (95% UI)** | **No. (95% UI)** | **No. *10^2^ (95% UI)** | **No.(95% UI)** | **No. (95% CI)** |
| Mexico | 34.88 [33.75-36.16] | 4.89 [4.77-5.02] | 57.65 [51.96-63.33] | 4.54 [4.1-4.98] | -0.74 [-1.74 to 0.27] |
| Chile | 5.27 [5.05-5.49] | 4.71 [4.49-4.92] | 8.19 [7.53-8.8] | 3.45 [3.17-3.69] | -0.97 [-1.77 to -0.16] |
| Nicaragua | 1.46 [1.23-1.84] | 4.13 [3.56-5.15] | 1.88 [1.34-2.31] | 3.26 [2.38-3.98] | -0.51 [-1.42 to 0.41] |
| Poland | 28.08 [26.16-30.12] | 6.7 [6.23-7.18] | 33.1 [29.98-36.03] | 4.72 [4.3-5.13] | -1.47 [-2.25 to -0.68] |
| Palestine | 1.02 [0.78-1.34] | 8.49 [6.46-11.16] | 1.67 [1.35-2.14] | 5.66 [4.58-7.22] | -1.07 [-1.97 to -0.16] |
| Saint Lucia | 0.05 [0.05-0.06] | 5.07 [4.81-5.4] | 0.07 [0.06-0.09] | 3.49 [2.88-4.19] | -1.31 [-1.97 to -0.64] |
| Ireland | 2.52 [2.38-2.65] | 6.23 [5.87-6.53] | 2.7 [2.39-2.98] | 3.46 [3.09-3.79] | -1.84 [-2.76 to -0.91] |
| Thailand | 24.58 [18.49-29.07] | 5.83 [4.24-6.94] | 45.22 [25.98-60.3] | 4.93 [2.9-6.45] | -1.27 [-2.24 to -0.29] |
| Panama | 0.84 [0.78-0.9] | 4.09 [3.85-4.35] | 1.92 [1.57-2.28] | 4.4 [3.6-5.23] | 0.16 [-0.75 to 1.08] |
| Singapore | 1.13 [1.07-1.19] | 4.55 [4.26-4.8] | 1.88 [1.72-2.02] | 2.52 [2.32-2.71] | -1.69 [-3.06 to -0.29] |
| Saint Vincent and the Grenadines | 0.05 [0.04-0.05] | 5.3 [4.9-5.69] | 0.06 [0.05-0.06] | 4.36 [3.83-4.95] | -0.33 [-1.02 to 0.37] |
| Slovenia | 1.26 [1.17-1.36] | 5.36 [5.01-5.75] | 1.93 [1.63-2.23] | 4.27 [3.59-4.93] | -0.76 [-1.61 to 0.1] |
| Malta | 0.21 [0.2-0.23] | 5.22 [4.8-5.63] | 0.35 [0.3-0.4] | 3.86 [3.4-4.34] | -1.8 [-2.73 to -0.86] |
| Kyrgyzstan | 1.54 [1.38-1.69] | 3.83 [3.47-4.2] | 1.43 [1.19-1.7] | 2.4 [1.98-2.84] | -1.76 [-2.57 to -0.95] |
| United States of America | 218.59 [205.43-225.59] | 6.97 [6.59-7.18] | 297.86 [267.04-313.37] | 5.17 [4.69-5.41] | -1.22 [-2.22 to -0.22] |
| Kiribati | 0.02 [0.01-0.02] | 3.16 [2.09-3.95] | 0.03 [0.02-0.04] | 3.15 [1.86-4.24] | -0.97 [-2.67 to 0.75] |
| Brazil | 51.61 [49.95-53.39] | 4.56 [4.38-4.71] | 91.71 [86.2-95.6] | 3.87 [3.63-4.04] | -0.67 [-1.5 to 0.17] |
| Andorra | 0.04 [0.03-0.06] | 7.63 [5.21-11.33] | 0.07 [0.04-0.09] | 4.5 [2.95-6.2] | -1.6 [-2.56 to -0.62] |
| Lithuania | 2.8 [2.64-2.95] | 6.64 [6.27-7] | 2.7 [2.34-3.03] | 5.09 [4.42-5.71] | -0.88 [-1.68 to -0.07] |
| Bolivia (Plurinational State of) | 4.16 [2.88-5.72] | 7.98 [5.62-10.63] | 6.47 [4.46-8.69] | 6.46 [4.46-8.62] | -0.64 [-1.6 to 0.33] |
| Tunisia | 2.2 [1.69-2.73] | 3.57 [2.71-4.47] | 3.28 [2.3-4.53] | 2.64 [1.86-3.61] | -1.26 [-2.13 to -0.39] |
| Samoa | 0.06 [0.05-0.08] | 5.7 [4.41-7.37] | 0.08 [0.06-0.11] | 5.11 [3.82-6.86] | -1.41 [-3 to 0.21] |
| Taiwan (Province of China) | 4.9 [4.68-5.11] | 2.82 [2.68-2.94] | 11.97 [10.91-12.91] | 3.28 [3.01-3.53] | 0.78 [-0.36 to 1.93] |
| Sweden | 7.14 [6.7-7.53] | 5.04 [4.76-5.31] | 8.45 [7.31-9.47] | 3.7 [3.26-4.13] | -1 [-2.09 to 0.09] |
| Uzbekistan | 7.87 [7.13-8.66] | 4.41 [4-4.87] | 8.98 [7.41-10.93] | 2.83 [2.34-3.44] | -1.67 [-2.61 to -0.72] |
| Barbados | 0.15 [0.14-0.16] | 5.21 [4.92-5.5] | 0.22 [0.17-0.26] | 4.84 [3.8-5.93] | -0.1 [-1 to 0.82] |
| Finland | 3.26 [3.07-3.42] | 4.81 [4.55-5.03] | 4.29 [3.75-4.7] | 3.41 [3.08-3.68] | -1.41 [-2.3 to -0.52] |
| Bahrain | 0.18 [0.13-0.21] | 8.38 [6.09-10.17] | 0.43 [0.3-0.57] | 5.02 [3.67-6.54] | -1.68 [-3.14 to -0.2] |
| Croatia | 3.24 [2.81-3.73] | 5.77 [4.98-6.66] | 4.16 [3.49-4.92] | 4.74 [3.96-5.63] | -1.15 [-1.98 to -0.32] |
| Costa Rica | 1.23 [1.17-1.3] | 5.5 [5.14-5.8] | 2.91 [2.59-3.21] | 5.59 [4.98-6.17] | -0.3 [-1.17 to 0.58] |
| Malaysia | 7.21 [5.63-8.38] | 5.46 [4.5-6.56] | 13.34 [11.07-17.49] | 4.57 [3.82-6.14] | -0.52 [-1.65 to 0.63] |
| Kuwait | 0.44 [0.41-0.48] | 5 [4.56-5.45] | 0.83 [0.69-0.98] | 2.64 [2.16-3.11] | -1.98 [-2.88 to -1.06] |
| Grenada | 0.04 [0.03-0.04] | 4.78 [4.34-5.26] | 0.05 [0.04-0.05] | 4.49 [3.94-5.02] | -0.27 [-0.95 to 0.42] |
| Romania | 10.54 [10.06-11.03] | 4.22 [4.03-4.41] | 13.71 [11.94-15.41] | 4.05 [3.52-4.56] | -0.41 [-1.23 to 0.41] |
| Paraguay | 1.34 [1.07-1.61] | 4.27 [3.4-5.21] | 2.84 [2.09-3.61] | 4.64 [3.43-5.94] | 0.5 [-0.32 to 1.32] |
| India | 205.56 [163.29-250.21] | 3.21 [2.56-3.78] | 321.45 [262.15-390.98] | 2.63 [2.14-3.21] | -0.5 [-1.58 to 0.6] |
| Eswatini | 0.14 [0.11-0.2] | 3.47 [2.67-4.69] | 0.28 [0.18-0.42] | 3.98 [2.69-5.86] | 0.74 [-0.91 to 2.41] |
| Bermuda | 0.04 [0.03-0.04] | 5.88 [5.45-6.33] | 0.04 [0.03-0.05] | 3.34 [2.81-4.02] | -2.11 [-3.07 to -1.13] |
| Malawi | 0.8 [0.53-1.04] | 0.89 [0.64-1.09] | 1.02 [0.55-1.68] | 0.78 [0.49-1.13] | -0.21 [-1.11 to 0.7] |
| American Samoa | 0.01 [0.01-0.01] | 3.72 [2.97-4.55] | 0.02 [0.01-0.02] | 3.63 [2.75-4.48] | 0.13 [-1.72 to 2.02] |
| Senegal | 0.77 [0.59-0.99] | 1.12 [0.85-1.38] | 1.54 [0.77-2.36] | 1.32 [0.69-1.92] | 0.75 [-0.4 to 1.91] |
| Sao Tome and Principe | 0.01 [0.01-0.01] | 0.8 [0.6-0.99] | 0.01 [0.01-0.02] | 0.68 [0.5-0.9] | -0.05 [-1.21 to 1.12] |
| Belarus | 7.16 [6.71-7.57] | 6.02 [5.63-6.36] | 6.43 [5.29-7.81] | 4.41 [3.64-5.31] | -1.14 [-1.92 to -0.35] |
| Egypt | 18.84 [15.63-27.62] | 4.83 [3.85-7.68] | 45 [31.26-56.76] | 6.4 [4.68-8.13] | 1.07 [0.21 to 1.94] |
| Guam | 0.04 [0.03-0.04] | 3.87 [3.18-4.8] | 0.05 [0.04-0.06] | 2.65 [2.05-3.18] | -2.91 [-4.54 to -1.26] |
| Libya | 1.78 [1.37-2.24] | 6.74 [4.85-8.71] | 3.6 [2.64-4.84] | 6.57 [4.8-8.62] | -0.21 [-1.14 to 0.73] |
| Cameroon | 1.02 [0.76-1.35] | 1.2 [0.93-1.51] | 3.21 [1.54-4.64] | 1.41 [0.72-2.06] | 0.68 [-0.42 to 1.8] |
| Angola | 1.63 [1.07-2.35] | 2.25 [1.62-2.97] | 3.97 [2.49-5.76] | 2.06 [1.21-3.18] | -0.17 [-1.43 to 1.12] |
| Somalia | 1.36 [0.86-1.98] | 2.71 [1.89-3.73] | 3.43 [2.21-4.92] | 2.94 [1.99-4.11] | 0.33 [-0.49 to 1.15] |
| South Sudan | 1.11 [0.77-1.63] | 2.48 [1.81-3.46] | 2.01 [1.33-2.8] | 3.04 [2-4.16] | 0.43 [-0.47 to 1.34] |
| Gambia | 0.05 [0.03-0.06] | 0.58 [0.41-0.73] | 0.1 [0.06-0.13] | 0.58 [0.4-0.75] | 0.18 [-0.91 to 1.29] |
| Ghana | 2.93 [1.52-4.02] | 2.35 [1.31-3.13] | 3.49 [2.38-5.09] | 1.34 [0.96-1.93] | -1.84 [-2.98 to -0.7] |
| Gabon | 0.15 [0.12-0.19] | 2.18 [1.58-2.87] | 0.27 [0.16-0.39] | 2.14 [1.32-3.09] | 0.12 [-1.19 to 1.45] |
| Northern Mariana Islands | 0.01 [0.01-0.02] | 4.81 [3.24-6.25] | 0.02 [0.01-0.02] | 3.56 [2.72-4.21] | -0.97 [-3.61 to 1.73] |
| Botswana | 0.23 [0.16-0.31] | 3.14 [2.18-4.29] | 0.46 [0.31-0.67] | 2.88 [1.99-4.09] | -0.29 [-1.96 to 1.41] |
| Palau | 0 [0-0] | 2.73 [1.93-3.61] | 0 [0-0.01] | 2.32 [1.78-2.87] | -0.42 [-1.13 to 0.3] |
| Mali | 0.83 [0.57-1.1] | 1.01 [0.74-1.24] | 1.47 [0.88-2.34] | 0.8 [0.53-1.11] | -0.7 [-1.81 to 0.41] |
| Djibouti | 0.06 [0.04-0.09] | 2.23 [1.49-3.26] | 0.21 [0.12-0.34] | 2.6 [1.58-4] | 0.76 [-0.17 to 1.69] |
| Tokelau | 0 [0-0] | 4.21 [2.78-5.6] | 0 [0-0] | 5.73 [3.62-8.09] | -1.64 [-3.31 to 0.05] |
| Eritrea | 0.58 [0.41-0.84] | 2.52 [1.9-3.42] | 1.2 [0.74-1.71] | 2.83 [1.83-3.81] | 0.36 [-0.48 to 1.21] |
| Republic of Moldova | 2.22 [2.13-2.33] | 5.06 [4.84-5.3] | 1.46 [1.33-1.63] | 2.77 [2.49-3.12] | -1.84 [-2.8 to -0.87] |
| Tuvalu | 0 [0-0] | 4.75 [3.35-6.31] | 0 [0-0.01] | 3.81 [2.55-4.79] | -1.66 [-3.32 to 0.02] |
| Timor-Leste | 0.3 [0.17-0.43] | 5.31 [3.62-7.09] | 0.48 [0.36-0.64] | 4.43 [3.29-5.84] | -0.79 [-1.85 to 0.28] |
| Niger | 0.91 [0.6-1.28] | 1.15 [0.88-1.48] | 2.27 [1.02-3.59] | 1.23 [0.59-1.95] | 0.16 [-0.93 to 1.25] |
| Nepal | 5.3 [3.55-7.13] | 3.55 [2.61-4.72] | 7.3 [5.35-9.98] | 2.91 [2.16-3.97] | -0.67 [-1.91 to 0.59] |
| Brunei Darussalam | 0.1 [0.08-0.13] | 6.03 [4.92-7.45] | 0.16 [0.12-0.19] | 4.23 [3.11-5.12] | -1.14 [-2.13 to -0.15] |
| Cook Islands | 0 [0-0] | 1.78 [1.27-2.24] | 0 [0-0] | 1.15 [0.81-1.46] | -2.81 [-4.34 to -1.25] |
| Pakistan | 31.94 [22.77-40.59] | 3.77 [2.83-4.72] | 62.01 [47.09-84.1] | 3.61 [2.78-4.83] | 0.12 [-1.1 to 1.35] |
| Sierra Leone | 0.43 [0.31-0.6] | 1.04 [0.77-1.35] | 0.88 [0.42-1.27] | 1.28 [0.63-1.83] | 0.65 [-0.43 to 1.75] |
| North Macedonia | 0.91 [0.76-1.14] | 4.99 [4.17-6.27] | 1.27 [0.93-1.6] | 4.33 [3.18-5.42] | -0.41 [-5.38 to 4.83] |
| Marshall Islands | 0.01 [0.01-0.01] | 4.2 [2.95-5.43] | 0.02 [0.01-0.02] | 4.19 [2.78-5.76] | -0.81 [-2.47 to 0.87] |
| Central African Republic | 0.47 [0.33-0.66] | 2.53 [1.87-3.22] | 0.83 [0.51-1.18] | 2.38 [1.5-3.25] | 0.01 [-1.19 to 1.22] |
| Australia | 11.67 [11.06-12.17] | 6.18 [5.84-6.44] | 22.62 [20.16-24.68] | 4.88 [4.41-5.3] | -1.4 [-2.21 to -0.58] |
| Monaco | 0.06 [0.04-0.08] | 8.87 [6.74-11.87] | 0.09 [0.07-0.11] | 9.78 [7.29-12.12] | -0.07 [-1.07 to 0.93] |
| Madagascar | 1.97 [1.54-2.57] | 2.13 [1.63-2.66] | 3.83 [2.52-5.26] | 2.05 [1.32-2.84] | -0.17 [-1.05 to 0.73] |
| Niue | 0 [0-0] | 3.86 [2.67-5] | 0 [0-0] | 5.21 [3.82-6.34] | -0.95 [-2.62 to 0.74] |
| South Africa | 8.18 [6.11-9.76] | 3.18 [2.23-4.01] | 15.76 [11.51-18.19] | 3.39 [2.42-3.92] | -0.22 [-1.81 to 1.39] |
| Montenegro | 0.26 [0.21-0.32] | 4.28 [3.46-5.2] | 0.36 [0.28-0.47] | 4.04 [3.14-5.18] | -0.52 [-1.45 to 0.42] |
| Solomon Islands | 0.09 [0.04-0.13] | 4.29 [2.17-6.28] | 0.2 [0.12-0.27] | 4.2 [2.64-5.71] | -1.27 [-2.91 to 0.4] |
| Tonga | 0.02 [0.01-0.03] | 3.11 [2.21-4.16] | 0.03 [0.02-0.03] | 2.98 [2.11-3.92] | -1.43 [-2.98 to 0.15] |
| Ethiopia | 35.36 [18.54-53.12] | 10.61 [7.1-14.45] | 45.45 [32.39-65.94] | 6.81 [4.8-10.5] | -1.6 [-2.58 to -0.62] |
| Austria | 6.4 [6.02-6.73] | 5.69 [5.38-5.97] | 8.24 [7.24-9] | 4.37 [3.93-4.75] | -1.3 [-2.21 to -0.37] |
| Lebanon | 1.48 [1.01-2.19] | 6.55 [4.55-9.49] | 3.09 [2.4-3.89] | 5.02 [3.88-6.29] | -0.87 [-1.72 to 0] |
| Puerto Rico | 1.99 [1.89-2.11] | 5.57 [5.28-5.9] | 2.65 [2.16-3.14] | 4.16 [3.42-4.87] | -1.12 [-2.01 to -0.23] |
| Mauritius | 0.36 [0.34-0.37] | 4.22 [4.04-4.4] | 0.53 [0.49-0.55] | 3.22 [2.99-3.38] | 1.6 [-0.29 to 3.52] |
| Oman | 0.46 [0.32-0.62] | 4.42 [3.06-6.01] | 0.7 [0.52-0.91] | 3.04 [2.3-3.86] | -1.21 [-2.29 to -0.12] |
| France | 53.48 [49.99-57.02] | 6.69 [6.3-7.08] | 75.14 [65.45-83.62] | 4.93 [4.4-5.44] | -1.46 [-2.28 to -0.62] |
| Qatar | 0.12 [0.08-0.16] | 7.83 [5.51-9.94] | 0.45 [0.3-0.63] | 4.06 [2.84-5.48] | -1.68 [-2.52 to -0.83] |
| Burkina Faso | 0.97 [0.72-1.26] | 1.14 [0.87-1.39] | 2.46 [1.19-3.44] | 1.38 [0.67-1.88] | 0.51 [-0.53 to 1.56] |
| Seychelles | 0.04 [0.03-0.05] | 6.55 [5.58-8.2] | 0.05 [0.04-0.06] | 4.71 [3.77-5.72] | -0.45 [-1.15 to 0.26] |
| Côte d'Ivoire | 0.78 [0.54-1.01] | 0.88 [0.6-1.09] | 1.62 [0.91-2.43] | 0.83 [0.48-1.17] | -0.02 [-1.1 to 1.07] |
| Zambia | 1.63 [1.24-2.2] | 2.68 [2.15-3.46] | 3.19 [1.98-4.6] | 2.64 [1.74-3.59] | 0.22 [-0.68 to 1.13] |
| Slovakia | 3.41 [2.89-4.21] | 5.87 [4.98-7.22] | 3.91 [2.77-5.01] | 4.4 [3.15-5.64] | -1.26 [-2.28 to -0.24] |
| San Marino | 0.03 [0.02-0.04] | 8.91 [7.25-11.86] | 0.03 [0.02-0.05] | 4.48 [2.94-6.28] | -1.52 [-2.35 to -0.68] |
| Liberia | 0.26 [0.18-0.38] | 1.12 [0.86-1.45] | 0.51 [0.23-0.75] | 1.33 [0.63-1.9] | 0.58 [-0.51 to 1.68] |
| Latvia | 2.24 [2.09-2.38] | 6.88 [6.45-7.3] | 1.66 [1.41-1.91] | 4.57 [3.92-5.25] | -1.47 [-2.29 to -0.64] |
| Israel | 3.52 [3.29-3.73] | 7.34 [6.81-7.77] | 6.66 [5.88-7.29] | 5.24 [4.68-5.71] | -1.21 [-2.31 to -0.1] |
| Suriname | 0.12 [0.09-0.14] | 3.78 [2.93-4.53] | 0.2 [0.15-0.26] | 3.31 [2.46-4.29] | -0.27 [-0.99 to 0.46] |
| Uruguay | 2.28 [2.16-2.42] | 6.26 [5.94-6.61] | 2.91 [2.63-3.15] | 5.57 [5.12-6.02] | -0.44 [-1.23 to 0.35] |
| Netherlands | 10.73 [10.03-11.28] | 5.57 [5.24-5.85] | 13.9 [12.43-15.09] | 3.96 [3.58-4.29] | -1.55 [-2.49 to -0.61] |
| United Republic of Tanzania | 5.37 [4.25-6.7] | 2.61 [2.06-3.33] | 10.34 [6.5-15.14] | 2.51 [1.64-3.57] | -0.12 [-1.01 to 0.77] |
| Norway | 3.13 [2.94-3.26] | 4.83 [4.59-5] | 4.13 [3.69-4.41] | 4.02 [3.65-4.27] | -1.13 [-2.17 to -0.09] |
| Turkey | 39.97 [29.96-49.01] | 9.15 [6.93-10.99] | 48.04 [36.24-59.51] | 5.45 [4.12-6.73] | -1.72 [-2.65 to -0.79] |
| Ecuador | 3.74 [3.54-3.96] | 4.81 [4.54-5.07] | 8.95 [7.33-10.91] | 5.27 [4.32-6.43] | 0.79 [-0.21 to 1.79] |
| Zimbabwe | 1.69 [1.23-2.1] | 3.11 [2.39-4.02] | 4.03 [2.86-5.32] | 4.22 [3.13-5.48] | 1.05 [-0.67 to 2.8] |
| Armenia | 1.71 [1.63-1.78] | 5.46 [5.23-5.69] | 1.39 [1.22-1.6] | 3.58 [3.16-4.06] | -1.11 [-1.88 to -0.33] |
| Lesotho | 0.26 [0.19-0.35] | 2.63 [1.91-3.51] | 0.54 [0.39-0.73] | 4.35 [3.19-5.82] | 1.75 [0.1 to 3.43] |
| Switzerland | 5.8 [5.45-6.11] | 5.88 [5.57-6.17] | 7.16 [6.2-8.08] | 3.8 [3.37-4.25] | -2.28 [-3.19 to -1.36] |
| Afghanistan | 8.6 [4.52-13.25] | 10.76 [5.98-16.08] | 17.18 [9.98-24.83] | 9.98 [5.97-14.58] | -0.4 [-1.25 to 0.45] |
| El Salvador | 2.35 [2.04-2.95] | 5.05 [4.4-6.37] | 3.14 [2.24-3.88] | 4.92 [3.51-6.08] | -0.25 [-1.3 to 0.81] |
| Belize | 0.05 [0.04-0.05] | 2.95 [2.78-3.15] | 0.1 [0.09-0.11] | 2.86 [2.53-3.19] | 0.34 [-0.42 to 1.1] |
| Cabo Verde | 0.08 [0.06-0.11] | 2.3 [1.8-3.26] | 0.14 [0.1-0.17] | 2.89 [2.19-3.68] | 0.57 [-0.55 to 1.7] |
| Mongolia | 0.59 [0.46-0.76] | 3.25 [2.54-4.08] | 0.71 [0.53-0.9] | 2.44 [1.83-3.07] | -0.85 [-1.93 to 0.24] |
| Guinea | 0.3 [0.21-0.4] | 0.47 [0.35-0.59] | 0.46 [0.24-0.78] | 0.41 [0.25-0.62] | -0.24 [-1.37 to 0.89] |
| Guyana | 0.2 [0.17-0.22] | 3.48 [3.11-3.86] | 0.23 [0.17-0.29] | 3.28 [2.52-4.13] | 0.48 [-0.37 to 1.35] |
| Albania | 1.01 [0.83-1.29] | 4.09 [3.36-5.32] | 1.15 [0.82-1.72] | 2.99 [2.12-4.33] | -0.94 [-1.87 to -0.01] |
| Sudan | 10.58 [6.28-15.78] | 6.9 [4.51-9.54] | 16.79 [10.2-23.96] | 5.78 [3.57-8.22] | -0.66 [-1.53 to 0.22] |
| Algeria | 5.78 [4.31-6.98] | 3.4 [2.52-4.12] | 8.73 [6.41-11.51] | 2.57 [1.85-3.35] | -1.31 [-2.34 to -0.27] |
| Republic of Korea | 17.54 [14.43-21.62] | 4.68 [4.02-6.01] | 19.5 [12.11-23.9] | 2.41 [1.53-2.94] | -2.69 [-3.83 to -1.55] |
| Argentina | 19.23 [18.49-19.93] | 5.97 [5.74-6.18] | 23.82 [21.98-25.67] | 4.46 [4.14-4.8] | -0.71 [-1.48 to 0.07] |
| Iraq | 8.56 [6.63-11.96] | 6.81 [5.41-9.54] | 16.03 [12.22-20.73] | 5.8 [4.41-7.51] | -0.42 [-1.37 to 0.55] |
| Canada | 20.11 [18.88-20.94] | 6.36 [5.97-6.61] | 32.2 [28.57-34.91] | 4.43 [4.01-4.78] | -1.44 [-2.38 to -0.49] |
| Jordan | 1.68 [1.35-2.09] | 7.97 [6.27-10.01] | 4.32 [3.12-5.69] | 5.24 [3.83-6.88] | -1.39 [-2.51 to -0.26] |
| Sri Lanka | 6.98 [5.72-8.45] | 5.24 [4.32-6.47] | 7.72 [5.09-10.68] | 3.11 [2.09-4.29] | -0.91 [-2.33 to 0.53] |
| Bahamas | 0.07 [0.07-0.08] | 3.74 [3.46-4.04] | 0.13 [0.11-0.16] | 3.4 [2.76-4.16] | -0.61 [-1.35 to 0.13] |
| Jamaica | 0.72 [0.67-0.78] | 3.43 [3.19-3.69] | 1.1 [0.85-1.4] | 3.65 [2.84-4.65] | 0.2 [-0.75 to 1.17] |
| Democratic Republic of the Congo | 5.18 [3.79-7.06] | 2.01 [1.38-2.71] | 10.42 [6.55-15.16] | 1.96 [1.16-3.04] | -0.06 [-1.33 to 1.21] |
| Denmark | 5.82 [5.49-6.07] | 7.51 [7.14-7.81] | 6.46 [5.77-7.04] | 5.29 [4.78-5.75] | -1.36 [-2.29 to -0.42] |
| Equatorial Guinea | 0.07 [0.05-0.09] | 2.28 [1.62-2.94] | 0.16 [0.08-0.27] | 1.95 [1.07-3.2] | -0.72 [-2 to 0.58] |
| Fiji | 0.29 [0.16-0.39] | 5.76 [3.19-7.69] | 0.39 [0.22-0.54] | 5.08 [2.82-6.97] | -1.2 [-3.24 to 0.89] |
| Dominica | 0.04 [0.03-0.04] | 5.53 [4.63-6.61] | 0.04 [0.03-0.05] | 5.77 [4.37-7.21] | 0.22 [-0.58 to 1.02] |
| Dominican Republic | 2.21 [1.84-2.7] | 3.7 [3.04-4.78] | 3.3 [2.51-4.39] | 3.18 [2.41-4.26] | -0.46 [-1.32 to 0.42] |
| Papua New Guinea | 1.03 [0.52-1.45] | 3.64 [1.88-5.46] | 2.44 [1.4-3.56] | 3.22 [1.8-5.03] | -1.54 [-3.26 to 0.21] |
| Comoros | 0.09 [0.06-0.12] | 2.5 [1.83-3.37] | 0.15 [0.1-0.21] | 2.63 [1.81-3.66] | -0.04 [-0.95 to 0.89] |
| Georgia | 2.81 [2.61-3.04] | 4.87 [4.52-5.25] | 2.34 [2.11-2.6] | 4.56 [4.11-5.07] | -0.73 [-1.58 to 0.13] |
| Kazakhstan | 6.4 [6.01-6.78] | 4.32 [4.04-4.6] | 4.98 [4.42-5.64] | 2.73 [2.42-3.08] | -1.25 [-2.11 to -0.38] |
| Turkmenistan | 1.15 [1.06-1.24] | 3.55 [3.31-3.8] | 1.34 [1.07-1.69] | 2.78 [2.21-3.51] | -1.28 [-2.21 to -0.34] |
| Bulgaria | 4.86 [4.42-5.31] | 4.72 [4.31-5.14] | 5.84 [4.8-7.01] | 4.67 [3.82-5.62] | -0.28 [-1.1 to 0.54] |
| Greece | 10.27 [9.72-10.73] | 7.24 [6.86-7.54] | 15.75 [14.24-16.93] | 6.25 [5.77-6.65] | -0.81 [-1.62 to 0] |
| Iceland | 0.14 [0.13-0.15] | 4.81 [4.38-5.19] | 0.23 [0.2-0.25] | 3.93 [3.46-4.36] | -1.01 [-1.96 to -0.06] |
| Colombia | 13.62 [12.89-14.32] | 5.25 [4.99-5.5] | 22.23 [18.77-26.15] | 4.27 [3.6-5.04] | -0.84 [-1.7 to 0.02] |
| Honduras | 2.21 [1.81-2.71] | 5.51 [4.42-6.7] | 3.73 [2.63-5.11] | 4.9 [3.59-6.49] | 0.09 [-0.82 to 1.01] |
| Luxembourg | 0.41 [0.38-0.43] | 8 [7.56-8.41] | 0.57 [0.51-0.63] | 5.31 [4.75-5.9] | -2.16 [-3.13 to -1.17] |
| Spain | 28.88 [27.33-30.15] | 5.81 [5.52-6.05] | 38.56 [33.12-42.74] | 3.87 [3.45-4.23] | -1.53 [-2.32 to -0.72] |
| Morocco | 2.49 [1.86-3.17] | 1.33 [0.98-1.65] | 3.85 [2.68-4.94] | 1.15 [0.81-1.46] | -0.72 [-1.56 to 0.14] |
| Syrian Arab Republic | 7 [5.29-8.75] | 8.56 [6.26-10.73] | 7.87 [5.72-10.87] | 6.31 [4.64-8.61] | -0.73 [-1.59 to 0.14] |
| Democratic People's Republic of Korea | 9.19 [6.4-12.59] | 4.79 [3.37-6.47] | 12.01 [8.69-16.9] | 4.07 [2.94-5.65] | -0.36 [-1.36 to 0.65] |
| Yemen | 5.57 [3.34-8.87] | 6.5 [3.97-9.56] | 11.31 [6.24-16.5] | 5.78 [3.21-8.37] | -0.63 [-1.44 to 0.18] |
| Lao People's Democratic Republic | 2.31 [1.26-3.53] | 7.28 [4.49-10.54] | 2.93 [2.12-3.94] | 5.09 [3.7-6.71] | -1.23 [-2.28 to -0.18] |
| Bhutan | 0.15 [0.09-0.21] | 3.5 [2.34-4.81] | 0.18 [0.12-0.28] | 2.87 [1.93-4.29] | -0.43 [-1.71 to 0.88] |
| Mauritania | 0.18 [0.14-0.22] | 1.06 [0.85-1.31] | 0.37 [0.19-0.55] | 1.19 [0.62-1.68] | 0.27 [-0.92 to 1.46] |
| Czechia | 9.2 [8.66-9.77] | 7.01 [6.6-7.46] | 10.95 [9.4-12.64] | 5.11 [4.37-5.88] | -1.13 [-1.96 to -0.29] |
| Cambodia | 5.4 [3.42-7.91] | 7.26 [5.02-9.88] | 8.17 [5.94-10.8] | 5.89 [4.43-7.62] | -0.75 [-1.76 to 0.27] |
| Maldives | 0.08 [0.04-0.12] | 5.11 [3.22-7.32] | 0.09 [0.07-0.12] | 2.44 [1.78-3.15] | -2.63 [-3.77 to -1.46] |
| Antigua and Barbuda | 0.03 [0.02-0.03] | 4.37 [4.05-4.7] | 0.04 [0.03-0.04] | 3.82 [3.58-4.05] | -0.33 [-1.39 to 0.75] |
| Saudi Arabia | 3.07 [2.13-4.95] | 3.2 [2.16-5.56] | 8.73 [6.19-13.54] | 3.61 [2.72-5.29] | 0.79 [0.11 to 1.47] |
| Cuba | 5.28 [5.04-5.48] | 5.06 [4.83-5.27] | 6.5 [5.62-7.36] | 3.77 [3.28-4.28] | -0.94 [-1.84 to -0.04] |
| United Arab Emirates | 0.61 [0.43-0.82] | 8.65 [5.8-11.93] | 2.25 [1.56-3.03] | 5.65 [4.26-7.38] | -0.43 [-1.26 to 0.4] |
| Bangladesh | 35.01 [22.93-49.23] | 4.09 [2.96-5.24] | 39.24 [26.84-53.68] | 2.72 [1.87-3.7] | -1.13 [-2.32 to 0.07] |
| Nigeria | 9.3 [6.51-13.09] | 1.15 [0.82-1.52] | 19.61 [10.75-26.63] | 1.07 [0.64-1.4] | -0.14 [-1.27 to 1] |
| Togo | 0.3 [0.22-0.39] | 1.03 [0.79-1.26] | 0.78 [0.37-1.14] | 1.34 [0.66-1.89] | 0.93 [-0.17 to 2.04] |
| United States Virgin Islands | 0.04 [0.03-0.05] | 4.23 [3.32-5.17] | 0.04 [0.03-0.05] | 2.61 [1.89-3.41] | -1.2 [-2.25 to -0.14] |
| Azerbaijan | 3.1 [2.57-3.8] | 4.63 [3.84-5.63] | 3.37 [2.38-4.99] | 3.32 [2.36-4.86] | -0.94 [-1.89 to 0.03] |
| Greenland | 0.02 [0.01-0.02] | 4.01 [3.17-4.84] | 0.02 [0.01-0.02] | 2.49 [1.88-3.3] | -1.4 [-2.32 to -0.48] |
| Nauru | 0 [0-0] | 5.63 [4.09-7.47] | 0 [0-0.01] | 5.16 [3.45-7.38] | -1.23 [-2.89 to 0.45] |
| Tajikistan | 1.88 [1.41-2.3] | 3.86 [2.89-4.74] | 2.24 [1.45-3.39] | 2.48 [1.67-3.59] | -1.57 [-2.54 to -0.59] |
| Hungary | 9.31 [8.73-9.94] | 6.85 [6.41-7.33] | 8.91 [7.56-10.56] | 4.86 [4.11-5.8] | -1.03 [-1.86 to -0.19] |
| Benin | 0.5 [0.38-0.67] | 1.1 [0.89-1.37] | 1.37 [0.66-1.96] | 1.3 [0.65-1.82] | 0.49 [-0.63 to 1.62] |
| Bosnia and Herzegovina | 1.49 [1.27-1.8] | 3.67 [3.14-4.49] | 2.05 [1.5-2.6] | 3.52 [2.59-4.46] | -0.5 [-1.41 to 0.41] |
| Saint Kitts and Nevis | 0.02 [0.02-0.02] | 5.2 [4.88-5.55] | 0.02 [0.02-0.02] | 3.49 [2.96-4.02] | -0.79 [-1.7 to 0.14] |
| Serbia | 5.22 [4.24-6.2] | 5.29 [4.36-6.38] | 6.67 [5.05-8.21] | 4.2 [3.16-5.15] | -1.05 [-1.94 to -0.16] |
| Chad | 0.53 [0.39-0.72] | 0.94 [0.72-1.2] | 1.94 [1.01-2.85] | 1.4 [0.75-1.96] | 1.34 [0.27 to 2.42] |
| Philippines | 24.51 [20.16-29.68] | 5.15 [4.07-6.07] | 39.91 [33.64-47.58] | 4.19 [3.53-5.04] | -0.67 [-1.78 to 0.47] |
| Estonia | 1.35 [1.26-1.43] | 7.19 [6.73-7.64] | 1.15 [0.98-1.32] | 4.51 [3.85-5.14] | -1.63 [-2.48 to -0.77] |
| Guinea-Bissau | 0.12 [0.08-0.17] | 1.4 [1.01-1.93] | 0.22 [0.12-0.3] | 1.62 [0.91-2.18] | 0.58 [-0.45 to 1.63] |
| Russian Federation | 75.68 [74.18-77.27] | 4.55 [4.45-4.65] | 80.7 [74.76-86.76] | 3.69 [3.44-3.96] | -0.7 [-1.45 to 0.06] |
| Ukraine | 37.95 [36.11-39.76] | 6.24 [5.94-6.53] | 23.44 [17.53-30.22] | 3.53 [2.66-4.51] | -1.48 [-2.21 to -0.75] |
| Viet Nam | 16.8 [13.17-21.65] | 3.35 [2.64-4.32] | 24.93 [18.78-34.21] | 2.56 [1.95-3.53] | -0.95 [-2.56 to 0.68] |
| Micronesia (Federated States of) | 0.03 [0.02-0.05] | 5.09 [3.63-6.89] | 0.04 [0.02-0.05] | 4.39 [2.99-5.97] | -1.46 [-3.09 to 0.2] |
| Vanuatu | 0.03 [0.02-0.05] | 3.74 [2.24-5.17] | 0.08 [0.05-0.1] | 3.59 [2.38-4.68] | -1.31 [-2.95 to 0.36] |
| Kenya | 2.62 [1.95-3.45] | 1.82 [1.33-2.69] | 6.3 [4.49-8.53] | 2.11 [1.56-2.83] | 0.53 [-0.28 to 1.35] |
| Mozambique | 4.56 [3.41-6.01] | 3.89 [3.14-4.78] | 7.32 [4.57-11.37] | 3.75 [2.73-5.09] | 0.01 [-0.89 to 0.91] |
| Rwanda | 1.72 [1.26-2.3] | 3.18 [2.48-4.18] | 2.26 [1.39-3.45] | 2.58 [1.69-3.8] | -0.65 [-1.48 to 0.2] |
| China | 674.23 [520.45-800.43] | 6.46 [5.04-7.66] | 589.03 [436.26-740.39] | 3.42 [2.51-4.26] | -2.04 [-3.05 to -1.01] |
| Venezuela (Bolivarian Republic of) | 6.9 [6.66-7.14] | 4.5 [4.33-4.66] | 11.73 [8.74-15.25] | 4.26 [3.18-5.54] | -0.45 [-1.35 to 0.45] |
| Uganda | 2.34 [1.65-3.04] | 1.8 [1.3-2.3] | 5.34 [3.1-7.98] | 1.87 [1.23-2.56] | 0.16 [-0.68 to 1.02] |
| Indonesia | 70.86 [53.12-91.98] | 5.01 [3.83-6.35] | 113.4 [91-146.16] | 4.65 [3.78-6.14] | 0 [-1.08 to 1.09] |
| Haiti | 3.99 [2.09-6.26] | 7.49 [4.73-10.58] | 5.88 [3.65-8.74] | 5.93 [3.93-8.4] | -0.53 [-1.28 to 0.21] |
| Namibia | 0.2 [0.15-0.25] | 2.26 [1.77-2.88] | 0.37 [0.26-0.52] | 2.26 [1.63-3.07] | -0.18 [-1.85 to 1.52] |
| Myanmar | 24.02 [14.06-35.56] | 7.39 [4.78-10.44] | 24.73 [18.54-32.58] | 4.87 [3.72-6.42] | -1.38 [-2.45 to -0.3] |
| Iran (Islamic Republic of) | 30.41 [18.95-38.1] | 7.54 [4.71-9.26] | 38.84 [25.34-44.88] | 5.06 [3.3-5.85] | -1.16 [-1.95 to -0.37] |
| Germany | 70.74 [66.66-74.01] | 5.96 [5.66-6.21] | 94.95 [83.81-103.79] | 4.71 [4.25-5.07] | -1.07 [-2.03 to -0.1] |
| Italy | 54.02 [50.99-55.84] | 6.69 [6.36-6.89] | 71.41 [62.49-77.21] | 4.76 [4.3-5.08] | -1.6 [-2.41 to -0.79] |
| Portugal | 7.18 [6.8-7.53] | 5.84 [5.53-6.1] | 10.05 [8.79-11.03] | 4.06 [3.63-4.41] | -1.53 [-2.44 to -0.61] |
| United Kingdom | 43.4 [41.42-44.37] | 5.12 [4.91-5.22] | 57.32 [51.77-60.15] | 4.33 [3.99-4.51] | -0.92 [-2.01 to 0.18] |
| Japan | 63.77 [61.37-65.09] | 4.2 [4.04-4.28] | 105.92 [91.26-114.21] | 3.06 [2.79-3.21] | -1.09 [-2.31 to 0.15] |
| New Zealand | 2.34 [2.2-2.46] | 6.11 [5.77-6.39] | 3.62 [3.27-3.87] | 4.37 [3.97-4.65] | -0.67 [-1.71 to 0.39] |
| Belgium | 9.9 [9.15-10.63] | 6.81 [6.33-7.28] | 11.82 [10.16-13.13] | 4.94 [4.35-5.42] | -1.82 [-2.67 to -0.96] |
| Trinidad and Tobago | 0.47 [0.45-0.49] | 4.84 [4.61-5.06] | 0.67 [0.51-0.84] | 3.91 [3.03-4.92] | -1.14 [-1.92 to -0.34] |
| Cyprus | 0.54 [0.43-0.74] | 7.85 [6.16-11.27] | 0.98 [0.7-1.25] | 5.07 [3.61-6.32] | -1.48 [-2.69 to -0.27] |
| Peru | 9.12 [7.52-11.79] | 5.05 [4.2-6.49] | 15.53 [9.9-20.57] | 4.49 [2.87-5.95] | -0.17 [-1.21 to 0.88] |
| Congo | 0.37 [0.28-0.5] | 2.42 [1.88-3.06] | 0.76 [0.49-1.07] | 2.19 [1.41-3.09] | -0.35 [-1.63 to 0.94] |
| Guatemala | 2.65 [2.49-2.8] | 3.78 [3.63-3.92] | 5.67 [4.91-6.54] | 4.09 [3.56-4.7] | -0.01 [-1.03 to 1.03] |
| Burundi | 1.19 [0.88-1.61] | 2.75 [2.08-3.61] | 2.05 [1.14-3.2] | 2.5 [1.51-3.72] | -0.28 [-1.11 to 0.55] |

| **Country** | **1990** | | **2021** | | **1990-2021** |
| --- | --- | --- | --- | --- | --- |
|  | **YLDs cases** | **YLDs per 100,000** | **YLDs cases** | **YLDs per 100,000** | **EAPC** |
|  | **No. *10^2^ (95% UI)** | **No. (95% UI)** | **No. *10^2^ (95% UI)** | **No.(95% UI)** | **No. (95% CI)** |
| China | 289.12 [191.52-402.08] | 2.73 [1.81-3.79] | 607.76 [378.58-861.8] | 4.16 [2.55-6.06] | 0.84 [-1.01 to 2.73] |
| Indonesia | 23.65 [14.17-35.27] | 1.63 [1-2.44] | 42.26 [27.64-60.82] | 1.66 [1.07-2.47] | 0.04 [-1.41 to 1.52] |
| Taiwan (Province of China) | 2.12 [1.49-2.82] | 1.18 [0.82-1.56] | 6.93 [4.9-9.42] | 2.28 [1.59-3.12] | 2.32 [1.04 to 3.62] |
| Cambodia | 1.75 [0.88-2.86] | 2.33 [1.28-3.71] | 2.96 [1.93-4.39] | 2.05 [1.36-3.04] | -0.43 [-1.79 to 0.94] |
| Lao People's Democratic Republic | 0.74 [0.35-1.24] | 2.32 [1.18-3.73] | 1.02 [0.64-1.59] | 1.71 [1.06-2.68] | -1.14 [-2.56 to 0.3] |
| Democratic People's Republic of Korea | 3.6 [2.25-5.35] | 1.83 [1.15-2.69] | 5.83 [3.55-9.05] | 2.01 [1.23-3.09] | 0.29 [-1.16 to 1.76] |
| Philippines | 7.98 [5.38-11.19] | 1.58 [1.04-2.15] | 13.84 [9.22-18.94] | 1.39 [0.92-1.9] | -0.55 [-1.85 to 0.77] |
| Fiji | 0.08 [0.04-0.13] | 1.5 [0.76-2.26] | 0.11 [0.06-0.17] | 1.36 [0.72-2.06] | -1.38 [-3.99 to 1.3] |
| Thailand | 8.87 [5.75-12.35] | 2.03 [1.28-2.84] | 18.88 [11.77-27.28] | 2.28 [1.44-3.26] | -0.02 [-1.18 to 1.16] |
| Viet Nam | 6.11 [4.1-8.79] | 1.18 [0.8-1.73] | 11.39 [7.29-17.19] | 1.17 [0.75-1.77] | 0.08 [-2.19 to 2.42] |
| Maldives | 0.03 [0.01-0.04] | 1.72 [0.9-2.83] | 0.05 [0.03-0.07] | 1.19 [0.76-1.71] | -1.29 [-2.89 to 0.34] |
| Marshall Islands | 0 [0-0.01] | 1.28 [0.76-1.9] | 0.01 [0-0.01] | 1.28 [0.77-1.89] | -1.49 [-3.94 to 1.02] |
| Malaysia | 2.79 [1.92-3.85] | 2.1 [1.46-3.01] | 6.34 [4.29-9.62] | 2.12 [1.43-3.26] | 0.19 [-1.48 to 1.9] |
| Kazakhstan | 2.79 [1.94-3.66] | 1.89 [1.31-2.47] | 2.95 [2.1-4.06] | 1.58 [1.12-2.17] | -0.21 [-1.54 to 1.13] |
| Papua New Guinea | 0.32 [0.15-0.53] | 1.09 [0.5-1.9] | 0.77 [0.41-1.25] | 0.97 [0.5-1.66] | -2.02 [-4.57 to 0.59] |
| Timor-Leste | 0.1 [0.05-0.15] | 1.68 [0.99-2.45] | 0.17 [0.1-0.25] | 1.5 [0.94-2.26] | -0.25 [-1.7 to 1.21] |
| Solomon Islands | 0.03 [0.01-0.05] | 1.33 [0.56-2.19] | 0.06 [0.04-0.1] | 1.29 [0.72-1.97] | -1.65 [-4.11 to 0.87] |
| Mongolia | 0.2 [0.13-0.28] | 1.09 [0.73-1.55] | 0.28 [0.18-0.4] | 0.94 [0.6-1.36] | -0.48 [-1.92 to 0.97] |
| Vanuatu | 0.01 [0.01-0.02] | 1.16 [0.62-1.81] | 0.03 [0.02-0.04] | 1.1 [0.66-1.64] | -1.79 [-4.24 to 0.72] |
| Myanmar | 7.87 [3.62-13.15] | 2.39 [1.2-3.9] | 8.87 [5.62-13.8] | 1.7 [1.08-2.68] | -1.46 [-2.93 to 0.02] |
| Sri Lanka | 2.67 [1.76-3.68] | 1.94 [1.28-2.67] | 3.64 [2.15-5.95] | 1.49 [0.9-2.41] | 0.39 [-1.51 to 2.32] |
| Turkmenistan | 0.45 [0.32-0.59] | 1.44 [1-1.9] | 0.62 [0.42-0.88] | 1.29 [0.86-1.79] | -0.54 [-1.9 to 0.84] |
| Kiribati | 0.01 [0-0.01] | 0.94 [0.56-1.38] | 0.01 [0-0.01] | 0.93 [0.5-1.42] | -1.48 [-3.99 to 1.1] |
| Armenia | 0.84 [0.6-1.1] | 2.65 [1.9-3.49] | 0.93 [0.66-1.25] | 2.43 [1.73-3.23] | -0.16 [-1.54 to 1.23] |
| Azerbaijan | 1.25 [0.85-1.74] | 1.88 [1.29-2.61] | 1.74 [1.04-2.65] | 1.7 [1.03-2.57] | -0.47 [-1.88 to 0.95] |
| Samoa | 0.02 [0.01-0.03] | 1.74 [1.11-2.65] | 0.03 [0.02-0.04] | 1.59 [1.02-2.45] | -1.63 [-3.85 to 0.65] |
| Tonga | 0.01 [0-0.01] | 1.03 [0.61-1.59] | 0.01 [0.01-0.01] | 0.99 [0.63-1.52] | -1.63 [-3.84 to 0.64] |
| Tajikistan | 0.72 [0.47-1.03] | 1.53 [0.99-2.2] | 0.95 [0.55-1.59] | 1.05 [0.62-1.66] | -1.69 [-3.14 to -0.23] |
| Micronesia (Federated States of) | 0.01 [0.01-0.02] | 1.53 [0.95-2.28] | 0.01 [0.01-0.02] | 1.35 [0.86-2.03] | -1.78 [-4.15 to 0.65] |
| Kyrgyzstan | 0.69 [0.49-0.9] | 1.76 [1.24-2.29] | 0.75 [0.51-1.04] | 1.23 [0.85-1.71] | -1.24 [-2.52 to 0.05] |
| Croatia | 2.12 [1.49-2.86] | 3.66 [2.58-4.95] | 4.62 [3.27-6.14] | 5.61 [3.95-7.5] | 0.46 [-1.22 to 2.16] |
| Hungary | 4.94 [3.48-6.45] | 3.55 [2.52-4.57] | 8.01 [5.7-10.98] | 4.56 [3.27-6.33] | -0.09 [-1.62 to 1.46] |
| Poland | 13.28 [9.35-17.56] | 3.12 [2.19-4.12] | 28.99 [20.88-38.14] | 4.29 [3.11-5.67] | -0.12 [-1.69 to 1.48] |
| Romania | 5.07 [3.58-6.78] | 1.98 [1.39-2.64] | 9.85 [6.74-13.36] | 3.03 [2.08-4.14] | 0.89 [-0.67 to 2.47] |
| Slovakia | 1.91 [1.31-2.68] | 3.25 [2.25-4.58] | 3.87 [2.3-5.58] | 4.34 [2.63-6.21] | 0.19 [-1.78 to 2.2] |
| Ukraine | 21.94 [15.54-29] | 3.56 [2.53-4.68] | 17.83 [11.38-26.22] | 2.74 [1.78-3.94] | -1.33 [-2.64 to 0] |
| North Macedonia | 0.39 [0.25-0.53] | 2.06 [1.34-2.85] | 1 [0.57-1.5] | 3.12 [1.81-4.67] | 0.53 [-7.09 to 8.77] |
| Georgia | 1.6 [1.15-2.14] | 2.72 [1.95-3.64] | 1.35 [0.96-1.81] | 2.63 [1.86-3.51] | -0.33 [-1.84 to 1.2] |
| Russian Federation | 43.31 [30.86-56.64] | 2.55 [1.82-3.34] | 71.59 [51.71-93.53] | 3.35 [2.44-4.37] | 0.25 [-1.18 to 1.7] |
| Bosnia and Herzegovina | 0.68 [0.45-0.95] | 1.62 [1.1-2.29] | 1.6 [0.93-2.31] | 2.75 [1.63-3.96] | 1.39 [-0.27 to 3.08] |
| Latvia | 1.39 [0.98-1.82] | 4.2 [2.95-5.49] | 1.64 [1.16-2.22] | 4.64 [3.3-6.28] | -0.69 [-2.34 to 1] |
| Uzbekistan | 3.27 [2.35-4.39] | 1.9 [1.35-2.54] | 4.49 [3.08-6.39] | 1.39 [0.96-1.97] | -1.13 [-2.56 to 0.32] |
| Lithuania | 2.03 [1.46-2.66] | 4.76 [3.41-6.23] | 2.11 [1.51-2.88] | 4.17 [3-5.72] | -0.71 [-2.28 to 0.87] |
| Albania | 0.4 [0.26-0.56] | 1.57 [1.02-2.26] | 0.79 [0.44-1.44] | 2.17 [1.28-3.74] | 1.14 [-0.43 to 2.73] |
| Bulgaria | 2.39 [1.69-3.22] | 2.24 [1.58-2.99] | 3.27 [2.28-4.64] | 2.64 [1.82-3.76] | 0.32 [-1.07 to 1.74] |
| Montenegro | 0.19 [0.12-0.27] | 3.04 [1.91-4.33] | 0.37 [0.22-0.57] | 3.95 [2.37-6.03] | 0.63 [-1.1 to 2.39] |
| Estonia | 0.99 [0.72-1.31] | 5.15 [3.72-6.8] | 1.31 [0.91-1.82] | 5.39 [3.79-7.52] | -0.34 [-2.02 to 1.37] |
| Czechia | 4.96 [3.55-6.49] | 3.81 [2.76-4.94] | 10.9 [7.82-14.46] | 5.32 [3.8-7.13] | 0.2 [-1.48 to 1.91] |
| Serbia | 2.52 [1.69-3.47] | 2.45 [1.66-3.37] | 5.67 [3.49-8.12] | 3.62 [2.24-5.16] | 0.38 [-1.29 to 2.08] |
| Slovenia | 0.93 [0.66-1.21] | 3.95 [2.82-5.18] | 3.04 [2.1-4.23] | 7.31 [5.02-10.19] | 1.28 [-0.49 to 3.09] |
| Belarus | 4.65 [3.37-6.21] | 3.84 [2.78-5.12] | 6.97 [4.81-9.92] | 4.73 [3.32-6.7] | 0.17 [-1.52 to 1.88] |
| Japan | 38.21 [27.27-50.85] | 2.98 [2.13-4.02] | 65.43 [46.28-85.62] | 2.97 [2.15-3.94] | -0.49 [-1.91 to 0.94] |
| Republic of Korea | 6.52 [4.47-9.03] | 1.71 [1.17-2.43] | 12.74 [7.83-18.21] | 2.26 [1.29-3.35] | 0.81 [-0.58 to 2.21] |
| Andorra | 0.04 [0.02-0.07] | 7.98 [4.64-13.17] | 0.08 [0.05-0.14] | 6.81 [4.08-11.34] | -0.51 [-2.25 to 1.25] |
| France | 38.57 [27.87-51.14] | 5.26 [3.8-6.96] | 71.13 [51.82-96.29] | 6.01 [4.39-8.1] | 0.47 [-0.96 to 1.92] |
| Denmark | 4.19 [3.03-5.49] | 5.79 [4.17-7.58] | 6.87 [4.82-9.03] | 6.53 [4.59-8.63] | 0.23 [-1.34 to 1.83] |
| Belgium | 7.57 [5.46-10] | 5.8 [4.14-7.62] | 10.2 [7.51-13.37] | 5.39 [3.96-7.18] | -0.53 [-1.94 to 0.9] |
| Greece | 9.08 [6.63-11.87] | 7 [4.95-9.18] | 12.44 [8.93-16.4] | 6.44 [4.68-8.61] | -0.71 [-2.05 to 0.66] |
| Germany | 55.06 [39.82-71.08] | 5.02 [3.62-6.44] | 97.59 [70.05-130.97] | 6.07 [4.39-8.11] | 0.12 [-1.39 to 1.65] |
| Cyprus | 0.3 [0.2-0.47] | 3.93 [2.59-6.24] | 1.17 [0.67-1.68] | 6.24 [3.67-8.99] | 1.71 [-0.32 to 3.77] |
| Finland | 2.5 [1.78-3.29] | 3.81 [2.74-5.06] | 4.7 [3.38-6.18] | 4.56 [3.27-6.09] | 0.46 [-1.11 to 2.05] |
| Brunei Darussalam | 0.04 [0.03-0.05] | 2.31 [1.53-3.2] | 0.08 [0.05-0.11] | 1.97 [1.27-2.75] | -0.22 [-1.65 to 1.23] |
| Ireland | 2.07 [1.47-2.72] | 5.13 [3.67-6.78] | 3.43 [2.46-4.56] | 4.84 [3.44-6.45] | -0.01 [-1.66 to 1.66] |
| Malta | 0.13 [0.09-0.18] | 3.39 [2.39-4.54] | 0.28 [0.19-0.38] | 4.23 [2.95-5.94] | 0.25 [-1.16 to 1.67] |
| Norway | 2.15 [1.55-2.76] | 3.69 [2.68-4.8] | 4.12 [2.98-5.35] | 4.72 [3.46-6.13] | 0.47 [-1.17 to 2.14] |
| Spain | 24.81 [18.23-32.68] | 5.44 [3.95-7.32] | 41.92 [30.1-55.75] | 5.65 [4.13-7.64] | -0.31 [-1.87 to 1.28] |
| Luxembourg | 0.28 [0.2-0.37] | 5.86 [4.2-7.75] | 0.53 [0.39-0.74] | 5.68 [4.13-7.79] | -0.47 [-1.99 to 1.08] |
| Switzerland | 5.98 [4.27-7.89] | 7.2 [5.11-9.5] | 8.62 [6.13-11.43] | 5.87 [4.21-7.8] | -0.71 [-2.27 to 0.88] |
| Portugal | 4.12 [2.92-5.44] | 3.41 [2.42-4.51] | 8.55 [6.12-11.47] | 4.48 [3.23-5.96] | 0.56 [-0.94 to 2.08] |
| United Kingdom | 48.25 [35.24-62.66] | 6.14 [4.53-8] | 55.8 [40.59-71.74] | 4.97 [3.68-6.44] | -0.4 [-2.14 to 1.38] |
| Israel | 2.56 [1.86-3.31] | 5.15 [3.77-6.67] | 6.66 [4.83-8.85] | 5.6 [4.07-7.5] | 0.21 [-1.59 to 2.04] |
| Republic of Moldova | 0.99 [0.69-1.31] | 2.23 [1.55-2.93] | 0.87 [0.62-1.19] | 1.74 [1.23-2.39] | -0.77 [-2.29 to 0.78] |
| Netherlands | 9.77 [7.03-12.92] | 5.56 [4.02-7.31] | 13.12 [9.28-17.8] | 4.4 [3.11-5.96] | -0.37 [-1.91 to 1.19] |
| Chile | 2.1 [1.49-2.75] | 1.84 [1.3-2.42] | 4.85 [3.51-6.37] | 2.22 [1.59-2.96] | 0.64 [-0.5 to 1.81] |
| Sweden | 7.07 [5.09-9.05] | 5.88 [4.21-7.66] | 9.03 [6.57-12.24] | 4.9 [3.58-6.59] | -0.19 [-1.96 to 1.62] |
| Argentina | 7.4 [5.22-9.65] | 2.27 [1.6-2.96] | 11.06 [8.01-14.88] | 2.16 [1.56-2.9] | -0.45 [-1.58 to 0.69] |
| Uruguay | 1.04 [0.73-1.34] | 2.82 [2-3.67] | 1.72 [1.24-2.28] | 3.48 [2.52-4.58] | 0.12 [-1.2 to 1.46] |
| New Zealand | 2.25 [1.65-2.93] | 5.81 [4.27-7.58] | 4.92 [3.55-6.48] | 6.29 [4.56-8.28] | 0.04 [-1.55 to 1.65] |
| Canada | 21.81 [16.12-28.53] | 7.15 [5.28-9.38] | 35.09 [25.45-45.98] | 5.59 [4.13-7.37] | -0.5 [-2.28 to 1.31] |
| United States of America | 214.94 [157.31-276.72] | 7.08 [5.21-9.11] | 268.48 [197.6-343.13] | 5.09 [3.75-6.53] | -1 [-2.66 to 0.67] |
| Belize | 0.02 [0.02-0.03] | 1.37 [0.99-1.83] | 0.05 [0.04-0.07] | 1.55 [1.1-2.13] | 0.52 [-0.95 to 2.02] |
| Bahamas | 0.03 [0.02-0.04] | 1.59 [1.12-2.13] | 0.07 [0.05-0.09] | 1.67 [1.13-2.31] | 0.05 [-1.12 to 1.25] |
| Dominica | 0.01 [0.01-0.02] | 1.91 [1.33-2.66] | 0.02 [0.01-0.02] | 2.28 [1.48-3.32] | 0.48 [-0.63 to 1.61] |
| Antigua and Barbuda | 0.01 [0.01-0.01] | 1.53 [1.1-2.01] | 0.02 [0.01-0.02] | 1.61 [1.14-2.14] | 0.04 [-1.22 to 1.31] |
| Italy | 50.49 [36.48-65.33] | 7.15 [5.29-9.34] | 71.49 [52.46-92.99] | 6.41 [4.69-8.54] | -0.88 [-2.48 to 0.75] |
| Australia | 10.06 [7.28-13.11] | 5.32 [3.88-6.93] | 21.99 [15.82-28.68] | 5.39 [3.87-7.15] | -0.31 [-1.84 to 1.25] |
| Cuba | 2.33 [1.64-3.06] | 2.25 [1.58-2.95] | 3.73 [2.66-4.98] | 2.31 [1.66-3.1] | 0.03 [-1.22 to 1.3] |
| Singapore | 0.48 [0.33-0.63] | 1.92 [1.34-2.53] | 1.37 [0.98-1.82] | 2.44 [1.72-3.31] | 0.51 [-1.12 to 2.17] |
| Austria | 5.3 [3.87-6.93] | 5.1 [3.69-6.72] | 8.85 [6.34-11.44] | 5.54 [4-7.27] | 0.17 [-1.39 to 1.75] |
| Iceland | 0.14 [0.1-0.18] | 5.1 [3.6-6.64] | 0.25 [0.18-0.34] | 5.17 [3.65-7.04] | -0.01 [-1.58 to 1.59] |
| Barbados | 0.07 [0.05-0.09] | 2.34 [1.68-3.08] | 0.11 [0.07-0.15] | 2.42 [1.59-3.48] | 0.44 [-0.98 to 1.87] |
| Grenada | 0.02 [0.01-0.02] | 2.18 [1.52-2.82] | 0.03 [0.02-0.04] | 2.41 [1.73-3.27] | 0.28 [-1.09 to 1.68] |
| Suriname | 0.05 [0.03-0.06] | 1.45 [0.95-1.97] | 0.09 [0.06-0.12] | 1.43 [0.95-2.06] | 0.01 [-1.18 to 1.21] |
| Saint Lucia | 0.02 [0.01-0.03] | 1.93 [1.34-2.53] | 0.04 [0.03-0.05] | 1.73 [1.19-2.44] | -0.72 [-1.84 to 0.43] |
| Haiti | 1.35 [0.64-2.26] | 2.57 [1.44-3.99] | 2.13 [1.2-3.44] | 2.12 [1.26-3.33] | -0.44 [-1.6 to 0.74] |
| Ecuador | 1.35 [0.96-1.76] | 1.73 [1.22-2.26] | 3.71 [2.52-5.18] | 2.15 [1.47-3.01] | 1.15 [-0.29 to 2.62] |
| Dominican Republic | 0.85 [0.58-1.19] | 1.4 [0.95-1.96] | 1.45 [0.93-2.14] | 1.39 [0.89-2.04] | 0.36 [-1.08 to 1.82] |
| Trinidad and Tobago | 0.17 [0.13-0.23] | 1.76 [1.25-2.3] | 0.27 [0.18-0.39] | 1.62 [1.07-2.34] | -0.33 [-1.39 to 0.75] |
| Colombia | 5.09 [3.61-6.69] | 1.97 [1.38-2.58] | 10.99 [7.59-15.05] | 2.2 [1.52-3.04] | 0.19 [-1.05 to 1.45] |
| Saint Vincent and the Grenadines | 0.02 [0.01-0.03] | 2.38 [1.71-3.1] | 0.03 [0.02-0.04] | 2.19 [1.54-2.97] | -0.21 [-1.56 to 1.16] |
| Peru | 3.36 [2.21-4.71] | 1.86 [1.21-2.57] | 8.24 [5-12.08] | 2.38 [1.44-3.5] | 1.21 [-0.35 to 2.79] |
| Bolivia (Plurinational State of) | 1.41 [0.84-2.12] | 2.73 [1.67-4.02] | 2.5 [1.54-3.89] | 2.42 [1.5-3.77] | -0.27 [-1.7 to 1.19] |
| Jamaica | 0.36 [0.26-0.48] | 1.73 [1.24-2.28] | 0.58 [0.39-0.83] | 1.97 [1.31-2.81] | 0.9 [-0.74 to 2.56] |
| Nicaragua | 0.51 [0.34-0.72] | 1.44 [0.98-1.99] | 0.86 [0.57-1.22] | 1.48 [0.98-2.1] | 0.41 [-0.87 to 1.71] |
| Mexico | 12.27 [8.8-16.19] | 1.71 [1.21-2.22] | 24.22 [16.67-32.75] | 1.92 [1.33-2.61] | 0.04 [-1.36 to 1.45] |
| Guyana | 0.07 [0.05-0.09] | 1.21 [0.84-1.6] | 0.09 [0.06-0.13] | 1.26 [0.83-1.82] | 0.54 [-0.69 to 1.79] |
| El Salvador | 0.87 [0.6-1.21] | 1.94 [1.33-2.68] | 1.57 [1.03-2.28] | 2.49 [1.63-3.62] | 0.94 [-0.72 to 2.63] |
| Costa Rica | 0.53 [0.37-0.69] | 2.25 [1.59-2.93] | 1.55 [1.12-2.08] | 3.13 [2.26-4.21] | 0.66 [-0.52 to 1.86] |
| Guatemala | 0.92 [0.65-1.2] | 1.38 [0.96-1.79] | 2.27 [1.6-3.12] | 1.64 [1.15-2.26] | 0.11 [-1.5 to 1.76] |
| Honduras | 0.72 [0.48-1] | 1.82 [1.21-2.52] | 1.36 [0.85-2.08] | 1.76 [1.1-2.62] | 0.14 [-1.09 to 1.39] |
| Panama | 0.32 [0.23-0.42] | 1.54 [1.09-2.04] | 0.97 [0.65-1.39] | 2.28 [1.54-3.29] | 0.83 [-0.44 to 2.12] |
| Venezuela (Bolivarian Republic of) | 2.39 [1.67-3.17] | 1.55 [1.08-2.04] | 5.03 [3.3-7.33] | 1.84 [1.2-2.67] | 0.14 [-1.01 to 1.31] |
| Paraguay | 0.48 [0.32-0.67] | 1.49 [1-2.06] | 1.29 [0.83-1.88] | 2.07 [1.35-3.01] | 1.06 [-0.08 to 2.22] |
| Brazil | 17.9 [12.68-23.33] | 1.56 [1.09-2.04] | 38.96 [27.92-51.24] | 1.64 [1.18-2.17] | -0.12 [-1.27 to 1.04] |
| Algeria | 2.77 [1.79-3.99] | 1.45 [0.96-2.02] | 5.18 [3.28-7.71] | 1.39 [0.89-2.04] | 0.25 [-1.46 to 2] |
| Bahrain | 0.08 [0.05-0.11] | 3.29 [2.12-4.59] | 0.25 [0.16-0.38] | 2.57 [1.62-3.87] | -0.98 [-2.95 to 1.03] |
| Egypt | 6.63 [4.09-11.26] | 1.63 [0.96-2.88] | 21.64 [12.66-32.18] | 2.83 [1.73-4.11] | 2.28 [1.01 to 3.57] |
| Iran (Islamic Republic of) | 12.07 [6.65-17.91] | 2.83 [1.49-4.08] | 23.81 [12.4-34.96] | 3.05 [1.58-4.47] | 0.32 [-0.91 to 1.57] |
| Iraq | 2.95 [1.86-4.74] | 2.24 [1.42-3.6] | 8.27 [5.18-12.25] | 2.71 [1.69-3.96] | 0.63 [-0.4 to 1.67] |
| Lebanon | 0.57 [0.32-0.95] | 2.42 [1.37-3.93] | 1.9 [1.19-2.83] | 3.25 [2.03-4.87] | 0.94 [-0.4 to 2.29] |
| Libya | 0.69 [0.45-0.97] | 2.47 [1.58-3.57] | 1.77 [1.13-2.7] | 3.19 [2.02-4.85] | 1.12 [-0.29 to 2.55] |
| Jordan | 0.58 [0.38-0.82] | 2.48 [1.65-3.5] | 2.27 [1.38-3.45] | 2.56 [1.56-3.91] | 0.37 [-1.05 to 1.8] |
| Kuwait | 0.32 [0.23-0.43] | 2.96 [2.15-3.91] | 0.76 [0.52-1.05] | 2.45 [1.64-3.41] | -0.51 [-2.03 to 1.03] |
| Morocco | 0.86 [0.55-1.24] | 0.44 [0.28-0.63] | 1.66 [1-2.47] | 0.48 [0.29-0.72] | 0.16 [-1.1 to 1.45] |
| Palestine | 0.54 [0.34-0.79] | 4.16 [2.62-5.88] | 1.29 [0.86-1.83] | 3.89 [2.61-5.46] | -0.33 [-2.17 to 1.54] |
| Oman | 0.2 [0.12-0.3] | 1.91 [1.11-2.81] | 0.5 [0.31-0.75] | 2.02 [1.28-2.97] | 0.14 [-1.71 to 2.01] |
| Qatar | 0.06 [0.04-0.09] | 3.55 [2.13-5.15] | 0.46 [0.26-0.74] | 3.56 [2.06-5.4] | -0.29 [-1.85 to 1.3] |
| Saudi Arabia | 1.19 [0.73-1.95] | 1.2 [0.72-2.07] | 5.97 [3.59-10.02] | 2.32 [1.42-3.64] | 2.19 [1.07 to 3.33] |
| Syrian Arab Republic | 2.55 [1.67-3.71] | 2.99 [1.92-4.32] | 4.43 [2.65-6.9] | 3.34 [2.05-5.21] | -0.09 [-1.38 to 1.22] |
| Tunisia | 0.92 [0.6-1.33] | 1.4 [0.92-1.99] | 2.01 [1.1-3.2] | 1.62 [0.91-2.55] | 0.14 [-1.21 to 1.51] |
| United Arab Emirates | 0.25 [0.15-0.36] | 3.33 [2.01-4.92] | 1.3 [0.79-1.97] | 2.86 [1.8-4.19] | 0.19 [-1.3 to 1.69] |
| Yemen | 1.9 [1.01-3.18] | 2.12 [1.16-3.34] | 4.33 [2.22-6.97] | 2.08 [1.07-3.35] | -0.22 [-1.35 to 0.93] |
| Turkey | 13.97 [9.08-20.28] | 3.1 [1.98-4.4] | 28.67 [18.31-41.4] | 3.29 [2.13-4.75] | 0.04 [-1.37 to 1.46] |
| Afghanistan | 2.73 [1.21-4.61] | 3.36 [1.58-5.51] | 5.92 [3.04-9.66] | 3.28 [1.69-5.3] | -0.14 [-1.28 to 1.02] |
| Bangladesh | 13.01 [7.32-20.6] | 1.52 [0.95-2.23] | 16.51 [10.26-25.79] | 1.12 [0.69-1.74] | -0.78 [-2.54 to 1.01] |
| Bhutan | 0.06 [0.03-0.09] | 1.3 [0.74-2] | 0.08 [0.04-0.12] | 1.16 [0.7-1.89] | -0.25 [-2.16 to 1.71] |
| India | 77.56 [52.78-109.29] | 1.19 [0.8-1.64] | 134.99 [89.73-184.74] | 1.08 [0.71-1.48] | -0.21 [-1.81 to 1.41] |
| Congo | 0.12 [0.07-0.18] | 0.79 [0.51-1.13] | 0.3 [0.17-0.46] | 0.84 [0.46-1.28] | -0.21 [-2.07 to 1.69] |
| Nepal | 1.98 [1.13-3.19] | 1.31 [0.84-1.93] | 2.96 [1.9-4.55] | 1.14 [0.73-1.73] | -0.07 [-1.92 to 1.81] |
| Equatorial Guinea | 0.02 [0.01-0.03] | 0.72 [0.45-1.06] | 0.07 [0.03-0.12] | 0.86 [0.41-1.48] | -0.05 [-1.97 to 1.91] |
| Angola | 0.52 [0.29-0.81] | 0.71 [0.46-1.05] | 1.44 [0.78-2.29] | 0.75 [0.37-1.26] | 0 [-1.8 to 1.83] |
| Central African Republic | 0.15 [0.09-0.23] | 0.8 [0.51-1.19] | 0.28 [0.16-0.42] | 0.79 [0.44-1.16] | -0.09 [-1.77 to 1.62] |
| Democratic Republic of the Congo | 1.66 [1.03-2.45] | 0.64 [0.41-0.94] | 3.76 [2.07-6.13] | 0.7 [0.36-1.19] | 0.26 [-1.56 to 2.12] |
| Pakistan | 11.93 [7.26-17.37] | 1.38 [0.87-1.94] | 24.87 [15.31-37.47] | 1.39 [0.89-2.05] | -0.17 [-2.02 to 1.72] |
| Burundi | 0.42 [0.26-0.63] | 0.99 [0.63-1.46] | 0.8 [0.4-1.35] | 0.97 [0.53-1.56] | -0.33 [-1.77 to 1.12] |
| Comoros | 0.03 [0.02-0.05] | 0.92 [0.6-1.35] | 0.06 [0.04-0.1] | 1.09 [0.69-1.71] | 0.16 [-1.45 to 1.8] |
| Djibouti | 0.02 [0.01-0.04] | 0.82 [0.49-1.3] | 0.09 [0.05-0.17] | 1.12 [0.61-1.9] | 0.72 [-0.88 to 2.36] |
| Kenya | 1.01 [0.65-1.48] | 0.71 [0.43-1.15] | 2.72 [1.69-4.1] | 0.9 [0.55-1.34] | 0.92 [-0.6 to 2.47] |
| Ethiopia | 13.36 [6.48-22.63] | 3.94 [2.36-6] | 19.28 [11.65-30.84] | 2.81 [1.69-4.72] | -1.61 [-3.36 to 0.17] |
| Eritrea | 0.21 [0.12-0.34] | 0.93 [0.56-1.42] | 0.48 [0.27-0.75] | 1.12 [0.65-1.65] | 0.47 [-0.99 to 1.96] |
| Gabon | 0.05 [0.03-0.07] | 0.72 [0.45-1.06] | 0.11 [0.06-0.18] | 0.89 [0.48-1.45] | 0.3 [-1.65 to 2.28] |
| Madagascar | 0.71 [0.46-1.08] | 0.77 [0.5-1.11] | 1.57 [0.91-2.44] | 0.83 [0.5-1.26] | 0.14 [-1.4 to 1.7] |
| Malawi | 0.28 [0.18-0.41] | 0.32 [0.21-0.46] | 0.42 [0.22-0.76] | 0.33 [0.19-0.54] | -0.19 [-1.73 to 1.36] |
| Seychelles | 0.02 [0.01-0.03] | 2.83 [1.93-4.08] | 0.03 [0.02-0.04] | 2.55 [1.67-3.62] | -0.15 [-1.47 to 1.2] |
| Mozambique | 1.64 [1.04-2.43] | 1.39 [0.9-2.03] | 3.06 [1.69-5.16] | 1.51 [0.93-2.32] | 0.48 [-1.09 to 2.08] |
| Mauritius | 0.15 [0.11-0.2] | 1.78 [1.25-2.34] | 0.28 [0.21-0.37] | 1.76 [1.3-2.3] | 0.79 [-1.28 to 2.91] |
| Rwanda | 0.6 [0.36-0.93] | 1.14 [0.7-1.7] | 0.96 [0.54-1.6] | 1.08 [0.63-1.76] | -1.08 [-2.52 to 0.37] |
| Somalia | 0.49 [0.27-0.82] | 1 [0.57-1.58] | 1.32 [0.76-2.1] | 1.15 [0.68-1.74] | 0.47 [-0.99 to 1.95] |
| United Republic of Tanzania | 1.98 [1.3-2.88] | 0.97 [0.63-1.4] | 4.44 [2.5-7.49] | 1.07 [0.63-1.76] | 0.14 [-1.41 to 1.7] |
| Zambia | 0.58 [0.36-0.9] | 0.97 [0.62-1.42] | 1.38 [0.8-2.13] | 1.15 [0.69-1.7] | -0.02 [-1.59 to 1.57] |
| Botswana | 0.08 [0.05-0.12] | 1.03 [0.62-1.58] | 0.18 [0.11-0.29] | 1.09 [0.65-1.7] | -0.04 [-2.58 to 2.58] |
| Uganda | 0.96 [0.59-1.39] | 0.72 [0.45-1.04] | 2.43 [1.25-4.19] | 0.82 [0.47-1.28] | 0.09 [-1.49 to 1.7] |
| Lesotho | 0.09 [0.05-0.13] | 0.86 [0.54-1.26] | 0.2 [0.12-0.29] | 1.51 [0.94-2.19] | 2.46 [-0.04 to 5.02] |
| Namibia | 0.07 [0.04-0.09] | 0.75 [0.5-1.06] | 0.15 [0.09-0.23] | 0.89 [0.56-1.34] | 0.06 [-2.48 to 2.67] |
| Eswatini | 0.05 [0.03-0.07] | 1.13 [0.74-1.71] | 0.1 [0.06-0.17] | 1.43 [0.84-2.29] | 0.85 [-1.61 to 3.37] |
| South Africa | 2.99 [1.94-4.07] | 1.14 [0.68-1.62] | 7.13 [4.32-10.01] | 1.46 [0.88-2.03] | 0.26 [-2.21 to 2.8] |
| Benin | 0.16 [0.1-0.24] | 0.37 [0.25-0.52] | 0.5 [0.22-0.81] | 0.49 [0.21-0.8] | 0.97 [-0.83 to 2.8] |
| Burkina Faso | 0.32 [0.19-0.46] | 0.38 [0.25-0.53] | 0.88 [0.38-1.4] | 0.51 [0.21-0.81] | 1.11 [-0.54 to 2.79] |
| Zimbabwe | 0.59 [0.38-0.82] | 1.06 [0.71-1.46] | 1.45 [0.89-2.16] | 1.48 [0.94-2.13] | 1.74 [-0.89 to 4.44] |
| Cameroon | 0.34 [0.22-0.5] | 0.41 [0.26-0.58] | 1.25 [0.56-2.07] | 0.56 [0.25-0.95] | 1.07 [-0.72 to 2.89] |
| Cabo Verde | 0.03 [0.02-0.04] | 0.87 [0.57-1.33] | 0.07 [0.04-0.11] | 1.51 [0.91-2.21] | 1.06 [-0.88 to 3.03] |
| Chad | 0.17 [0.11-0.25] | 0.31 [0.21-0.44] | 0.66 [0.33-1.04] | 0.5 [0.24-0.8] | 1.72 [0.02 to 3.45] |
| Ghana | 1 [0.47-1.55] | 0.82 [0.4-1.24] | 1.44 [0.87-2.28] | 0.55 [0.35-0.85] | -2.16 [-4.01 to -0.27] |
| Côte d'Ivoire | 0.27 [0.17-0.4] | 0.31 [0.19-0.45] | 0.64 [0.32-1.05] | 0.33 [0.17-0.52] | 0.1 [-1.7 to 1.93] |
| Gambia | 0.02 [0.01-0.02] | 0.2 [0.12-0.3] | 0.04 [0.02-0.06] | 0.24 [0.14-0.36] | 0.1 [-1.69 to 1.93] |
| Mali | 0.27 [0.16-0.4] | 0.33 [0.2-0.48] | 0.53 [0.28-0.91] | 0.3 [0.17-0.47] | -0.4 [-2.06 to 1.29] |
| Guinea | 0.1 [0.06-0.14] | 0.15 [0.09-0.22] | 0.16 [0.08-0.28] | 0.15 [0.08-0.24] | -0.01 [-1.75 to 1.75] |
| Guinea-Bissau | 0.04 [0.02-0.06] | 0.47 [0.28-0.72] | 0.08 [0.04-0.12] | 0.6 [0.29-0.9] | 0.96 [-0.69 to 2.64] |
| Liberia | 0.08 [0.05-0.13] | 0.37 [0.23-0.54] | 0.2 [0.09-0.32] | 0.53 [0.24-0.86] | 1.28 [-0.47 to 3.06] |
| Mauritania | 0.06 [0.04-0.08] | 0.36 [0.24-0.52] | 0.17 [0.07-0.27] | 0.53 [0.24-0.84] | 0.85 [-1.1 to 2.85] |
| Niger | 0.29 [0.17-0.44] | 0.37 [0.24-0.53] | 0.78 [0.33-1.4] | 0.44 [0.18-0.8] | 0.63 [-1.09 to 2.38] |
| Nigeria | 3.04 [1.88-4.65] | 0.39 [0.24-0.58] | 7.17 [3.54-11.45] | 0.4 [0.21-0.62] | -0.05 [-1.9 to 1.83] |
| Sao Tome and Principe | 0 [0-0] | 0.28 [0.18-0.39] | 0 [0-0.01] | 0.3 [0.19-0.48] | -0.03 [-1.8 to 1.77] |
| Senegal | 0.25 [0.17-0.36] | 0.38 [0.25-0.53] | 0.6 [0.26-1.02] | 0.52 [0.24-0.83] | 1.1 [-0.76 to 3] |
| Sierra Leone | 0.14 [0.08-0.21] | 0.35 [0.22-0.49] | 0.33 [0.14-0.53] | 0.49 [0.2-0.79] | 1.3 [-0.41 to 3.04] |
| Togo | 0.1 [0.07-0.14] | 0.36 [0.23-0.5] | 0.31 [0.13-0.49] | 0.54 [0.23-0.83] | 1.37 [-0.42 to 3.2] |
| Guam | 0.01 [0.01-0.02] | 1.24 [0.8-1.71] | 0.02 [0.01-0.02] | 0.95 [0.63-1.3] | -2.52 [-4.58 to -0.4] |
| Cook Islands | 0 [0-0] | 0.59 [0.36-0.85] | 0 [0-0] | 0.46 [0.28-0.68] | -2.27 [-4.24 to -0.25] |
| American Samoa | 0 [0-0.01] | 1.27 [0.84-1.8] | 0.01 [0-0.01] | 1.46 [0.95-2.08] | 0.38 [-2.43 to 3.27] |
| Bermuda | 0.02 [0.01-0.02] | 2.68 [1.9-3.53] | 0.03 [0.02-0.04] | 2.84 [1.94-4.06] | 0.5 [-0.82 to 1.83] |
| Monaco | 0.05 [0.03-0.08] | 11.82 [7.1-18.62] | 0.1 [0.06-0.14] | 14.86 [9.1-21.86] | 0.82 [-0.89 to 2.56] |
| Greenland | 0.01 [0-0.01] | 1.47 [0.98-2.13] | 0.01 [0-0.01] | 1.23 [0.79-1.77] | -0.51 [-1.82 to 0.82] |
| Nauru | 0 [0-0] | 1.73 [1.09-2.61] | 0 [0-0] | 1.59 [0.92-2.57] | -1.84 [-4.24 to 0.63] |
| Northern Mariana Islands | 0 [0-0.01] | 1.67 [0.93-2.47] | 0.01 [0-0.01] | 1.63 [0.91-2.38] | -0.47 [-4.32 to 3.53] |
| Puerto Rico | 0.88 [0.62-1.16] | 2.43 [1.71-3.21] | 1.5 [1.04-2.11] | 2.67 [1.85-3.75] | 0.22 [-1.04 to 1.49] |
| Saint Kitts and Nevis | 0.01 [0.01-0.01] | 2.28 [1.66-3.09] | 0.01 [0.01-0.02] | 2.01 [1.37-2.79] | 0.18 [-1.53 to 1.92] |
| Palau | 0 [0-0] | 1.07 [0.68-1.62] | 0 [0-0] | 1.06 [0.7-1.5] | 0.03 [-1.08 to 1.16] |
| Niue | 0 [0-0] | 1.18 [0.72-1.78] | 0 [0-0] | 1.85 [1.19-2.68] | -1.09 [-3.39 to 1.27] |
| Tokelau | 0 [0-0] | 1.25 [0.73-1.9] | 0 [0-0] | 2.01 [1.11-3.31] | -1.42 [-3.8 to 1.02] |
| San Marino | 0.04 [0.02-0.05] | 14.05 [9.22-20.88] | 0.04 [0.02-0.06] | 8.66 [4.94-13.49] | -0.77 [-2.72 to 1.23] |
| Tuvalu | 0 [0-0] | 1.4 [0.85-2.11] | 0 [0-0] | 1.17 [0.74-1.67] | -1.9 [-4.3 to 0.57] |
| South Sudan | 0.41 [0.24-0.68] | 0.91 [0.55-1.47] | 0.84 [0.5-1.28] | 1.26 [0.78-1.91] | 0.93 [-0.66 to 2.54] |
| United States Virgin Islands | 0.02 [0.01-0.03] | 1.99 [1.33-2.83] | 0.03 [0.02-0.04] | 1.78 [1.1-2.77] | -0.43 [-2.17 to 1.35] |
| Sudan | 3.53 [1.74-6.02] | 2.23 [1.26-3.44] | 6.91 [4.03-10.85] | 2.24 [1.3-3.49] | -0.11 [-1.33 to 1.13] |

| **Country** | **1990** | | **2021** | | **1990-2021** |
| --- | --- | --- | --- | --- | --- |
|  | **YLLs cases** | **YLLs per 100,000** | **YLLs cases** | **YLLs per 100,000** | **EAPC** |
|  | **No. *10^2^ (95% UI)** | **No. (95% UI)** | **No. *10^2^ (95% UI)** | **No.(95% UI)** | **No. (95% CI)** |
| China | 289.12 [191.52-402.08] | 2.73 [1.81-3.79] | 607.76 [378.58-861.8] | 4.16 [2.55-6.06] | -2.5 [-3.67 to -1.31] |
| Indonesia | 23.65 [14.17-35.27] | 1.63 [1-2.44] | 42.26 [27.64-60.82] | 1.66 [1.07-2.47] | -0.28 [-1.57 to 1.04] |
| Taiwan (Province of China) | 2.12 [1.49-2.82] | 1.18 [0.82-1.56] | 6.93 [4.9-9.42] | 2.28 [1.59-3.12] | 0.09 [-1.19 to 1.38] |
| Cambodia | 1.75 [0.88-2.86] | 2.33 [1.28-3.71] | 2.96 [1.93-4.39] | 2.05 [1.36-3.04] | -0.98 [-2.2 to 0.26] |
| Lao People's Democratic Republic | 0.74 [0.35-1.24] | 2.32 [1.18-3.73] | 1.02 [0.64-1.59] | 1.71 [1.06-2.68] | -1.38 [-2.65 to -0.11] |
| Democratic People's Republic of Korea | 3.6 [2.25-5.35] | 1.83 [1.15-2.69] | 5.83 [3.55-9.05] | 2.01 [1.23-3.09] | -0.52 [-1.67 to 0.65] |
| Philippines | 7.98 [5.38-11.19] | 1.58 [1.04-2.15] | 13.84 [9.22-18.94] | 1.39 [0.92-1.9] | -0.73 [-2.06 to 0.62] |
| Fiji | 0.08 [0.04-0.13] | 1.5 [0.76-2.26] | 0.11 [0.06-0.17] | 1.36 [0.72-2.06] | -1.35 [-3.59 to 0.93] |
| Thailand | 8.87 [5.75-12.35] | 2.03 [1.28-2.84] | 18.88 [11.77-27.28] | 2.28 [1.44-3.26] | -1.31 [-2.46 to -0.15] |
| Viet Nam | 6.11 [4.1-8.79] | 1.18 [0.8-1.73] | 11.39 [7.29-17.19] | 1.17 [0.75-1.77] | -0.94 [-2.79 to 0.94] |
| Maldives | 0.03 [0.01-0.04] | 1.72 [0.9-2.83] | 0.05 [0.03-0.07] | 1.19 [0.76-1.71] | -3.1 [-4.42 to -1.77] |
| Marshall Islands | 0 [0-0.01] | 1.28 [0.76-1.9] | 0.01 [0-0.01] | 1.28 [0.77-1.89] | -0.94 [-2.79 to 0.95] |
| Malaysia | 2.79 [1.92-3.85] | 2.1 [1.46-3.01] | 6.34 [4.29-9.62] | 2.12 [1.43-3.26] | -0.98 [-2.31 to 0.37] |
| Kazakhstan | 2.79 [1.94-3.66] | 1.89 [1.31-2.47] | 2.95 [2.1-4.06] | 1.58 [1.12-2.17] | -1.65 [-2.67 to -0.61] |
| Papua New Guinea | 0.32 [0.15-0.53] | 1.09 [0.5-1.9] | 0.77 [0.41-1.25] | 0.97 [0.5-1.66] | -1.7 [-3.68 to 0.32] |
| Timor-Leste | 0.1 [0.05-0.15] | 1.68 [0.99-2.45] | 0.17 [0.1-0.25] | 1.5 [0.94-2.26] | -1.09 [-2.4 to 0.23] |
| Solomon Islands | 0.03 [0.01-0.05] | 1.33 [0.56-2.19] | 0.06 [0.04-0.1] | 1.29 [0.72-1.97] | -1.39 [-3.22 to 0.48] |
| Mongolia | 0.2 [0.13-0.28] | 1.09 [0.73-1.55] | 0.28 [0.18-0.4] | 0.94 [0.6-1.36] | -1.12 [-2.41 to 0.19] |
| Vanuatu | 0.01 [0.01-0.02] | 1.16 [0.62-1.81] | 0.03 [0.02-0.04] | 1.1 [0.66-1.64] | -1.46 [-3.3 to 0.42] |
| Myanmar | 7.87 [3.62-13.15] | 2.39 [1.2-3.9] | 8.87 [5.62-13.8] | 1.7 [1.08-2.68] | -1.56 [-2.87 to -0.23] |
| Sri Lanka | 2.67 [1.76-3.68] | 1.94 [1.28-2.67] | 3.64 [2.15-5.95] | 1.49 [0.9-2.41] | -1.14 [-2.78 to 0.53] |
| Turkmenistan | 0.45 [0.32-0.59] | 1.44 [1-1.9] | 0.62 [0.42-0.88] | 1.29 [0.86-1.79] | -1.4 [-2.53 to -0.27] |
| Kiribati | 0.01 [0-0.01] | 0.94 [0.56-1.38] | 0.01 [0-0.01] | 0.93 [0.5-1.42] | -1.37 [-3.32 to 0.62] |
| Armenia | 0.84 [0.6-1.1] | 2.65 [1.9-3.49] | 0.93 [0.66-1.25] | 2.43 [1.73-3.23] | -1.69 [-2.62 to -0.74] |
| Azerbaijan | 1.25 [0.85-1.74] | 1.88 [1.29-2.61] | 1.74 [1.04-2.65] | 1.7 [1.03-2.57] | -1.2 [-2.39 to 0] |
| Samoa | 0.02 [0.01-0.03] | 1.74 [1.11-2.65] | 0.03 [0.02-0.04] | 1.59 [1.02-2.45] | -1.81 [-3.54 to -0.04] |
| Tonga | 0.01 [0-0.01] | 1.03 [0.61-1.59] | 0.01 [0.01-0.01] | 0.99 [0.63-1.52] | -1.55 [-3.24 to 0.16] |
| Tajikistan | 0.72 [0.47-1.03] | 1.53 [0.99-2.2] | 0.95 [0.55-1.59] | 1.05 [0.62-1.66] | -1.77 [-2.94 to -0.58] |
| Micronesia (Federated States of) | 0.01 [0.01-0.02] | 1.53 [0.95-2.28] | 0.01 [0.01-0.02] | 1.35 [0.86-2.03] | -1.76 [-3.59 to 0.1] |
| Kyrgyzstan | 0.69 [0.49-0.9] | 1.76 [1.24-2.29] | 0.75 [0.51-1.04] | 1.23 [0.85-1.71] | -2.23 [-3.18 to -1.26] |
| Croatia | 2.12 [1.49-2.86] | 3.66 [2.58-4.95] | 4.62 [3.27-6.14] | 5.61 [3.95-7.5] | -1.31 [-2.16 to -0.46] |
| Hungary | 4.94 [3.48-6.45] | 3.55 [2.52-4.57] | 8.01 [5.7-10.98] | 4.56 [3.27-6.33] | -1.41 [-2.28 to -0.52] |
| Poland | 13.28 [9.35-17.56] | 3.12 [2.19-4.12] | 28.99 [20.88-38.14] | 4.29 [3.11-5.67] | -1.93 [-2.74 to -1.11] |
| Romania | 5.07 [3.58-6.78] | 1.98 [1.39-2.64] | 9.85 [6.74-13.36] | 3.03 [2.08-4.14] | -1.14 [-2.09 to -0.18] |
| Slovakia | 1.91 [1.31-2.68] | 3.25 [2.25-4.58] | 3.87 [2.3-5.58] | 4.34 [2.63-6.21] | -1.45 [-2.49 to -0.4] |
| Ukraine | 21.94 [15.54-29] | 3.56 [2.53-4.68] | 17.83 [11.38-26.22] | 2.74 [1.78-3.94] | -2.06 [-2.94 to -1.18] |
| North Macedonia | 0.39 [0.25-0.53] | 2.06 [1.34-2.85] | 1 [0.57-1.5] | 3.12 [1.81-4.67] | -1.2 [-6.26 to 4.13] |
| Georgia | 1.6 [1.15-2.14] | 2.72 [1.95-3.64] | 1.35 [0.96-1.81] | 2.63 [1.86-3.51] | -1.34 [-2.32 to -0.35] |
| Russian Federation | 43.31 [30.86-56.64] | 2.55 [1.82-3.34] | 71.59 [51.71-93.53] | 3.35 [2.44-4.37] | -1.42 [-2.28 to -0.54] |
| Bosnia and Herzegovina | 0.68 [0.45-0.95] | 1.62 [1.1-2.29] | 1.6 [0.93-2.31] | 2.75 [1.63-3.96] | -0.88 [-1.92 to 0.17] |
| Latvia | 1.39 [0.98-1.82] | 4.2 [2.95-5.49] | 1.64 [1.16-2.22] | 4.64 [3.3-6.28] | -1.98 [-2.84 to -1.11] |
| Uzbekistan | 3.27 [2.35-4.39] | 1.9 [1.35-2.54] | 4.49 [3.08-6.39] | 1.39 [0.96-1.97] | -1.78 [-2.91 to -0.63] |
| Lithuania | 2.03 [1.46-2.66] | 4.76 [3.41-6.23] | 2.11 [1.51-2.88] | 4.17 [3-5.72] | -1.46 [-2.26 to -0.65] |
| Albania | 0.4 [0.26-0.56] | 1.57 [1.02-2.26] | 0.79 [0.44-1.44] | 2.17 [1.28-3.74] | -1.24 [-2.41 to -0.05] |
| Bulgaria | 2.39 [1.69-3.22] | 2.24 [1.58-2.99] | 3.27 [2.28-4.64] | 2.64 [1.82-3.76] | -0.68 [-1.62 to 0.28] |
| Montenegro | 0.19 [0.12-0.27] | 3.04 [1.91-4.33] | 0.37 [0.22-0.57] | 3.95 [2.37-6.03] | -0.94 [-1.99 to 0.13] |
| Estonia | 0.99 [0.72-1.31] | 5.15 [3.72-6.8] | 1.31 [0.91-1.82] | 5.39 [3.79-7.52] | -2.38 [-3.21 to -1.54] |
| Czechia | 4.96 [3.55-6.49] | 3.81 [2.76-4.94] | 10.9 [7.82-14.46] | 5.32 [3.8-7.13] | -1.46 [-2.32 to -0.58] |
| Serbia | 2.52 [1.69-3.47] | 2.45 [1.66-3.37] | 5.67 [3.49-8.12] | 3.62 [2.24-5.16] | -1.56 [-2.52 to -0.59] |
| Slovenia | 0.93 [0.66-1.21] | 3.95 [2.82-5.18] | 3.04 [2.1-4.23] | 7.31 [5.02-10.19] | -1.37 [-2.24 to -0.49] |
| Belarus | 4.65 [3.37-6.21] | 3.84 [2.78-5.12] | 6.97 [4.81-9.92] | 4.73 [3.32-6.7] | -1.87 [-2.71 to -1.02] |
| Japan | 38.21 [27.27-50.85] | 2.98 [2.13-4.02] | 65.43 [46.28-85.62] | 2.97 [2.15-3.94] | -1.79 [-3.2 to -0.35] |
| Republic of Korea | 6.52 [4.47-9.03] | 1.71 [1.17-2.43] | 12.74 [7.83-18.21] | 2.26 [1.29-3.35] | -3.41 [-4.78 to -2.02] |
| Andorra | 0.04 [0.02-0.07] | 7.98 [4.64-13.17] | 0.08 [0.05-0.14] | 6.81 [4.08-11.34] | -1.78 [-2.81 to -0.74] |
| France | 38.57 [27.87-51.14] | 5.26 [3.8-6.96] | 71.13 [51.82-96.29] | 6.01 [4.39-8.1] | -2.1 [-2.95 to -1.25] |
| Denmark | 4.19 [3.03-5.49] | 5.79 [4.17-7.58] | 6.87 [4.82-9.03] | 6.53 [4.59-8.63] | -1.99 [-2.89 to -1.08] |
| Belgium | 7.57 [5.46-10] | 5.8 [4.14-7.62] | 10.2 [7.51-13.37] | 5.39 [3.96-7.18] | -2.26 [-3.14 to -1.37] |
| Greece | 9.08 [6.63-11.87] | 7 [4.95-9.18] | 12.44 [8.93-16.4] | 6.44 [4.68-8.61] | -1.23 [-2.05 to -0.41] |
| Germany | 55.06 [39.82-71.08] | 5.02 [3.62-6.44] | 97.59 [70.05-130.97] | 6.07 [4.39-8.11] | -1.75 [-2.77 to -0.71] |
| Cyprus | 0.3 [0.2-0.47] | 3.93 [2.59-6.24] | 1.17 [0.67-1.68] | 6.24 [3.67-8.99] | -1.66 [-2.95 to -0.35] |
| Finland | 2.5 [1.78-3.29] | 3.81 [2.74-5.06] | 4.7 [3.38-6.18] | 4.56 [3.27-6.09] | -1.77 [-2.7 to -0.84] |
| Brunei Darussalam | 0.04 [0.03-0.05] | 2.31 [1.53-3.2] | 0.08 [0.05-0.11] | 1.97 [1.27-2.75] | -1.51 [-2.63 to -0.38] |
| Ireland | 2.07 [1.47-2.72] | 5.13 [3.67-6.78] | 3.43 [2.46-4.56] | 4.84 [3.44-6.45] | -2.2 [-3.17 to -1.22] |
| Malta | 0.13 [0.09-0.18] | 3.39 [2.39-4.54] | 0.28 [0.19-0.38] | 4.23 [2.95-5.94] | -1.93 [-2.99 to -0.85] |
| Norway | 2.15 [1.55-2.76] | 3.69 [2.68-4.8] | 4.12 [2.98-5.35] | 4.72 [3.46-6.13] | -1.61 [-2.7 to -0.51] |
| Spain | 24.81 [18.23-32.68] | 5.44 [3.95-7.32] | 41.92 [30.1-55.75] | 5.65 [4.13-7.64] | -2.3 [-3.21 to -1.39] |
| Luxembourg | 0.28 [0.2-0.37] | 5.86 [4.2-7.75] | 0.53 [0.39-0.74] | 5.68 [4.13-7.79] | -2.84 [-3.83 to -1.85] |
| Switzerland | 5.98 [4.27-7.89] | 7.2 [5.11-9.5] | 8.62 [6.13-11.43] | 5.87 [4.21-7.8] | -2.83 [-3.78 to -1.86] |
| Portugal | 4.12 [2.92-5.44] | 3.41 [2.42-4.51] | 8.55 [6.12-11.47] | 4.48 [3.23-5.96] | -2.32 [-3.35 to -1.28] |
| United Kingdom | 48.25 [35.24-62.66] | 6.14 [4.53-8] | 55.8 [40.59-71.74] | 4.97 [3.68-6.44] | -1.34 [-2.51 to -0.15] |
| Israel | 2.56 [1.86-3.31] | 5.15 [3.77-6.67] | 6.66 [4.83-8.85] | 5.6 [4.07-7.5] | -1.68 [-2.82 to -0.52] |
| Republic of Moldova | 0.99 [0.69-1.31] | 2.23 [1.55-2.93] | 0.87 [0.62-1.19] | 1.74 [1.23-2.39] | -2.19 [-3.37 to -1] |
| Netherlands | 9.77 [7.03-12.92] | 5.56 [4.02-7.31] | 13.12 [9.28-17.8] | 4.4 [3.11-5.96] | -2.08 [-3.08 to -1.08] |
| Chile | 2.1 [1.49-2.75] | 1.84 [1.3-2.42] | 4.85 [3.51-6.37] | 2.22 [1.59-2.96] | -1.6 [-2.63 to -0.55] |
| Sweden | 7.07 [5.09-9.05] | 5.88 [4.21-7.66] | 9.03 [6.57-12.24] | 4.9 [3.58-6.59] | -1.51 [-2.65 to -0.35] |
| Argentina | 7.4 [5.22-9.65] | 2.27 [1.6-2.96] | 11.06 [8.01-14.88] | 2.16 [1.56-2.9] | -1.17 [-2.15 to -0.18] |
| Uruguay | 1.04 [0.73-1.34] | 2.82 [2-3.67] | 1.72 [1.24-2.28] | 3.48 [2.52-4.58] | -0.9 [-1.76 to -0.03] |
| New Zealand | 2.25 [1.65-2.93] | 5.81 [4.27-7.58] | 4.92 [3.55-6.48] | 6.29 [4.56-8.28] | -0.99 [-2.12 to 0.15] |
| Canada | 21.81 [16.12-28.53] | 7.15 [5.28-9.38] | 35.09 [25.45-45.98] | 5.59 [4.13-7.37] | -1.86 [-2.84 to -0.88] |
| United States of America | 214.94 [157.31-276.72] | 7.08 [5.21-9.11] | 268.48 [197.6-343.13] | 5.09 [3.75-6.53] | -1.71 [-2.75 to -0.65] |
| Belize | 0.02 [0.02-0.03] | 1.37 [0.99-1.83] | 0.05 [0.04-0.07] | 1.55 [1.1-2.13] | -0.07 [-0.98 to 0.83] |
| Bahamas | 0.03 [0.02-0.04] | 1.59 [1.12-2.13] | 0.07 [0.05-0.09] | 1.67 [1.13-2.31] | -0.89 [-1.7 to -0.06] |
| Dominica | 0.01 [0.01-0.02] | 1.91 [1.33-2.66] | 0.02 [0.01-0.02] | 2.28 [1.48-3.32] | 0.41 [-0.62 to 1.44] |
| Antigua and Barbuda | 0.01 [0.01-0.01] | 1.53 [1.1-2.01] | 0.02 [0.01-0.02] | 1.61 [1.14-2.14] | -0.75 [-1.96 to 0.47] |
| Italy | 50.49 [36.48-65.33] | 7.15 [5.29-9.34] | 71.49 [52.46-92.99] | 6.41 [4.69-8.54] | -2.39 [-3.29 to -1.49] |
| Australia | 10.06 [7.28-13.11] | 5.32 [3.88-6.93] | 21.99 [15.82-28.68] | 5.39 [3.87-7.15] | -1.9 [-2.76 to -1.03] |
| Cuba | 2.33 [1.64-3.06] | 2.25 [1.58-2.95] | 3.73 [2.66-4.98] | 2.31 [1.66-3.1] | -1.25 [-2.29 to -0.19] |
| Singapore | 0.48 [0.33-0.63] | 1.92 [1.34-2.53] | 1.37 [0.98-1.82] | 2.44 [1.72-3.31] | -2.15 [-3.71 to -0.57] |
| Austria | 5.3 [3.87-6.93] | 5.1 [3.69-6.72] | 8.85 [6.34-11.44] | 5.54 [4-7.27] | -1.93 [-2.86 to -0.99] |
| Iceland | 0.14 [0.1-0.18] | 5.1 [3.6-6.64] | 0.25 [0.18-0.34] | 5.17 [3.65-7.04] | -1.58 [-2.58 to -0.57] |
| Barbados | 0.07 [0.05-0.09] | 2.34 [1.68-3.08] | 0.11 [0.07-0.15] | 2.42 [1.59-3.48] | -0.39 [-1.38 to 0.6] |
| Grenada | 0.02 [0.01-0.02] | 2.18 [1.52-2.82] | 0.03 [0.02-0.04] | 2.41 [1.73-3.27] | -0.76 [-1.46 to -0.05] |
| Suriname | 0.05 [0.03-0.06] | 1.45 [0.95-1.97] | 0.09 [0.06-0.12] | 1.43 [0.95-2.06] | -0.4 [-1.31 to 0.52] |
| Saint Lucia | 0.02 [0.01-0.03] | 1.93 [1.34-2.53] | 0.04 [0.03-0.05] | 1.73 [1.19-2.44] | -1.38 [-2.16 to -0.6] |
| Haiti | 1.35 [0.64-2.26] | 2.57 [1.44-3.99] | 2.13 [1.2-3.44] | 2.12 [1.26-3.33] | -0.7 [-1.71 to 0.32] |
| Ecuador | 1.35 [0.96-1.76] | 1.73 [1.22-2.26] | 3.71 [2.52-5.18] | 2.15 [1.47-3.01] | 0.54 [-0.71 to 1.81] |
| Dominican Republic | 0.85 [0.58-1.19] | 1.4 [0.95-1.96] | 1.45 [0.93-2.14] | 1.39 [0.89-2.04] | -0.66 [-1.81 to 0.49] |
| Trinidad and Tobago | 0.17 [0.13-0.23] | 1.76 [1.25-2.3] | 0.27 [0.18-0.39] | 1.62 [1.07-2.34] | -1.11 [-2.1 to -0.11] |
| Colombia | 5.09 [3.61-6.69] | 1.97 [1.38-2.58] | 10.99 [7.59-15.05] | 2.2 [1.52-3.04] | -1.17 [-2.31 to -0.01] |
| Saint Vincent and the Grenadines | 0.02 [0.01-0.03] | 2.38 [1.71-3.1] | 0.03 [0.02-0.04] | 2.19 [1.54-2.97] | -0.6 [-1.36 to 0.16] |
| Peru | 3.36 [2.21-4.71] | 1.86 [1.21-2.57] | 8.24 [5-12.08] | 2.38 [1.44-3.5] | -0.52 [-1.81 to 0.79] |
| Bolivia (Plurinational State of) | 1.41 [0.84-2.12] | 2.73 [1.67-4.02] | 2.5 [1.54-3.89] | 2.42 [1.5-3.77] | -1.02 [-2.24 to 0.22] |
| Jamaica | 0.36 [0.26-0.48] | 1.73 [1.24-2.28] | 0.58 [0.39-0.83] | 1.97 [1.31-2.81] | -0.23 [-1.36 to 0.92] |
| Nicaragua | 0.51 [0.34-0.72] | 1.44 [0.98-1.99] | 0.86 [0.57-1.22] | 1.48 [0.98-2.1] | -0.84 [-2.05 to 0.38] |
| Mexico | 12.27 [8.8-16.19] | 1.71 [1.21-2.22] | 24.22 [16.67-32.75] | 1.92 [1.33-2.61] | -1.1 [-2.43 to 0.24] |
| Guyana | 0.07 [0.05-0.09] | 1.21 [0.84-1.6] | 0.09 [0.06-0.13] | 1.26 [0.83-1.82] | 0.4 [-0.58 to 1.4] |
| El Salvador | 0.87 [0.6-1.21] | 1.94 [1.33-2.68] | 1.57 [1.03-2.28] | 2.49 [1.63-3.62] | -0.54 [-1.83 to 0.76] |
| Costa Rica | 0.53 [0.37-0.69] | 2.25 [1.59-2.93] | 1.55 [1.12-2.08] | 3.13 [2.26-4.21] | -0.43 [-1.54 to 0.69] |
| Guatemala | 0.92 [0.65-1.2] | 1.38 [0.96-1.79] | 2.27 [1.6-3.12] | 1.64 [1.15-2.26] | -0.26 [-1.58 to 1.07] |
| Honduras | 0.72 [0.48-1] | 1.82 [1.21-2.52] | 1.36 [0.85-2.08] | 1.76 [1.1-2.62] | -0.59 [-1.78 to 0.62] |
| Panama | 0.32 [0.23-0.42] | 1.54 [1.09-2.04] | 0.97 [0.65-1.39] | 2.28 [1.54-3.29] | -0.05 [-1.27 to 1.18] |
| Venezuela (Bolivarian Republic of) | 2.39 [1.67-3.17] | 1.55 [1.08-2.04] | 5.03 [3.3-7.33] | 1.84 [1.2-2.67] | -0.73 [-1.89 to 0.45] |
| Paraguay | 0.48 [0.32-0.67] | 1.49 [1-2.06] | 1.29 [0.83-1.88] | 2.07 [1.35-3.01] | 0.2 [-0.86 to 1.28] |
| Brazil | 17.9 [12.68-23.33] | 1.56 [1.09-2.04] | 38.96 [27.92-51.24] | 1.64 [1.18-2.17] | -1.1 [-2.16 to -0.04] |
| Algeria | 2.77 [1.79-3.99] | 1.45 [0.96-2.02] | 5.18 [3.28-7.71] | 1.39 [0.89-2.04] | -1.85 [-3.06 to -0.61] |
| Bahrain | 0.08 [0.05-0.11] | 3.29 [2.12-4.59] | 0.25 [0.16-0.38] | 2.57 [1.62-3.87] | -1.96 [-3.57 to -0.31] |
| Egypt | 6.63 [4.09-11.26] | 1.63 [0.96-2.88] | 21.64 [12.66-32.18] | 2.83 [1.73-4.11] | 0.52 [-0.51 to 1.56] |
| Iran (Islamic Republic of) | 12.07 [6.65-17.91] | 2.83 [1.49-4.08] | 23.81 [12.4-34.96] | 3.05 [1.58-4.47] | -1.46 [-2.44 to -0.47] |
| Iraq | 2.95 [1.86-4.74] | 2.24 [1.42-3.6] | 8.27 [5.18-12.25] | 2.71 [1.69-3.96] | -0.65 [-1.74 to 0.45] |
| Lebanon | 0.57 [0.32-0.95] | 2.42 [1.37-3.93] | 1.9 [1.19-2.83] | 3.25 [2.03-4.87] | -1.26 [-2.23 to -0.28] |
| Libya | 0.69 [0.45-0.97] | 2.47 [1.58-3.57] | 1.77 [1.13-2.7] | 3.19 [2.02-4.85] | -0.35 [-1.42 to 0.73] |
| Jordan | 0.58 [0.38-0.82] | 2.48 [1.65-3.5] | 2.27 [1.38-3.45] | 2.56 [1.56-3.91] | -1.7 [-2.97 to -0.41] |
| Kuwait | 0.32 [0.23-0.43] | 2.96 [2.15-3.91] | 0.76 [0.52-1.05] | 2.45 [1.64-3.41] | -2.37 [-3.45 to -1.28] |
| Morocco | 0.86 [0.55-1.24] | 0.44 [0.28-0.63] | 1.66 [1-2.47] | 0.48 [0.29-0.72] | -1.1 [-2.12 to -0.07] |
| Palestine | 0.54 [0.34-0.79] | 4.16 [2.62-5.88] | 1.29 [0.86-1.83] | 3.89 [2.61-5.46] | -1.3 [-2.27 to -0.33] |
| Oman | 0.2 [0.12-0.3] | 1.91 [1.11-2.81] | 0.5 [0.31-0.75] | 2.02 [1.28-2.97] | -1.67 [-2.7 to -0.64] |
| Qatar | 0.06 [0.04-0.09] | 3.55 [2.13-5.15] | 0.46 [0.26-0.74] | 3.56 [2.06-5.4] | -1.81 [-2.64 to -0.98] |
| Saudi Arabia | 1.19 [0.73-1.95] | 1.2 [0.72-2.07] | 5.97 [3.59-10.02] | 2.32 [1.42-3.64] | 0.42 [-0.41 to 1.26] |
| Syrian Arab Republic | 2.55 [1.67-3.71] | 2.99 [1.92-4.32] | 4.43 [2.65-6.9] | 3.34 [2.05-5.21] | -1.32 [-2.33 to -0.3] |
| Tunisia | 0.92 [0.6-1.33] | 1.4 [0.92-1.99] | 2.01 [1.1-3.2] | 1.62 [0.91-2.55] | -1.49 [-2.53 to -0.44] |
| United Arab Emirates | 0.25 [0.15-0.36] | 3.33 [2.01-4.92] | 1.3 [0.79-1.97] | 2.86 [1.8-4.19] | -0.92 [-1.78 to -0.05] |
| Yemen | 1.9 [1.01-3.18] | 2.12 [1.16-3.34] | 4.33 [2.22-6.97] | 2.08 [1.07-3.35] | -0.84 [-1.8 to 0.13] |
| Turkey | 13.97 [9.08-20.28] | 3.1 [1.98-4.4] | 28.67 [18.31-41.4] | 3.29 [2.13-4.75] | -2.28 [-3.4 to -1.14] |
| Afghanistan | 2.73 [1.21-4.61] | 3.36 [1.58-5.51] | 5.92 [3.04-9.66] | 3.28 [1.69-5.3] | -0.55 [-1.59 to 0.5] |
| Bangladesh | 13.01 [7.32-20.6] | 1.52 [0.95-2.23] | 16.51 [10.26-25.79] | 1.12 [0.69-1.74] | -1.53 [-2.98 to -0.06] |
| Bhutan | 0.06 [0.03-0.09] | 1.3 [0.74-2] | 0.08 [0.04-0.12] | 1.16 [0.7-1.89] | -0.73 [-2.26 to 0.82] |
| India | 77.56 [52.78-109.29] | 1.19 [0.8-1.64] | 134.99 [89.73-184.74] | 1.08 [0.71-1.48] | -0.85 [-2.16 to 0.48] |
| Congo | 0.12 [0.07-0.18] | 0.79 [0.51-1.13] | 0.3 [0.17-0.46] | 0.84 [0.46-1.28] | -0.45 [-1.83 to 0.94] |
| Nepal | 1.98 [1.13-3.19] | 1.31 [0.84-1.93] | 2.96 [1.9-4.55] | 1.14 [0.73-1.73] | -1.1 [-2.58 to 0.41] |
| Equatorial Guinea | 0.02 [0.01-0.03] | 0.72 [0.45-1.06] | 0.07 [0.03-0.12] | 0.86 [0.41-1.48] | -0.93 [-2.32 to 0.48] |
| Angola | 0.52 [0.29-0.81] | 0.71 [0.46-1.05] | 1.44 [0.78-2.29] | 0.75 [0.37-1.26] | -0.33 [-1.72 to 1.08] |
| Central African Republic | 0.15 [0.09-0.23] | 0.8 [0.51-1.19] | 0.28 [0.16-0.42] | 0.79 [0.44-1.16] | -0.04 [-1.36 to 1.29] |
| Democratic Republic of the Congo | 1.66 [1.03-2.45] | 0.64 [0.41-0.94] | 3.76 [2.07-6.13] | 0.7 [0.36-1.19] | -0.2 [-1.58 to 1.19] |
| Pakistan | 11.93 [7.26-17.37] | 1.38 [0.87-1.94] | 24.87 [15.31-37.47] | 1.39 [0.89-2.05] | 0.12 [-1.36 to 1.61] |
| Burundi | 0.42 [0.26-0.63] | 0.99 [0.63-1.46] | 0.8 [0.4-1.35] | 0.97 [0.53-1.56] | -0.46 [-1.42 to 0.5] |
| Comoros | 0.03 [0.02-0.05] | 0.92 [0.6-1.35] | 0.06 [0.04-0.1] | 1.09 [0.69-1.71] | -0.14 [-1.17 to 0.9] |
| Djibouti | 0.02 [0.01-0.04] | 0.82 [0.49-1.3] | 0.09 [0.05-0.17] | 1.12 [0.61-1.9] | 0.64 [-0.4 to 1.7] |
| Kenya | 1.01 [0.65-1.48] | 0.71 [0.43-1.15] | 2.72 [1.69-4.1] | 0.9 [0.55-1.34] | 0.37 [-0.55 to 1.29] |
| Ethiopia | 13.36 [6.48-22.63] | 3.94 [2.36-6] | 19.28 [11.65-30.84] | 2.81 [1.69-4.72] | -1.82 [-2.84 to -0.78] |
| Eritrea | 0.21 [0.12-0.34] | 0.93 [0.56-1.42] | 0.48 [0.27-0.75] | 1.12 [0.65-1.65] | 0.2 [-0.75 to 1.16] |
| Gabon | 0.05 [0.03-0.07] | 0.72 [0.45-1.06] | 0.11 [0.06-0.18] | 0.89 [0.48-1.45] | 0.06 [-1.35 to 1.49] |
| Madagascar | 0.71 [0.46-1.08] | 0.77 [0.5-1.11] | 1.57 [0.91-2.44] | 0.83 [0.5-1.26] | -0.24 [-1.24 to 0.78] |
| Malawi | 0.28 [0.18-0.41] | 0.32 [0.21-0.46] | 0.42 [0.22-0.76] | 0.33 [0.19-0.54] | -0.47 [-1.52 to 0.59] |
| Seychelles | 0.02 [0.01-0.03] | 2.83 [1.93-4.08] | 0.03 [0.02-0.04] | 2.55 [1.67-3.62] | -0.78 [-1.57 to 0.02] |
| Mozambique | 1.64 [1.04-2.43] | 1.39 [0.9-2.03] | 3.06 [1.69-5.16] | 1.51 [0.93-2.32] | -0.22 [-1.27 to 0.83] |
| Mauritius | 0.15 [0.11-0.2] | 1.78 [1.25-2.34] | 0.28 [0.21-0.37] | 1.76 [1.3-2.3] | 0.87 [-0.94 to 2.72] |
| Rwanda | 0.6 [0.36-0.93] | 1.14 [0.7-1.7] | 0.96 [0.54-1.6] | 1.08 [0.63-1.76] | -0.86 [-1.81 to 0.11] |
| Somalia | 0.49 [0.27-0.82] | 1 [0.57-1.58] | 1.32 [0.76-2.1] | 1.15 [0.68-1.74] | 0.23 [-0.69 to 1.15] |
| United Republic of Tanzania | 1.98 [1.3-2.88] | 0.97 [0.63-1.4] | 4.44 [2.5-7.49] | 1.07 [0.63-1.76] | -0.19 [-1.21 to 0.85] |
| Zambia | 0.58 [0.36-0.9] | 0.97 [0.62-1.42] | 1.38 [0.8-2.13] | 1.15 [0.69-1.7] | 0.14 [-0.88 to 1.16] |
| Botswana | 0.08 [0.05-0.12] | 1.03 [0.62-1.58] | 0.18 [0.11-0.29] | 1.09 [0.65-1.7] | -0.31 [-2.06 to 1.47] |
| Uganda | 0.96 [0.59-1.39] | 0.72 [0.45-1.04] | 2.43 [1.25-4.19] | 0.82 [0.47-1.28] | 0.15 [-0.82 to 1.13] |
| Lesotho | 0.09 [0.05-0.13] | 0.86 [0.54-1.26] | 0.2 [0.12-0.29] | 1.51 [0.94-2.19] | 1.83 [0.1 to 3.59] |
| Namibia | 0.07 [0.04-0.09] | 0.75 [0.5-1.06] | 0.15 [0.09-0.23] | 0.89 [0.56-1.34] | -0.27 [-2.02 to 1.5] |
| Eswatini | 0.05 [0.03-0.07] | 1.13 [0.74-1.71] | 0.1 [0.06-0.17] | 1.43 [0.84-2.29] | 0.75 [-0.96 to 2.49] |
| South Africa | 2.99 [1.94-4.07] | 1.14 [0.68-1.62] | 7.13 [4.32-10.01] | 1.46 [0.88-2.03] | -0.59 [-2.2 to 1.04] |
| Benin | 0.16 [0.1-0.24] | 0.37 [0.25-0.52] | 0.5 [0.22-0.81] | 0.49 [0.21-0.8] | 0.37 [-0.89 to 1.64] |
| Burkina Faso | 0.32 [0.19-0.46] | 0.38 [0.25-0.53] | 0.88 [0.38-1.4] | 0.51 [0.21-0.81] | 0.44 [-0.74 to 1.63] |
| Zimbabwe | 0.59 [0.38-0.82] | 1.06 [0.71-1.46] | 1.45 [0.89-2.16] | 1.48 [0.94-2.13] | 1.27 [-0.51 to 3.08] |
| Cameroon | 0.34 [0.22-0.5] | 0.41 [0.26-0.58] | 1.25 [0.56-2.07] | 0.56 [0.25-0.95] | 0.64 [-0.59 to 1.88] |
| Cabo Verde | 0.03 [0.02-0.04] | 0.87 [0.57-1.33] | 0.07 [0.04-0.11] | 1.51 [0.91-2.21] | 0.15 [-1.08 to 1.41] |
| Chad | 0.17 [0.11-0.25] | 0.31 [0.21-0.44] | 0.66 [0.33-1.04] | 0.5 [0.24-0.8] | 1.24 [0.01 to 2.47] |
| Ghana | 1 [0.47-1.55] | 0.82 [0.4-1.24] | 1.44 [0.87-2.28] | 0.55 [0.35-0.85] | -1.96 [-3.23 to -0.66] |
| Côte d'Ivoire | 0.27 [0.17-0.4] | 0.31 [0.19-0.45] | 0.64 [0.32-1.05] | 0.33 [0.17-0.52] | -0.03 [-1.24 to 1.19] |
| Gambia | 0.02 [0.01-0.02] | 0.2 [0.12-0.3] | 0.04 [0.02-0.06] | 0.24 [0.14-0.36] | 0.04 [-1.18 to 1.29] |
| Mali | 0.27 [0.16-0.4] | 0.33 [0.2-0.48] | 0.53 [0.28-0.91] | 0.3 [0.17-0.47] | -0.91 [-2.14 to 0.35] |
| Guinea | 0.1 [0.06-0.14] | 0.15 [0.09-0.22] | 0.16 [0.08-0.28] | 0.15 [0.08-0.24] | -0.48 [-1.77 to 0.82] |
| Guinea-Bissau | 0.04 [0.02-0.06] | 0.47 [0.28-0.72] | 0.08 [0.04-0.12] | 0.6 [0.29-0.9] | 0.37 [-0.8 to 1.55] |
| Liberia | 0.08 [0.05-0.13] | 0.37 [0.23-0.54] | 0.2 [0.09-0.32] | 0.53 [0.24-0.86] | 0.34 [-0.89 to 1.58] |
| Mauritania | 0.06 [0.04-0.08] | 0.36 [0.24-0.52] | 0.17 [0.07-0.27] | 0.53 [0.24-0.84] | 0.17 [-1.12 to 1.49] |
| Niger | 0.29 [0.17-0.44] | 0.37 [0.24-0.53] | 0.78 [0.33-1.4] | 0.44 [0.18-0.8] | -0.18 [-1.41 to 1.06] |
| Nigeria | 3.04 [1.88-4.65] | 0.39 [0.24-0.58] | 7.17 [3.54-11.45] | 0.4 [0.21-0.62] | -0.27 [-1.55 to 1.02] |
| Sao Tome and Principe | 0 [0-0] | 0.28 [0.18-0.39] | 0 [0-0.01] | 0.3 [0.19-0.48] | -0.32 [-1.6 to 0.98] |
| Senegal | 0.25 [0.17-0.36] | 0.38 [0.25-0.53] | 0.6 [0.26-1.02] | 0.52 [0.24-0.83] | 0.54 [-0.74 to 1.83] |
| Sierra Leone | 0.14 [0.08-0.21] | 0.35 [0.22-0.49] | 0.33 [0.14-0.53] | 0.49 [0.2-0.79] | 0.46 [-0.78 to 1.71] |
| Togo | 0.1 [0.07-0.14] | 0.36 [0.23-0.5] | 0.31 [0.13-0.49] | 0.54 [0.23-0.83] | 0.75 [-0.47 to 1.98] |
| Guam | 0.01 [0.01-0.02] | 1.24 [0.8-1.71] | 0.02 [0.01-0.02] | 0.95 [0.63-1.3] | -2.39 [-4.26 to -0.47] |
| Cook Islands | 0 [0-0] | 0.59 [0.36-0.85] | 0 [0-0] | 0.46 [0.28-0.68] | -3.3 [-5 to -1.57] |
| American Samoa | 0 [0-0.01] | 1.27 [0.84-1.8] | 0.01 [0-0.01] | 1.46 [0.95-2.08] | -0.06 [-2.15 to 2.08] |
| Bermuda | 0.02 [0.01-0.02] | 2.68 [1.9-3.53] | 0.03 [0.02-0.04] | 2.84 [1.94-4.06] | -2.27 [-3.27 to -1.26] |
| Monaco | 0.05 [0.03-0.08] | 11.82 [7.1-18.62] | 0.1 [0.06-0.14] | 14.86 [9.1-21.86] | -0.34 [-1.42 to 0.76] |
| Greenland | 0.01 [0-0.01] | 1.47 [0.98-2.13] | 0.01 [0-0.01] | 1.23 [0.79-1.77] | -1.78 [-2.8 to -0.75] |
| Nauru | 0 [0-0] | 1.73 [1.09-2.61] | 0 [0-0] | 1.59 [0.92-2.57] | -1.34 [-3.2 to 0.56] |
| Northern Mariana Islands | 0 [0-0.01] | 1.67 [0.93-2.47] | 0.01 [0-0.01] | 1.63 [0.91-2.38] | -0.83 [-3.8 to 2.23] |
| Puerto Rico | 0.88 [0.62-1.16] | 2.43 [1.71-3.21] | 1.5 [1.04-2.11] | 2.67 [1.85-3.75] | -1.35 [-2.35 to -0.34] |
| Saint Kitts and Nevis | 0.01 [0.01-0.01] | 2.28 [1.66-3.09] | 0.01 [0.01-0.02] | 2.01 [1.37-2.79] | -1.22 [-2.11 to -0.32] |
| Palau | 0 [0-0] | 1.07 [0.68-1.62] | 0 [0-0] | 1.06 [0.7-1.5] | -0.38 [-1.18 to 0.42] |
| Niue | 0 [0-0] | 1.18 [0.72-1.78] | 0 [0-0] | 1.85 [1.19-2.68] | -1.01 [-2.91 to 0.92] |
| Tokelau | 0 [0-0] | 1.25 [0.73-1.9] | 0 [0-0] | 2.01 [1.11-3.31] | -1.81 [-3.7 to 0.12] |
| San Marino | 0.04 [0.02-0.05] | 14.05 [9.22-20.88] | 0.04 [0.02-0.06] | 8.66 [4.94-13.49] | -1.74 [-2.67 to -0.81] |
| Tuvalu | 0 [0-0] | 1.4 [0.85-2.11] | 0 [0-0] | 1.17 [0.74-1.67] | -2.09 [-3.96 to -0.19] |
| South Sudan | 0.41 [0.24-0.68] | 0.91 [0.55-1.47] | 0.84 [0.5-1.28] | 1.26 [0.78-1.91] | 0.32 [-0.71 to 1.36] |
| United States Virgin Islands | 0.02 [0.01-0.03] | 1.99 [1.33-2.83] | 0.03 [0.02-0.04] | 1.78 [1.1-2.77] | -1.11 [-2.2 to -0.02] |
| Sudan | 3.53 [1.74-6.02] | 2.23 [1.26-3.44] | 6.91 [4.03-10.85] | 2.24 [1.3-3.49] | -0.84 [-1.91 to 0.24] |
